# From Sequence‐Defined Macromolecules to Macromolecular Pin Codes

**DOI:** 10.1002/advs.201903698

**Published:** 2020-03-03

**Authors:** Joshua O. Holloway, Filip Van Lijsebetten, Nezha Badi, Hannes A. Houck, Filip E. Du Prez

**Affiliations:** ^1^ Polymer Chemistry Research group (PCR) Centre of Macromolecular Chemistry (CMaC) Department of Organic and Macromolecular Chemistry Faculty of Sciences Ghent University Krijgslaan 281‐S4bis Ghent 9000 Belgium

**Keywords:** 1,2,4‐triazoline‐3,5‐dione, chemical encryption, multicomponent reactions, sequence‐defined macromolecules

## Abstract

Dynamic sequence‐defined oligomers carrying a chemically written pin code are obtained through a strategy combining multicomponent reactions with the thermoreversible addition of 1,2,4‐triazoline‐3,5‐diones (TADs) to indole substrates. The precision oligomers are specifically designed to be encrypted upon heating as a result of the random reshuffling of the TAD‐indole covalent bonds within the backbone, thereby resulting in the scrambling of the encoded information. The encrypted pin code can eventually be decrypted following a second heating step that enables the macromolecular pin code to be deciphered using 1D electrospray ionization‐mass spectrometry (ESI‐MS). The herein introduced concept of encryption/decryption represents a key advancement compared with current strategies that typically use uncontrolled degradation to erase and tandem mass spectrometry (MS/MS) to analyze, decipher, and read‐out chemically encrypted information. Additionally, the synthesized macromolecules are coated onto a high‐value polymer material, which demonstrates their potential application as coded product tags for anti‐counterfeiting purposes.

## Graphical abstract

Macromolecular pin codes are obtained through the combination of the dynamic Alder‐ene reaction of 1,2,4‐triazoline‐3,5‐dione (TAD) molecules with indole substrates and the Passerini three‐component reaction for the synthesis of tailor‐made, sequence‐defined macromolecules. The thermoreversible nature of the TAD‐indole reaction enables the encryption and decryption of the code and the read‐out is performed using electrospray ionization‐MS and liquid chromatography‐MS.

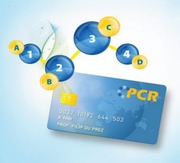

## 1 Introduction

In recent years, polymer chemists worldwide have been striving to prepare monodisperse synthetic macromolecules that possess properties mimicking the ones found in nature. This has resulted in the emergence of the field of sequence‐defined polymers.^[^ ^1^, ^2^, ^3^, ^4^, ^5^, ^6^, ^7^, ^8^, ^9^ ^]^ While until recently, research has been mostly dedicated toward the development of synthesis routes to design sequence‐defined macromolecules,^[^ ^1^, ^2^, ^3^ ^]^ attention is now shifting toward the potential use of these structures and their wider impact on our daily lives.^[^ ^10^, ^11^ ^]^ Hence, biomedical and biomimicking applications,^[^ ^12^, ^13^ ^]^ (bio)catalysis,^[^ ^14^ ^]^ long‐term data storage,^[^ ^6^, ^11^, ^15^, ^16^ ^]^ and product labeling for anti‐counterfeiting purposes^[^ ^17^ ^]^ are now all emerging. Information storage, in particular, is an area of increasing scientific interest as we move toward a more digital society, generating an ever‐increasing amount of data that will soon exceed the chip‐grade silicon resources required to store this.^[^ ^18^, ^19^ ^]^ As a result, polymer chemists are turning their interests toward a more compact and durable chemical solution.^[^ ^11^, ^20^ ^]^ While nonbiological information storage has already been successfully demonstrated using DNA,^[^ ^21^, ^22^ ^]^ along with its read‐out using DNA sequencing,^[^ ^23^ ^]^ there are drawbacks when considering this as a feasible data storage medium. These include, reliability as a result of possible degradation, a restricted number of building blocks (i.e., the monomer alphabet), limited rewritability, and cost.^[^ ^15^, ^20^, ^24^, ^25^ ^]^ In order to solve some of the drawbacks related to DNA, the use of discrete synthetic macromolecules has emerged as a viable alternative for data encoding at the molecular level and has the potential to greatly enhance data storage capacity.^[^ ^10^, ^11^, ^15^, ^16^, ^17^, ^20^, ^26^ ^]^

Although many protocols have been developed over recent years to store information on non‐natural sequence‐defined macromolecules,^[^ ^15^, ^16^, ^17^ ^]^ data encryption still remains an important aspect in future molecular data management, as individuals and enterprises seek more sophisticated and fraud‐resistant technologies.^[^ ^10^, ^17^ ^]^ To date, only a limited number of approaches using sequence‐defined macromolecules have been developed for the specific purpose of data encryption.^[^ ^16^, ^27^, ^28^ ^]^ For instance, Lutz and co‐workers reported the use of sequence‐defined poly(alkoxyamine amide)s for the encoding and decoding of information, which can be read‐out via tandem mass spectrometric (MS) analysis.^[^ ^27^ ^]^ Their study was completed by the total and uncontrolled erasing of the information using thermally induced degradation of the polymeric structure, which they claimed to be potentially easier to sequence than using biopolymers. Since then, the same group has expanded their research toward the elegant use of sequence‐defined (oligo)urethanes as readable, molecular barcodes to label polymer‐based materials for anti‐counterfeiting purposes.^[^ ^17^ ^]^ More recently, the use of photochemistry, as opposed to heat, has been used to introduce mutations in a sequence‐defined code, altering the information carried within.^[^ ^28^ ^]^

In a similar context, Meier and co‐workers reported the use of multicomponent reactions for the preparation of synthetic macromolecules for means of secret communication.^[^ ^16^ ^]^ To achieve this, molecular messages were deposited onto supports such as envelopes, from which they could be extracted and later deciphered using MS/MS. The fragmentation patterns were used to read out the information that could be translated into an alphanumeric code.^[^ ^16^ ^]^ Unlike alternative strategies, this was not limited to a simple binary code, but utilizing 130 commercially available components lead to up to 500 000 possible combinations. Finally, our own research group synthesized thiolactone‐based sequence‐defined oligomers, using an acrylate‐based alphabet to synthesize 71 different sequences in an automated fashion, which could be encoded and decoded to result in the unique storage of a QR code in a chemical form.^[^ ^15^ ^]^

When developing data management technologies, the reading part of the process is arguably more important than the writing and must be unambiguously demonstrated, which poses challenges to both data storage and encryption in order to retrieve molecularly encoded information in a fast, straightforward, and non‐destructive manner using readily available analytical techniques.^[^ ^10^ ^]^ Indeed, the current decryption of information‐containing macromolecules is mainly carried out using MS/MS,^[^ ^6^, ^10^, ^29^ ^]^ a technique that requires a high investment cost and relies on specific and readily fragmentable bonds in order to give distinct fragmentation patterns that can be identified and rationalized.^[^ ^30^ ^]^ For more complex encoded data, however, MS/MS sequencing rapidly requires additional—and often in‐house developed—computer algorithms.^[^ ^31^ ^]^

**Figure 1:**
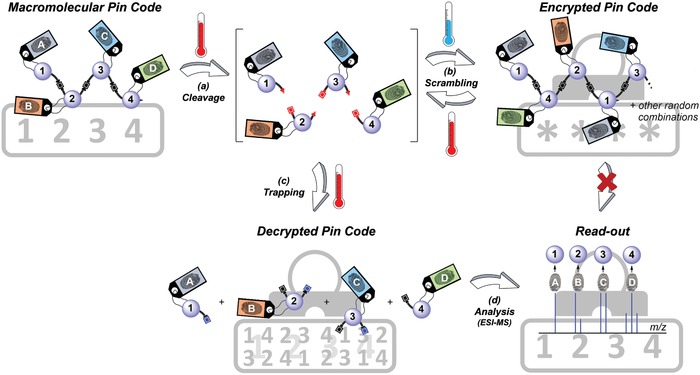
Schematic overview of the encryption and decryption of the macromolecular pin codes. a) The reversible TAD‐indole covalent bonds imbedded in the backbone of the pin codes enable cleavage of the digits upon heating. b) Upon cooling, the digits reconnect with one another in a random manner, thereby leading to a statistical mixture of unreadable oligomers. c) The encrypted pin code can only be decrypted upon subsequent heating in the presence of a trapping agent, which allows the monomer digits to separate from one another. d) The resulting mixture of monomers can eventually be read out via ESI‐MS analysis and their original order can be deciphered from the attached chemical fingerprints, hence revealing the initially written pin code.

Motivated by the need for both a more advanced encryption and a simplified read‐out strategy, we herein introduce cleavable covalent linkages into the backbone of sequence‐defined oligomers that exhibit a reliable and clean dynamic behavior at elevated temperatures. Specifically, we aimed to synthesize macromolecules bearing a numeric code by using the thermoreversible reaction of 1,2,4‐triazoline‐3,5‐dione (TAD) coupling reagents with indole substrates.^[^ ^32^, ^33^, ^34^ ^]^ This was used in combination with the Passerini three‐component reaction (P‐3CR), which is the one‐pot reaction between a carboxylic acid, aldehyde or ketone, and isocyanide (**Figure** **1**).^[^ ^35^ ^]^ In contrast to the thermal unzipping of sequences that requires high degradation temperatures,^[^ ^23^ ^]^ the bond scission profile of TAD‐indole linkages is accessible at a much milder temperature range (i.e., 60–150 °C). This guarantees cleavage to occur in a reliable manner at specific sites along the backbone, thereby resulting in simplified structural fragments that can be decrypted by 1D MS techniques. Moreover, the dynamic nature also offers the potential to scramble the TAD‐indole combinations, allowing the encryption of the molecularly encoded information. While the word “encryption” generally means “to convert information (…) into a secret code that people cannot understand or use on normal equipment,”^[^ ^36^ ^]^ we are more specifically referring to encryption as the concealment of the numeric code in the context of this study.

Following our interests in the synthesis of precise sequence‐defined macromolecules, the aim of this research was, to demonstrate our encryption/decryption protocol through the design of macromolecular pin codes whereby chemical digits (e.g., 1–4, Figure 1) were written in a defined order using commercially available P‐3CR building blocks. Importantly, tailored indole compounds, bearing a distinct isotopic pattern, were simultaneously introduced during the writing process in order to mark the digit's position within the sequence (i.e., A to D) and enable easier read‐out. As the digits are connected through the thermally dynamic TAD‐indole linkages, the written pin codes can readily be dissociated upon heating (Figure 1a) and subsequently transformed into a statistical distribution of oligomers upon cooling to room temperature (Figure 1b), resulting in unreadable information. Additionally, the individual monomer digits could later be isolated by heating in the presence of a kinetic trap (Figure 1c) and the information could eventually be retrieved by means of electrospray ionization‐MS (ESI‐MS) analysis, based on their difference in mass (Figure 1d). The original position of the digit in the macromolecular pin code could hence be deciphered through the attached “chemical fingerprints” of the indole compounds. In other words, we demonstrate that only those who know the initial order of the indole marker compounds (here ABCD) are hence able to decipher the encoded information and thus recover the original pin code (thereby for instance differentiating between 1234 and 4321).

## 2 Results and Discussion

### 2.1 Writing Macromolecular Pin Codes

**Figure 2:**
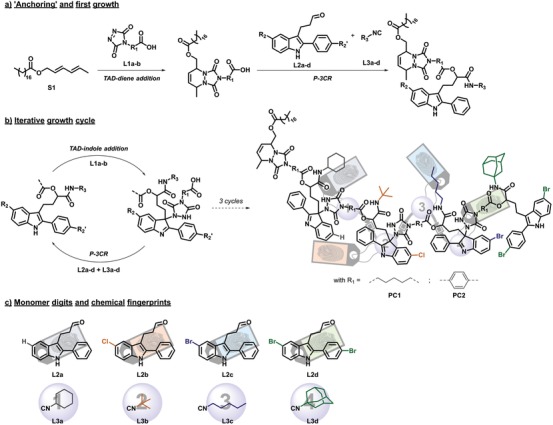
Chemical protocol combining the P‐3CR and thermoreversible TAD‐indole addition reaction to write sequence‐defined macromolecules. a) Initial Diels–Alder reaction between diene **S1** and TAD‐COOH **L1a‐b**, followed by the P‐3CR with an indole aldehyde (**L2a‐d**) and isocyanide (**L3a‐d**) in order to encode the first position marker (A) and digit (1), respectively. b) Two‐step iterative growth cycle, resulting in the macromolecular pin codes **PC1** and **PC2**, synthesized by using the aliphatic and aromatic TAD‐linkers **L1a** and **L1b**, respectively. c) Overview of the indole position markers and isocyanide digits used as the library to construct the pin codes.

Prior to evaluating our above outlined encryption/decryption strategy (see Figure 1; Movie S1, Supporting Information), we first identified a chemical procedure to write TAD‐indole‐based molecular pin codes that could undergo the desired reversible transformations. For this, a recently established iterative synthesis protocol that combines the reactivity of TADs with the P‐3CR multicomponent reaction was modified in order to implement the required thermoreversible linkages within the sequence.^[^ ^37^ ^]^ Indeed, changing one of the reagents from a conjugated diene to an indole‐containing moiety would alter the nature of the formed covalent TAD linkage within the sequence from an irreversible to a thermally reversible one.^[^ ^34^ ^]^ Thus, the TAD carboxylic acid (TAD‐COOH) building blocks (**L1a**‐**b**, **Figure** **2**) were combined with four different indole‐aldehyde analogues (**L2a‐d**) to act as dynamic TAD reaction partners.^[^ ^37^ ^]^ Furthermore, **L2b‐d** contained Cl, Br, and 2xBr substitutions in order to introduce specific isotopic patterning to the macromolecular pin codes. In addition, structurally diverse and commercially available isocyanide compounds (**L3a‐d**) were selected to serve as the third component in the P‐3CR.

**Figure 3:**
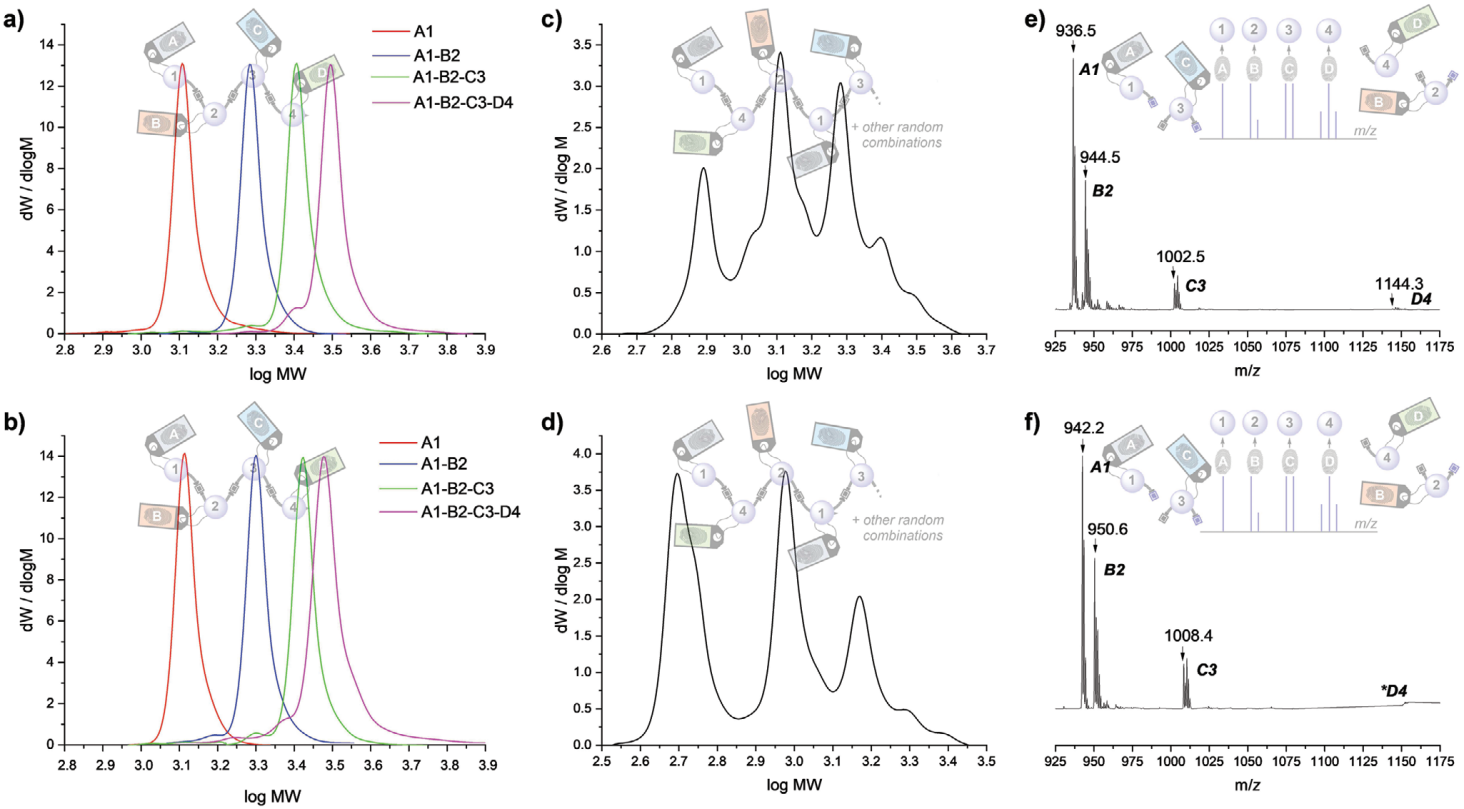
a,b) SEC elugrams recorded during the synthesis of **PC1** and **PC2**, respectively, showing a progressive increase in molecular weight from monomer (A1) to tetramer (A1‐B2‐C3‐D4). Purities of the isolated sequences are given in Table S2, Supporting Information. c,d) SEC elugrams of **PC1** and **PC2** following heating for 15 min at 150 and 120°C, respectively, to encrypt the pin code. e,f) ESI‐MS read‐out after decryption of **PC1 and PC2**, respectively. *A zoom of the ESI‐MS spectrum showing **D4** is given in Figure S11, Supporting Information.

Following the synthesis of the necessary building blocks, a macromolecular pin code was next written via the two‐step iterative protocol depicted in Figure 2. It should be noted that the initial Diels–Alder reaction of **L1a** or **L1b** with the apolar starting block **S1** (Figure 2a) was merely a practical consideration, implemented to assist in the purification process. Following the iterative‐growth cycle, the first macromolecular pin code (tetramer) **PC1** (using the aliphatic TAD‐COOH **L1a**) was eventually obtained as a powder. The four isocyanide compounds incorporated throughout the sequence were used to chemically write the numeric pin code of 1‐2‐3‐4 and their order A‐B‐C‐D in the macromolecular structure was denoted by the substituted indole‐aldehyde analogues **L2a‐d** that they were paired with. The sequential growth of the macromolecular pin code **PC1** was monitored after each writing cycle through analysis by means of SEC (see **Figure** **3**a), as well as by liquid chromatography mass spectrometry (LCMS) and NMR (see Supporting Information). Further analysis of the resulting macromolecular pin code via matrix‐assisted laser desorption/ionization tandem mass spectrometry (MALDI‐MS/MS) showed that the original order of the sequence could still be determined (Figure S1, Supporting Information).

### 2.2 Reversibility Model Studies of Macromolecular Pin Codes

Following the synthesis, characterization, and read‐out of **PC1**, the concealment of the sequential order of the macromolecular pin code was evaluated by further investigation of the thermoreversible TAD‐indole chemistry. For this, kinetic reversibility experiments of **PC1** (0.02 M in DMSO‐*d*_6_) were first conducted at three distinct temperatures (i.e., 120, 135, and 150 °C) in the presence of a slight excess of a low molecular weight conjugated diene (i.e., (*E*,*E*)‐2,4‐hexadien‐1‐ol). The latter served as an irreversible and kinetically favored trap, forming an irreversible TAD–Diels–Alder adduct, effectively quenching any released TAD as the pin code was subjected to elevated temperatures. Thus, the fraction of TAD‐indole dissociation could be closely monitored over time through offline ^1^H NMR measurements (Figure S3, Supporting Information).^[^ ^32^, ^33^ ^]^ Several key proton resonances indeed provided the confirmation of the successful TAD‐indole dissociation, thus demonstrating the successful deconstruction of the sequence‐defined tetramer.

As expected, the temperature had a significant effect on the rate of oligomer dissociation, with complete conversion to the diene‐capped monomers—and hence full dissociation of the pin code—reached within 15 min at 150 °C, 45 min at 135 °C, and 180 min at 120 °C (Figure S4, Supporting Information). Moreover, an observed activation energy *E*_a,obs_ = 116.1 ± 8.5 kJ mol^−1^ could be derived from the kinetic study (Table S1, Supporting Information), which is in accordance with the values of previous studies on simple low molecular weight TAD‐indole systems (see Supporting Information for details).^[^ ^32^, ^33^ ^]^

While it was demonstrated that the reversibility is relatively fast at 150 °C, this high temperature is often regarded as the upper limit at which reliable TAD‐indole exchange reactions can occur without significant TAD‐degradation.^[^ ^38^ ^]^ In order to anticipate this potential issue, a second macromolecular pin code (**PC2**) was synthesized using the alternative aromatic TAD‐COOH derivative **L1b**, as this was previously reported to significantly reduce the temperature of reversibility of the corresponding indole adduct compared to its aliphatic counterpart.^[^ ^32^, ^33^ ^]^ Similar to the first pin code, **PC2** was again characterized using SEC (Figure 3b), LCMS, and NMR (see Supporting Information) during the pin code writing process and complemented by MALDI‐MS/MS to retrieve the encoded information (see Figure S2, Supporting Information). The thermal reversibility kinetics was also investigated by offline ^1^H NMR (Figure S5, Supporting Information). As anticipated, complete dissociation of **PC2** was now observed to be completed in a faster timescale, that is, after 15 min at 120 °C (Figure S6, Supporting Information), with an observed activation energy *E*_a,obs_ = 95.7 ± 5.3 kJ mol^−1^ (Table S1, Supporting Information). Besides a significant acceleration of the encryption process, these results highlight the potential to further tune the thermoreversible properties by changing the structure of the TAD derivative.

### 2.3 Encryption of Macromolecular Pin Codes

Following the kinetic studies designed to gain basic insights into the thermal deconstruction process of the pin codes, the encryption process—whereby the written information is (temporarily) secured—was evaluated. In this context, both **PC1** and **PC2** were heated for 15 min at 150 and 120 °C, respectively, this time in the absence of any co‐reactant. During this heating step, the retro‐reaction of the TAD‐indole covalent bonds was initiated, which led to the complete dissociation of the monomer units from the oligomer (see Figure 1a). Since no trapping agent was present during this encryption stage, the in situ liberated TAD moieties were no longer scavenged from the reaction mixture and consequently allowed the TAD‐indole systems to exchange with one another. Subsequent cooling to room temperature eventually resulted in a recombination of the covalent bonds, albeit in a completely random order (see Figure 1b). Hence, a statistical mixture of disperse oligomers was formed, as evidenced by the SEC elugrams (Figure 3c,d), confirming the successful encryption of the macromolecular pin codes. Moreover, MALDI‐MS/MS analysis of the encrypted pin codes showed that the original order of the sequence could no longer be determined, as different dimer combinations were observed, which resulted in ambiguity during read‐out (Figures S7 and S8, Supporting Information). Additionally, the mixture proved to be unreadable by means of ESI‐MS (Figures S9 and S10, Supporting Information), although the Diels–Alder adduct formed as a result of starting with **S1**, thus encoding A1, could be identified from the ESI‐MS spectrum. The sacrificial identification of the first digit during encryption could be avoided, however, by either using an indole instead of the conjugated diene to initiate the sequence growth, or by first incorporating a non‐information containing dummy digit.

### 2.4 Decryption and Read‐Out of Macromolecular Pin Codes

In a final stage, we aimed to retrieve the encrypted information embedded in the macromolecular pin codes. As with the encryption studies, a heat treatment was carried out on **PC1** and **PC2** (at 120 °C for 5 and 2 h, respectively), but this time in the presence of the conjugated diene trapping agent **S1**, which was also used as the anchor from which the iterative sequence‐defined growth was initiated. As the TAD‐indole bonds of the unreadable oligomeric mixture opened at elevated temperature, the sequence could now be effectively dissociated into its separate monomer units (see Figure 1c). When cooling the mixture in the presence of **S1**, the Diels–Alder reaction between the newly liberated TAD moieties and conjugated diene was kinetically favored over the recombination reaction with the indole compounds, which is known from our previous research.^[^ ^33^ ^]^ In other words, **S1** acted as a kinetic trap to isolate the monomer units.

In contrast to the previously obtained disperse statistical distribution of oligomers, the resulting mixture of defined monomer fragments could be readily identified by means of LCMS and ESI‐MS (Figures S12 and S13, Supporting Information). The initial position of the different monomer digits could be deciphered using the chemical fingerprint associated with their corresponding substituted indole markers (Figure S14, Supporting Information). Indeed, by making use of the unique isotopic patterns, the related *m*/*z* values could be linked to the correct chemical structure of the digits based on their difference in mass. As a result, the different positions and contained information of the digits could be acquired and the molecular pin codes deciphered, thereby retrieving the initial sequencing order (Figure 3e,f). Note, the ESI‐MS can be complemented with LCMS when poor ionization of the cleaved monomer fragments is observed. It should be also noted that if (part of) the pin code is revealed, the synthesis of a new macromolecular pin code would be required.

### 2.5 Anti‐Counterfeiting Demonstration

Among the many emerging applications of sequence‐defined macromolecules is their use as chemical labels for anti‐counterfeiting purposes.^[^ ^17^ ^]^ To demonstrate the use of our conceptual encryption/decryption approach in this context, the encrypted oligomers were deposited onto biaxially oriented polypropylene banknotes, to add a concealed chemical label as an additional layer of security. In general, such polymer‐based banknotes contain multiple layers of printing and security features, which are ever‐changing and evolving to protect the currency from counterfeiting.

**Figure 4:**
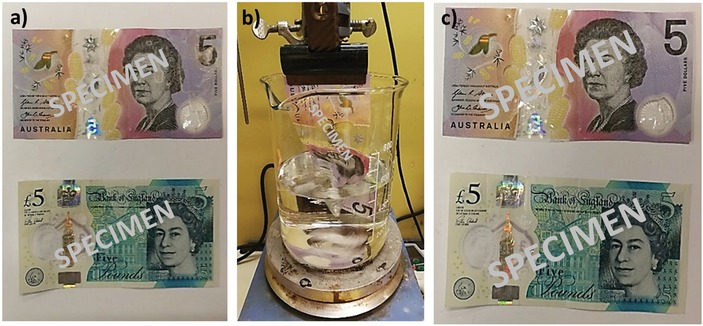
Macromolecular tagging of banknotes for anti‐counterfeiting. a) $5 and £5 banknotes coated with encrypted pin codes **PC1** and **PC2**, respectively. b) Suspension of the banknotes in ethanol allows for the extraction of the encrypted macromolecular tag and their subsequent decryption and read‐out (here shown for the $5). c) Banknotes obtained after extraction of the macromolecular tags.^[^ ^39^ ^]^

Thus, as a proof‐of‐concept study, a £5 Bank of England and a $5 Reserve Bank of Australia polymer banknote (**Figure** **4**a), which had been in circulation and hence subjected to the usual mechanical wear of daily use, were labeled, respectively, with the encrypted **PC1** and **PC2**. Prior to this, the banknotes were suspended overnight in ethanol to verify that they remained unaltered in the solvent (Figure S15, Supporting Information). Furthermore, the products extracted from the banknote during suspension were analyzed by ESI‐MS in order to record a background reference (see Figure S16, Supporting Information). Ethanol was chosen here as non‐toxic solvent, which could be used to both deposit and extract the chemical information. Approximately 10 mg of encrypted **PC1** and **PC2** was dissolved in a minimal amount of ethanol and deposited on the surface of the respective banknotes. Mild heating (<50 °C) was applied to evaporate the solvent and the notes were then left to dry overnight. To the casual eye, the encoded banknotes had the same appearance as the pristine ones (Figure 4a; Figure S15, Supporting Information). They were placed again in ethanol for 5 h to re‐extract the encrypted pin codes from the surface (Figure 4b,c). Solvent removal in vacuo allowed for the recovery of the encrypted codes, which were finally decrypted by heating in butyl acetate (4 h at 120 °C) in the presence of the trapping agent **S1**. Eventually, the initially written pin codes were successfully deciphered using ESI‐MS, following the above‐outlined strategy (see Figures S17 and S18, Supporting Information).

## 3 Conclusion

In this work, the successful combination of the P‐3CR and TAD‐indole chemistry was demonstrated for the synthesis of dynamic sequence‐defined macromolecules. The thermoreversible TAD‐indole reaction was utilized to construct, scramble, and deconstruct well‐defined macromolecular structures, carrying a molecular pin code. The introduction of a range of indole‐aldehyde analogues meant that the original order of the sequence could be readily deciphered and read‐out through distinct isotopic patterning (or “chemical fingerprinting”) using ESI‐MS, rather than the currently employed sophisticated tandem mass analyses. Such tandem mass analysis was used in this study for the sole purpose of unambiguously proving the necessity of the thermal encryption for the complete concealment of the original sequence order following scrambling. By altering the structure of the TAD‐COOH compound, we were able to tune the temperature of reversibility to conduct both the encryption and decryption process. Finally, we visually demonstrated how this conceptual approach could potentially be used to further advance the use of precision chemistry for anti‐counterfeiting applications. Currently, the use of four different isocyanides allows for 4^4^ (256) possible combinations, which translates to a small storage capacity of 8 bits. Our strategy can, however, readily be expanded to a wide range of other isocyanide compounds, thereby significantly extending the amount of codable pin code digits and hence increase the amount of possible permutations to *n* ^4^. The implementation of a reliable, dynamic covalent chemistry within sequence‐defined macromolecules could be considered as an important advancement in the development of the next generation of encryption and decryption technologies.

## Conflict of Interest

The authors declare no conflict of interest.

## Supporting information

Supporting Information

Supplemental Movie 1
