# Supplementary material for: From Sequence‐Defined Macromolecules to Macromolecular Pin Codes
Source: Adv Sci (Weinh). 2020 Mar 3;7(8):1903698. doi: 10.1002/advs.201903698 (PMC7175230; doi:10.1002/advs.201903698)
Supplement: Supplementary file 1 — Supporting Information [file ADVS-7-1903698-s001.pdf]

Copyright WILEY-VCH Verlag GmbH & Co. KGaA, 69469 Weinheim, Germany, 2020.

ADVANCED SCIENCE
Open Access

Supporting Information

for *Adv. Sci.,* DOI: 10.1002/advs.201903698

From Sequence-Defined Macromolecules to Macromolecular Pin Codes

*Joshua O. Holloway, Filip Van Lijsebetten, Nezha Badi, Hannes A. Houck, and Filip E. Du Prez**

Supporting Information

From Sequence-Defined Macromolecules to Macromolecular Pin Codes

*Joshua O. Holloway, Filip Van Lijsebetten, Nezha Badi, Hannes A. Houck and Filip E. Du Prez**

Dr J. O. Holloway, F. Van Lijsebetten, Dr N. Badi, Dr H. A. Houck and Prof. Dr F. E. Du Prez
Polymer Chemistry Research group (PCR), Centre of Macromolecular Chemistry (CMaC), Department of Organic and Macromolecular Chemistry, Faculty of Sciences, Ghent University, Krijgslaan 281-S4bis, Ghent, 9000, Belgium
E-mail: Filip.DuPrez@UGent.be

Table of contents

1. Instrumentation S3

2. Materials S5

3. Synthetic procedures S6

3.1 Synthesis of triazolinedione carboxylic acids **L1a-b** S6

3.1.1 Synthesis of 6-(3,5-dioxo-3,5-dihydro-4H-1,2,4-triazol-4-yl)hexanoic acid (**L1a**) S6

3.1.2 Synthesis of 4-(3,5-dioxo-3,5-dihydro-4H-1,2,4-triazol-4-yl)benzoic acid (**L1b**) S8

3.2 Synthesis of substituted 2-phenyl-1*H*-indole aldehydes **L2a-d** S10

3.2.1 Synthesis of substituted 2-phenyl-1H-indole precursors (**l2b-d**) S10

3.2.2 Synthesis of substituted 2-phenyl-1H-indole aldehydes **L2a-d** S12

3.3 Synthesis of macromolecular pin codes **PC1** and **PC2** S15

3.3.1 Synthesis of (2E,4E)-hexa-2,4-dien-1-yl stearate (**S1**) S15

3.3.2 Synthesis of aliphatic TAD-indole-based pin code (**PC1**) S16

3.3.3 Synthesis of aromatic TAD-indole-based pin code (**PC2**) S21

4. MALDI-MS/MS analysis of **PC1** and **PC2** S26

5. Encryption model study S28

6. Decryption model study S28

7. MALDI-MS/MS analysis of **PC1** and **PC2** after encryption S34

8. ESI-MS analysis of the encryption of **PC1** and **PC2** S36

9. Decryption and read-out of **PC1** and **PC2** S38

10. Proof-of-concept demonstration on polymer banknotes S42

11. Supplementary LCMS Figures S44

12. Supplementary NMR Figures S45

13. References S59

1. Instrumentation

Electrospray Ionization Mass Spectrometry (ESI-MS) and Liquid Chromatography Mass Spectrometry (LCMS). An Agilent technologies 1100 series LC/MSD system equipped with a diode array detector and single quad MS detector (VL) with an electrospray source (ESI-MS) was used for classic reversed phase LCMS and MS analysis. Analytic reversed phase HPLC (high-performance liquid chromatography) was performed using a Cyano porous column with a flow rate of 1 mL min^{-1} (5µm, 250 x 4.6 mm) using a solvent gradient of 50 → 100 % acetonitrile in water over 15 minutes, or a Phenomenex Luna C5 column (5µm, 250 x 4.6 mm) using a solvent gradient of 90 → 100 % acetonitrile in water over 30 minutes. The eluting compounds were detected via UV-detection (λ = 214 nm). LCMS spectra were analyzed using ACD/Spectrus software and the purity of compounds was assessed by integrating the LC peaks (λ = 214 nm) using the in-software function.

Nuclear magnetic resonance (NMR) spectroscopy. NMR spectra were recorded on a Bruker Avance 300 (300 MHz), Bruker Ascend 400 (400 MHz) or Bruker Avance II (500 MHz) FT-NMR spectrometer at 25 °C in either $CDCl_3$ or DMSO-d_6, as indicated. Chemical shifts (δ) are expressed in parts per million (ppm) whereby the residual solvent peaks ($CDCl_3$: ^1H = 7.26 ppm and ^{13}C = 77.16 ppm; DMSO-d_6: ^1H = 2.50 ppm and ^{13}C = 39.52 ppm) served as an internal standard. Coupling constants (J) are reported in Hertz (Hz). The resonance multiplicities are abbreviated as follows: s (singlet), d (doublet), t (triplet), q (quadruplet), quint (quintet), sext (sextet) or m (multiplet). Standard parameters in terms of number of scans, pulse delay, acquisition time, tilt angle and pulse time:
^1H spectra (300 MHz: ns = 16, D1 = 1.0 s, AQ = 2.65 s, 30°, P1 = 7.25 µs; 400 MHz: ns = 16, D1 = 1.0 s, AQ = 4.10 s, 30°, P1 = 7.75 µs; 500 MHz: ns = 16, D1 = 1.0 s, AQ = 3.28 s, 30°, P1 = 13.83 µs); ^{13}C spectra (75 MHz: ns = 3072, D1 = 2.0 s, AQ =1.82 s, 30°, P1 = 7.50 µs; 100 MHz: ns = 3072, D1 = 2.0 s, AQ = 1.36 s, 30°, P1 = 7.50 µs; 125 MHz: ns = 3072, D1 = 2.0 s, AQ = 1.10 s, 30°, P1 = 7.50 µs).
Full assignment of synthesized compounds was aided by 2D NMR analysis (*i.e.* COSY, HSQC and HMBC).

Size Exclusion Chromatography (SEC). Oligomers were characterized on a Waters SEC system equipped with a Waters 1515 isocratic pump, Waters 2410 refractive index detector (24 °C), Waters 717plus autosampler and a Waters 2487 dual λ absorbance UV detector and column oven. For separation, a three-column setup was used with one SDV 3 µm, 8×50 mm precolumn and two SDV 3 µm, 1000 Å, 8×300 mm columns supplied by PSS, Germany. Tetrahydrofuran (THF) stabilized with butylated hydroxytoluene (BHT, HPLC-SEC grade) supplied by Biosolve was used at a flow rate 1.0 mL min^{-1}. Calibration was carried out by three injections of a mixture of narrow polystyrene standards ranging from 162 to 38640 Da.

Matrix-Assisted Laser Desorption/Ionization Tandem Mass Spectrometry (MALDI-MS/MS). For the MALDI measurements a stock solution of the matrix, trans-2-[3-(4-tert-butylphenyl)-2-methyl-2-propenylidene]malonitrile (DCTB, 30 mg/ml) was prepared and the samples where solubilised in either tetrahydrofuran or acetonitrile (10 mg/ml). 45 µl of the matrix solution and 15 µl of the sample solution where mixed and subsequently spotted on the MALDI plate. The spots were dried at room temperature and loaded into an Applied Biosystems Sciex 4800+ MALDI-TOF/TOF analyser, controlled by 4000 Series Explorer software (Applied Biosystems, Germany). The instrument was operated in linear positive ion mode. Fragmentation (MS/MS) was performed in positive ion mode at 1 kV using air as collision gas with the 'metastable suppressor' and 'optimised precursor ion' options turned on.

High Resolution Mass Spectroscopy (HRMS). HRMS spectra were collected using an Agilent 6220 accurate-mass time-of-flight (TOF) analyzer equipped with a multimode ionization (MMI) source.

Infrared (IR). Measurements were recorded on a Perkin Elmer FTIR SPECTRUM 1000 spectrometer with Attenuated Total Reflection (ATR) with a PIKE Miracle ATR unit in a frequency range from 4000 to 600 cm^{-1}.

Thin layer chromatography (TLC). Experiments were performed on silica gel coated aluminum foil (silica gel 60 F_{254}, Sigma-Aldrich) or glass plates (0.25 mm, Macherey-Nagel). Compounds were visualized by staining with phosphomolybdic acid (PMA) upon heating.

2. Materials

Acetic acid (> 95.5 %, Sigma-Aldrich); acetophenone (99 %, Alfa Aesar); acrolein (90 %, hydroquinone inhibitor, Sigma-Aldrich); 1-adamantyl isocyanide (**L3d**, 97 %, Tokyo Chemical Industry - Europe); aminocaproic acid (≥ 99 %, Acros Organics); 4'-bromoacetophenone (98 %, Alfa Aesar); 4-bromophenylhydrazine hydrochloride (98 %, Fluorochem); butyl acetate (anhydrous, ≥ 99 %, Sigma-Aldrich); *tert*-butyl isocyanide (**L3b**, > 98 %, Tokyo Chemical Industry - Europe); 4-chlorophenylhydrazine hydrochloride (94 %, Fluorochem); concentrated aqueous hydrochloric acid (36 wt%, Chem-Lab); cyclohexyl isocyanide (**L3a**, > 98 %, Tokyo Chemical Industry - Europe); deuterated chloroform-*d* ($CDCl_3$, 99.8 %, Euriso-top); deuterated dimethylsulfoxide-d_6 (DMSO-d_6, 99.8 %, Euriso-top); 1-(3-dimethylaminopropyl)-3-ethylcarbodiimide hydrochloride (EDC.HCl, > 98 %, Tokyo Chemical Industry – Europe); 4-(dimethylamino)pyridine (DMAP, 99 %, Acros Organics); 2,3-dimethylbut-2-ene (≥ 96 %, Tokyo Chemical Industry – Europe); diphenyl carbonate (99 %, Acros Organics); ethyl carbazate (97 %, Tokyo Chemical Industry – Europe); ethyl 4-isocyanatobenzoate (97 %, Sigma-Aldrich); hydrochloric acid solution in 1,4-dioxane (4 N, Tokyo Chemical Industry - Europe); hydroquinone (99.5 %, Acros Organics); magnesium sulfate monohydrate (≥ 99 %, Roth); morpholine (≥ 99 %, Tokyo Chemical Industry - Europe); 1-pentyl isocyanide (**L3c**, 97 %, Sigma-Aldrich); 2-phenyl-1*H*-indole (**I2a**, > 98 %, Tokyo Chemical Industry - Europe); phosphomolybdic acid hydrate (99 %, Sigma-Aldrich); polyphosphoric acid (PPA, 115 % H_3PO_4, Honeywell); potassium carbonate (≥ 99 %, Roth); potassium hydroxide (≥ 99 %, Roth); silica (60 Å, ≥ 99.5 %, ROCC); sodium bicarbonate (99 %, Roth); sodium chloride (≥ 99 %, Roth); sorbic alcohol (*E,E*-2,4-hexadien-1-ol, HDEO, > 97 %, Sigma-Aldrich and 98 %, Alfa Aesar); stearic acid (98 %, Tokyo Chemical Industry - Europe); trichloroisocyanuric acid (TCICA, 99 %, Acros Organics); triethylamine (99 %, anhydrous, Fischer Scientific) and trifluoroacetic acid (TFA, peptide grade, Iris Biotech GmbH) were used as received from their supplier. All solvents (HPLC grade) were used without further purification.

3. Synthetic procedures

3.1 Synthesis of triazolinedione carboxylic acids L1a-b

*3.1.1 Synthesis of 6-(3,5-dioxo-3,5-dihydro-4H-1,2,4-triazol-4-yl)hexanoic acid (**L1a**)*

(i) Synthesis of 1-ethyl 2-phenyl hydrazine-1,2-dicarboxylate[1]

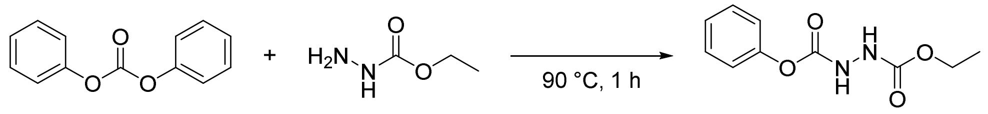

A mixture of diphenyl carbonate (60.0 g, 0.28 mol, 1.0 equiv.) and ethyl carbazate (58.4 g, 0.56 mol, 2.0 equiv.) was heated in bulk at 90 °C and stirred for 1 hour. The resulting mixture was poured into water (1.5 L), resulting in a suspension that was stirred vigorously for several hours until a white solid formed. The mixture was filtered, and the precipitate was collected, giving 1-ethyl 2-phenyl hydrazine-1,2-dicarboxylate as a white residue after overnight drying *in vacuo* at 40 °C. Yield = 64 % (40.2 g). **^{1}H-NMR (400 MHz, DMSO-d_6, see Figure S21):** δ (ppm) = 9.67 (s, 1H, Ar-O-C(O)-N*H*, [1]), 9.24 (s, 1H, CH2-O-C(O)-N*H*, [2]), 7.46-7.35 (m, 2H, Ar*H*, [4]), 7.30-7.21 (m, 1H, Ar*H*, [5]), 7.14-7.08 (m, 2H, Ar*H*, [6]), 4.07 (q, 2H, J = 7.1, CH_2, [7]), 1.19 (t, 3H, J = 7.1, CH_3, [8]). **^{13}C-NMR (100 MHz, DMSO-d_6):** δ(ppm) = 156.43 (C), 154.86 (C), 150.61 (C), 129.48 (CH), 125.39 (CH), 121.47 (CH), 60.70 (CH_2), 14.52 (CH_3). **HRMS (ESI) *m/z*:** $[M+H]^+$ for $[C_{10}H_{13}N_2O_4]^+$; *calculated*: 225.0870, *found*: 225.0872. **IR (ATR platinum diamond):** ν (cm^{-1}) = 3225 (N-H), 1742 (C=O), 1699 (C=O), 1519 (N-H),1489 (C=C), 1224 (C=C), 1189-1045 (C-O), 907-688 (=C-H).

(ii) Synthesis of 6-(2-(ethoxycarbonyl)hydrazine-1-carboxamido)hexanoic acid[1]

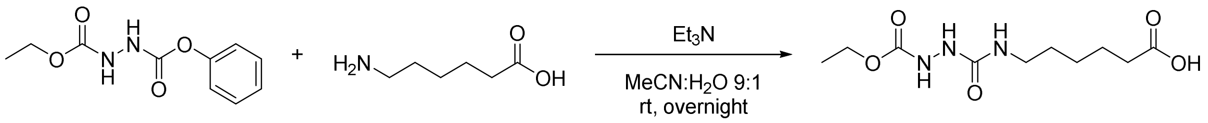

Aminocaproic acid (5.85 g, 44.6 mmol, 1.0 equiv.) and ethyl phenyl hydrazine dicarboxylate (10.0 g, 44.6 mmol, 1.0 equiv.) were dissolved in 150 mL of a 9:1 mixture of acetonitrile:water. Triethylamine (12.4 mL, 89.2 mmol, 2.0 equiv.) was added and the reaction was stirred for over 24 hours at room temperature. The acetonitrile was then removed *in vacuo* and the residual water phase was diluted further with water (400 mL) and extracted with ethyl acetate (3 x 100 mL) to remove the phenol by-product from the reaction. The aqueous phase was then acidified to pH 1 with concentrated aqueous hydrochloric acid (36 wt%) before removal of the water *in vacuo*. The resulting white residue was suspended in a minimal amount of water and stirred vigorously overnight to remove by-products. The resulting suspension was filtered off and the precipitate was dried overnight under vacuum at 40 °C to give the hexanoic acid semicarbazide as a white powder. Yield = 60 % (7.0 g).

^{1}H-NMR (400 MHz, DMSO-d_6, see Figure S22): δ(ppm) = 11.96 (s, 1H, COO*H*, [1]), 8.70 + 8.36 (s, 1H, CH_2-O-C(O)-N*H*, [2]), 7.61 (s, 1H, C(O)-NH-N*H*, [3]), 6.29 (s, 1H, N*H*-CH_2, [4]), 4.02 (q, 2H, *J* = 7.1, O-C*H*$_2$, [5]), 2.97 (q, 2H, *J* = 6.8, N-C*H*$_2$, [6]), 2.18 (t, 2H, *J* = 7.4, C*H*$_2$-COOH, [7]), 1.48 (quin, 2H, *J* = 7.5, N-CH_2-C*H*$_2$, [8]), 1.36 (m, 2H, C*H*$_2$-CH_2-COOH, [9]), 1.24 (m, 2H, N-$(CH_2)_2$-C*H*$_2$, [10]), 1.17 (t, 3H, *J* = 7.1, C*H*$_3$, [11]). **^{13}C-NMR (100 MHz, DMSO-d_6):** δ(ppm) = 174.44 (C), 158.22 (C), 156.89 (C), 60.30 (CH_2), 38.96 (CH_2), 33.64 (CH_2), 29.58 (CH_2), 25.81 (CH_2), 24.25 (CH_2), 14.54 (CH_3). **HRMS (ESI) *m/z*:** $[M+H]^+$ for $[C_{10}H_{20}N_3O_5]^+$; *calculated*: 262.1403, *found*: 262.1396. **IR (ATR platinum diamond):** ν (cm^{-1}) = 3283 (N-H), 2939 (O-H), 1728 (C=O), 1711 (C=O), 1662 (C=O), 1560-1531 (N-H), 1474 (CH_2), 1365 (CH_3), 1272-1056 (C-O),738 ($(CH_2)_n$).

(iii) Synthesis of 6-(3,5-dioxo-1,2,4-triazolidin-4-yl)hexanoic acid[1]

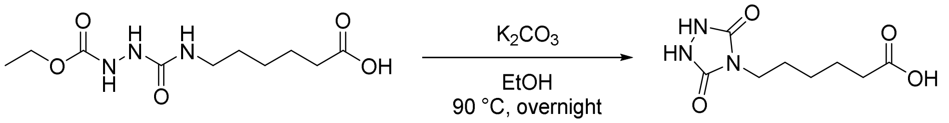

A mixture of 6-(2-(ethoxycarbonyl)hydrazine-1-carboxamido)hexanoic acid (6.05 g, 23.0 mmol, 1.0 equiv.) and potassium carbonate (12.7 g, 92.0 mmol, 4.0 equiv.) in 100 mL ethanol was stirred at reflux overnight (90 °C). The resulting mixture was cooled to room temperature, filtered and the filtrate was evaporated *in vacuo* to complete dryness. The residue was solubilised in a minimal amount of 1,4-dioxane and acidified to pH = 1 with hydrochloric acid solution in 1,4-dioxane (4 N). The formed precipitate was filtered, and the filtrate was removed *in vacuo* to give the hexanoic acid-based urazole as a white solid. Yield = 67 % (3.30 g).

^{1}H-NMR (400 MHz, DMSO-d_6, see Figure S23): δ(ppm) = 11.99 (s, 1H, COO*H*, [1]), 10.02 (s, 2H, N*H*, [2]), 3.35 (m, 2H, N-C*H*$_2$, [3]), 2.18 (t, 2H, *J* = 7.3, C*H*$_2$-COOH, [4]), 1.50 (m, 4H, N-CH_2-C*H*$_2$-CH_2-C*H*$_2$, [5]), 1.28-1.18 (m, 2H, N-CH_2-CH_2-C*H*$_2$, [6]). **^{13}C-NMR (100 MHz, DMSO-d_6):** δ (ppm) = 174.38 (C), 155.06 (C), 37.75 (CH_2), 33.49 (CH_2), 27.27 (CH_2), 25.59 (CH_2), 24.02 (CH_2). **HRMS (ESI) *m/z*:** $[M+H]^+$ for $[C_8H_{14}N_3O_4]^+$; *calculated*: 216.0984, *found*: 216.0981. **IR (ATR platinum diamond):** ν (cm^{-1}) = 3166 (N-H), 2931 (O-H), 1668 (C=O), 1474 (N-H), 1415 (CH_2), 1348 (C-N), 1225-1030 (C-O), 735 ($(CH_2)_n$).

*(iv) Synthesis of 6-(3,5-dioxo-4H-1,2,4-triazol-4-yl)hexanoic acid (**L1a**)*

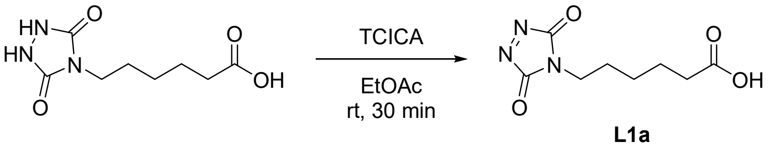

A mixture of the 6-(3,5-dioxo-1,2,4-triazolidin-4-yl)hexanoic acid (1.50 g, 6.94 mmol, 1.0 equiv.) and trichloroisocyanuric acid (TCICA, 0.54 g, 2.31 mmol, 1/3 equiv.) in 100 mL anhydrous ethyl acetate

was placed under inert atmosphere and stirred vigorously for 30 minutes at room temperature. The resulting mixture was filtered and concentrated *in vacuo* (keeping the temperature below 35 °C) to give the hexanoic acid-based triazolinedione **L1a** as a vivid pink powder. Yield = 100 % (1.49 g).

^{1}H-NMR (400 MHz, DMSO-d_6, see Figure S24): δ(ppm) = 12.01 (s, 1H, COO*H*, [1]), 3.46 (t, 2H, *J* = 7.1, N-CH_2, [2]), 2.18 (t, 2H, *J* = 7.3, CH_2-COOH, [3]), 1.64-1.43 (m, 4H, N-CH_2-CH_2-CH_2-CH_2, [4]), 1.34-1.22 (m, 2H, N-$(CH_2)_2$-CH_2, [5]). **^{13}C-NMR (100 MHz, DMSO-d_6):** δ(ppm) = 174.34 (C), 160.15 (C), 40.50 (CH_2), 33.42 (CH_2), 26.39 (CH_2), 25.38 (CH_2), 23.89 (CH_2). **HRMS (ESI) *m/z*:** $[M+H]^+$ for $[C_8H_{12}N_3O_4]^+$; *calculated*: 214.0822, *found*: 214.1879; $[M+Na]^+$ for $[C_8H_{11}N_3O_4Na]^+$; *calculated*: 236.0642, *found*: 236.1720. **IR (ATR platinum diamond):** ν (cm^{-1}) = 2934 (O-H), 1742 (C=O), 1698 (C=O), 1393-1336 (C-N), 1270-1137 (C-O), 728 ((C-H).

3.1.2 Synthesis of 4-(3,5-dioxo-3,5-dihydro-4H-1,2,4-triazol-4-yl)benzoic acid (L1b)

(i) Synthesis of ethyl 2-((4-(ethoxycarbonyl)phenyl)carbamoyl)hydrazine-1-carboxylate

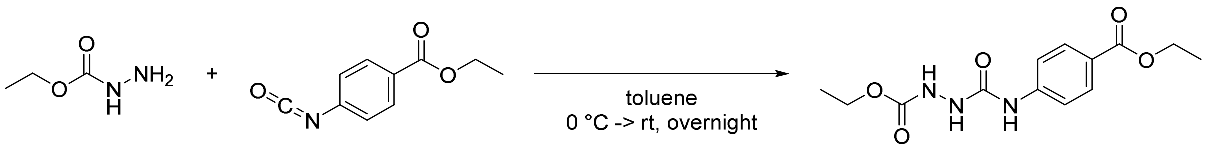

A solution of ethyl carbazate (5.45 g, 0.052 mol, 1.0 equiv.) in 100 mL toluene was placed under inert atmosphere and cooled in a water-ice bath at 0 °C. To this, a solution of ethyl 4-isocyanatobenzoate (10.0 g, 0.052 mol, 1.0 equiv.) in 100 mL toluene was added dropwise by means of an addition funnel, after which the reaction mixture was allowed to warm up to room temperature and stirred overnight. The resulting cloudy mixture was filtered off, washed with toluene (50 mL) and the residue was dried overnight in a vacuum oven at 40 °C to give ethyl 2-((4-(ethoxycarbonyl)phenyl)carbamoyl)hydrazine-1-carboxylate as a white powder. Yield = 96 % (14.8 g).

^{1}H-NMR (400 MHz, DMSO-d_6, see Figure S25): δ(ppm) = 9.18 (s, 1H, O-C(O)-N*H*, [1]), 8.98 + 8.60 (s, 1H, O-C(O)-NH-N*H*, [2]), 8.20 (s, 1H, NH, Ar-N*H*, [3]), 7.85 (m, 2H, Ar*H*, [4]), 7.61 (m, 2H, Ar*H*, [5]), 4.27 (q, *J* = 14.2, 7.1 Hz, 2H, Ar-C(O)O-CH_2, [6]), 4.06 (q, *J* = 14.3, 7.1 Hz, 2H, N-C(O)O-CH_2, [7]), 1.30 (t, *J* = 7.2 Hz, 3H, Ar-C(O)O-CH_2-CH_3, [8]), 1.20 (t, *J* = 6.9 Hz, 3H, N-C(O)O-CH_2-CH_3, [9]). **^{13}C-NMR (100 MHz, DMSO-d_6):** δ (ppm) = 165.44 (C), 156.88 (C), 155.29 (C), 144.38 (C), 130.18 (CH), 122.74 (C), 117.54 (CH), 60.58 (CH_2), 60.25 (CH_2), 14.53 (CH_3), 14.23 (CH_3).

(ii) Synthesis of 4-(3,5-dioxo-1,2,4-triazolidin-4-yl)benzoic acid

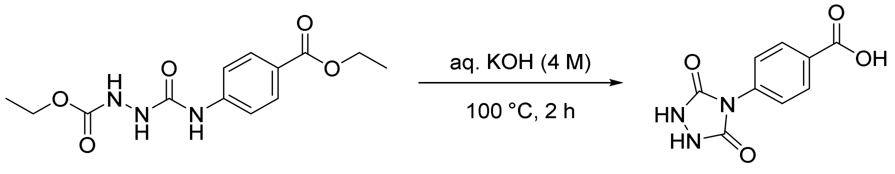

A suspension of ethyl 2-((4-(ethoxycarbonyl)phenyl)carbamoyl)hydrazine-1-carboxylate (5.0 g, 16.9 mmol, 1.0 equiv.) in 15.7 mL of a 4 M aqueous potassium hydroxide solution was placed under inert atmosphere and stirred at reflux (100 °C) for 2 hours. The resulting faint yellow clear solution was cooled to room temperature and subsequently placed in an ice-water bath at 0 °C before acidifying to pH 1 upon the addition of a 1 M aqueous hydrochloric acid solution. The formed precipitate was filtered off, washed with water (10 mL) and dried in a vacuum oven overnight at 40 °C to give 4-(3,5-dioxo-1,2,4-triazolidin-4-yl)benzoic acid as a white powder. Yield = 99 % (3.7 g).

^{1}H-NMR (400 MHz, DMSO-*d_6*, see Figure S26): δ (ppm) = 13.06 (s, 1H, COO*H*, [1]), 10.66 (s, 2H, N*H*, [2]), 8.04 (m, 2H, Ar*H*, [3]), 7.66 (m, 2H, Ar*H*, [4]). **^{13}C-NMR (100 MHz, DMSO-*d_6*):** δ (ppm) = 166.73 (C), 152.78 (C), 135.97 (C), 129.85 (CH), 129.43 (C), 125.22 (CH). **LC-ESI-MS *m/z*:** $[M-H]^-$ for $[C_9H_6N_3O_4]^-$; *calculated*: 220.0364, *found*: 220.10.

*(iii) Synthesis of 4-(3,5-dioxo-3,5-dihydro-4H-1,2,4-triazol-4-yl)benzoic acid (**L1b**)*

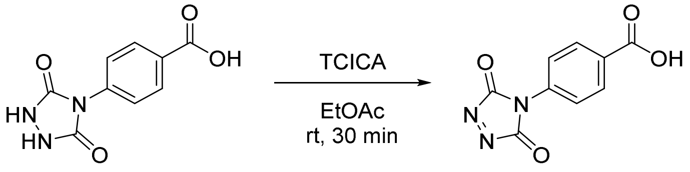

L1b

A suspension of 4-(3,5-dioxo-1,2,4-triazolidin-4-yl)benzoic acid (1.00 g, 4.52 mmol, 1.0 equiv.) and trichloroisocyanuric acid (TCICA, 0.35 g, 1.51 mmol, $^1/_3$ equiv.) in 30 mL ethyl acetate was placed under inert atmosphere and stirred at room temperature for 30 minutes whilst shielded from ambient light. The resulting pink heterogenous mixture was filtered, washed with ethyl acetate (10 mL) and the filtrate was evaporated *in vacuo* to dryness (keeping the temperature below 35 °C) to give 4-(3,5-dioxo-3,5-dihydro-4*H*-1,2,4-triazol-4-yl)benzoic acid (**L1b**) as a pink powder. Yield = 83 % (0.82 g).

^{1}H-NMR (400 MHz, DMSO-*d_6*, see Figure S27): δ (ppm) = 13.16 (s, 1H, COO*H*, [1]), 8.15 (m, 2H, Ar*H*, [2]), 7.60 (m, 2H, Ar*H*, [3]). **^{13}C-NMR (100 MHz, DMSO-*d_6*):** δ (ppm) = 166.43 (C), 158.31 (C), 133.92 (C), 131.09 (C), 130.61 (CH), 124.83 (CH). **LC-ESI-MS *m/z*:** [M+**S1**+H]$^+$ for $[C_{33}H_{50}N_3O_6]^+$; *calculated*: 584.3694, *found*: 584.30. Submitted as the Diels-Alder adduct upon reaction of **L1b** with (2*E*,4*E*)-hexa-2,4-dien-1-yl stearate **S1**.

3.2 Synthesis of substituted 2-phenyl-1*H*-indole aldehydes L2a-d

*3.2.1 Synthesis of substituted 2-phenyl-1H-indole precursors (**l2b-d**)*

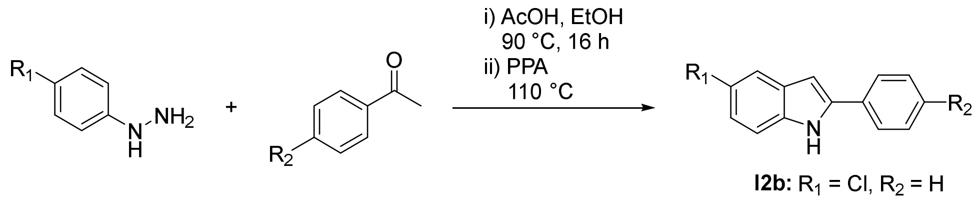

*(i) Synthesis of 5-chloro-2-phenyl-1H-indole (**l2b**)*

A mixture of 4-chlorophenylhydrazine hydrochloride (5.94 g, 33.2 mmol, 1.1 equiv.) and acetophenone (3.51 mL, 30.2 mmol, 1.0 equiv.) in 115 mL of a 9:1 v% ethanol:acetic acid solution was placed under inert atmosphere and stirred under reflux (90 °C) overnight. The resulting mixture was cooled to room temperature and the solvent was removed *in vacuo* to give a yellow-brown residue. To this, polyphosphoric acid (PPA) (ca. 50 g) was added and the resulting mixture was heated to 110 °C for 4 hours. The dark brown mixture was poured into ice-water (400 mL) whilst hot and stirred vigorously overnight at room temperature. 200 mL of ethyl acetate was added to the resulting heterogenous mixture and the organic phase was separated. The aqueous phase was extracted with another 2 x 200 mL ethyl acetate and the collected organic phases were washed with 2 x 100 mL brine, followed by drying over magnesium sulfate and solvent removal *in vacuo*. The resulting brown oil was purified by column chromatography (silica, hexane:ethyl acetate 4:1, R_F = 0.31) to give 5-chloro-2-phenyl-1*H*-indole **l2b** as a yellow powder. Yield = 71 % (4.85 g).

^{1}H-NMR (400 MHz, DMSO-*d₆*, see Figure S28): δ(ppm) = 11.73 (s, 1H, N*H*, [1]), 7.86 (m, 2H, Ar*H*, [2]), 7.57 (d, *J* = 2.2 Hz, 1H, Ar*H*, [3]), 7.47 (m, 2H, Ar*H*, [4]), 7.40 (d, *J* = 8.5 Hz, 1H, Ar*H*, [5]), 7.34 (m, 1H, Ar*H*, [6]), 7.09 (dd, *J* = 8.5, 2.1 Hz, 1H, Ar*H*, [7]), 6.93 (dd, *J* = 2.1, 0.8 Hz, 1H, Ar*H*, [8]). **^{13}C-NMR (100 MHz, DMSO-d_6, see Figure S28):** δ (ppm) = 139.31 (C, [1]), 135.54 (C, [2]), 131.66 (CH, [3]), 129.75 (C, [4]), 128.95 (CH, [5]), 127.84 (CH, [6]), 125.15 (CH, [7]), 123.86 (C, [8]), 121.42 (CH, [9]), 119.05 (CH, [10]), 112.74 (CH, [11]), 98.35 (CH, [12]). **HRMS (ESI) *m/z*:** $[M-H]^-$ for $[C_{14}H_9ClN]^-$, *calculated*: 226.0429, *found*: 226.0435.

*(ii) Synthesis of 5-bromo-2-phenyl-1H-indole (**l2c**)*

A mixture of 4-bromophenylhydrazine hydrochloride (12.6 g, 56.6 mmol, 1.1 equiv.) and acetophenone (6.00 mL, 51.4 mmol, 1.0 equiv.) in 200 mL of a 9:1 v% ethanol:acetic acid solution was placed under inert atmosphere and stirred under reflux (90 °C) overnight. The resulting mixture was cooled to room temperature and the solvent was removed *in vacuo* to give a yellow-brown residue. To this,

polyphosphoric acid (PPA) (ca. 100 g) was added and the resulting mixture was heated to 110 °C for 2 hours. The dark brown mixture was poured into ice-water (800 mL) whilst hot and stirred vigorously overnight at room temperature. 400 mL of ethyl acetate was added to the resulting heterogenous mixture and the organic phase was separated. The aqueous phase was extracted with another 2 x 400 mL ethyl acetate and the collected organic phases were washed with 2 x 200 mL brine, followed by drying over magnesium sulfate and solvent removal *in vacuo*. The resulting dark brown oil was purified by column chromatography (silica, hexane:ethyl acetate 4:1, R_F = 0.35) to give 5-bromo-2-phenyl-1*H*-indole **l2c** as a pale brown powder. Yield = 47 % (6.52 g).

^{1}H-NMR (400 MHz, DMSO-*d₆*, see Figure S29): δ(ppm) = 11.74 (s, 1H, N*H*, ^{1}), 7.86 (m, 2H, Ar*H*, ^{2}), 7.71 (d, *J* = 2.0 Hz, 1H, Ar*H*, ^{3}), 7.47 (m, 2H, Ar*H*, ^{4}), 7.38-7.31 (m, 2H, Ar*H*, $^{5+6}$), 7.20 (dd, *J* = 8.5, 2.0 Hz, 1H, Ar*H*, ^{7}), 6.89 (dd, *J* = 2.1, 0.7 Hz, 1H, Ar*H*, ^{8}). **^{13}C-NMR (APT, 100 MHz, DMSO-d_6, see Figure S29):** δ (ppm) = 139.13 (C, ^{1}), 135.75 (C, ^{2}), 131.61 (CH, ^{3}), 130.48 (C, ^{4}), 128.95 (CH, ^{5}), 127.85 (CH, ^{6}), 125.16 (CH, ^{7}), 123.94 (CH, ^{8}), 122.09 (CH, ^{9}), 113.21 (CH, ^{10}), 111.83 (C, ^{11}), 98.23 (CH, ^{12}). **HRMS (ESI) *m/z*:** $[M-H]^-$ for $[C_{14}H_9BrN]^-$; *calculated*: 269.9924, *found*: 269.9930.

*(iii) Synthesis of 5-bromo-2-(4-bromophenyl)-1H-indole (**l2d**)*

A mixture of 4-bromophenylhydrazine hydrochloride (5.97 g, 26.7 mmol, 1.1 equiv.) and 4’-bromoacetophenone (4.84 mL, 24.3 mmol, 1.0 equiv.) in 120 mL of a 9:1 v% ethanol:acetic acid solution was placed under inert atmosphere and stirred under reflux (90 °C) overnight. The resulting mixture was cooled to room temperature and the solvent was removed *in vacuo* to give a yellow-greenish residue. To this, polyphosphoric acid (PPA) (ca. 50 g) was added and the resulting mixture was heated to 110 °C for 2 hours. The dark brown mixture was poured into ice-water (800 mL) whilst hot and stirred vigorously overnight at room temperature. 250 mL of ethyl acetate was added to the resulting grey heterogenous mixture and the organic phase was separated. The aqueous phase was extracted with another 2 x 250 mL ethyl acetate and the collected organic phases were washed with 2 x 125 mL of brine, followed by drying over magnesium sulfate and solvent removal *in vacuo*. The resulting brown residue was suspended in 50 mL of water and heated to reflux, followed by the slow addition of 210 mL of ethanol until completely solubilized. Upon cooling to room temperature, 5-bromo-2-(4-bromophenyl)-1*H*-indole **l2d** was collected via filtration as a yellow powder. Yield = 52 % (4.40 g).

^{1}H-NMR (400 MHz, DMSO-d_6, see Figure S30): δ(ppm) = 11.80 (s, 1H, N*H*, ^{1}), 7.81 (m, 2H, Ar*H*, ^{2}), 7.72 (d, *J* = 2.0 Hz, 1H, Ar*H*, ^{3}), 7.67 (m, 2H, Ar*H*, ^{4}), 7.36 (d, *J* = 8.5 Hz, 1H, Ar*H*, ^{5}), 7.22 (dd, *J* = 8.5, 2.0 Hz, 1H, Ar*H*, ^{6}), 6.93 (dd, *J* = 2.1, 0.7 Hz, 1H, Ar*H*, ^{7}). **^{13}C-NMR (APT, 100 MHz, DMSO-d_6, see Figure S30):** δ(ppm) = 137.92 (C, ^{1}), 135.88 (C, ^{2}), 131.90 (CH, ^{3}), 130.90 (C, ^{4}), 130.39 (C, ^{5}), 127.11 (CH, ^{6}), 124.33 (CH, ^{7}), 122.27 (CH, ^{8}), 120.87 (C, ^{9}), 113.32 (CH, ^{10}), 112.01 (C, ^{11}), 98.91 (CH, ^{12}). **HRMS (ESI) *m/z*:** $[M-H]^-$ for $[C_{14}H_8Br_2N]^-$; *calculated*: 347.9029, *found*: 347.9043.

3.2.2 *Synthesis of substituted 2-phenyl-1H-indole aldehydes* ***L2a-d***

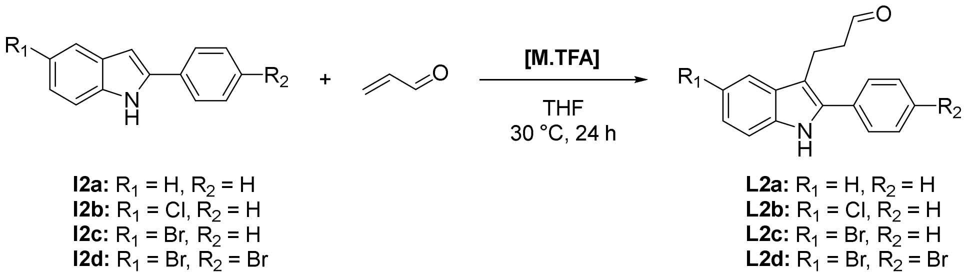

(i) Synthesis of morpholinium trifluoroacetate salt ***[M.TFA]***[2]

A solution of morpholine (1.72 mL, 20.0 mmol, 1.0 equiv.) in 40 mL diethyl ether was cooled in an ice-water bath at 0 °C. To this, trifluoroacetic acid (TFA, 1.68 mL, 22.0 mmol, 1.1 equiv.) solubilized in 20 mL diethyl ether was added dropwise. After stirring for 1 hour, the reaction mixture was allowed to warm up to room temperature and the resulting white precipitate was filtered, washed with 2 x 10 mL diethyl ether and dried overnight under vacuum at 40°C to give morpholinium trifluoroacetate salt [**M.TFA**] as a white powder. Yield = 95 % (3.83 g).

^{1}H-NMR (400 MHz, DMSO-*d*$_6$): δ= 9.02 (s, 2H, N*H*$_2$), 3.83-3.69 (m, 2H, O-C*H*$_2$), 3.19-3.01 (m, 2H, N-C*H*$_2$). **^{13}C-NMR (APT, 100 MHz, DMSO-*d*$_6$):** δ(ppm) = 63.29 (C-O), 42.77 (C-N). **HRMS (ESI) *m/z*:** $[M+H]^+$ for $[C_4H_{10}NO]^+$; *calculated*: 88.0757, *found*: 88.0759; $[M-H]^-$ for $[C_2F_3O_2]^-$; *calculated*: 112.9856, *found*: 112.9860. **IR (ATR platinum diamond):** *v* [cm^{-1}] = 3340 (N-H), 3100-2500 (O-H), 1704 (C=O) 1439 (N-H), 1310 (C-O), 1172 (C-N), 1104 (C-F).

(ii) Synthesis of 3-(2-phenyl-1H-indol-3-yl)propanal ***L2a***

To a mixture of 2-phenyl-1*H*-indole (**I2a**, 6.00 g, 31.1 mmol, 1.0 equiv.) and morpholinium trifluoroacetate salt ([**M.TFA**], 1.26 g, 6.2 mmol, 0.2 equiv.) in 200 mL tetrahydrofuran, acrolein (6.20 mL, 93.2 mmol, 3.0 equiv.) was added. The resulting solution was placed under an inert atmosphere and stirred at 30 °C for 24 hours, until complete consumption of the indole substrate (monitored via thin layer chromatography). The resulting mixture was cooled to room temperature and the solvent was evaporated *in vacuo* to give an orange oil. The residue was dissolved in ethyl acetate (300 mL) and extracted with H_2O (30 mL), saturated aqueous sodium bicarbonate (2 x 30 mL) and brine (2 x 30 mL). The combined organic phases were dried over magnesium sulfate and evaporated *in vacuo* to dryness. A small amount of hydroquinone was added as stabilizer prior to solvent removal whilst the temperature of the water bath was kept below 35 °C. The resulting orange oil was purified by column chromatography (silica, hexane:ethyl acetate 4:1, R_F = 0.27) to give 3-(2-phenyl-1*H*-indol-3-yl)propanal **L2a** as an off-white powder. Yield = 61 % (3.66 g).

^{1}H-NMR (400 MHz, DMSO-d_6, see Figure S31): δ = 11.19 (s, 1H, N*H*, [1]), 9.72 (t, *J* = 1.5 Hz, 1H, C*H*O, [2]), 7.45-7.67 (m, 5H, Ar*H*, [3+4+5]), 7.31-7.45 (m, 2H, Ar*H*, [6+7]), 7.12 (dd, *J* = 8.1, 7.0, 1.3 Hz, 1H, Ar*H*, [8]), 7.02 (ddd, *J* = 7.9, 7.0, 1.1 Hz, 1H, Ar*H*, [9]), 3.13 (m, 2H, C*H*$_2$-CH$_2$-CHO, [10]), 2.80 (m, 2H, C*H*$_2$-CHO, [11]). **^{13}C-NMR (APT, 100 MHz, DMSO-d_6, see Figure S31):** δ(ppm) = 203.06 (CH, [1]), 135.99 (C, [2]), 134.18 (C, [3]), 132.82 (C, [4]), 128.76 (CH, [5]), 128.24 (C, [6]), 127.83 (CH, [7]), 127.38 (CH, [8]), 121.57 (CH, [9]), 118.74 (CH, [10]), 118.53 (CH, [11]), 111.19 (CH, [12]), 110.12 (C, [13]), 44.11 (CH$_2$, [14]), 16.96 (CH$_2$, [15]). **HRMS (ESI) *m/z*:** [M+H]$^+$ for [C$_{17}$H$_{16}$NO]$^+$; *calculated*: 250.1226, *found*: 250.1215. IR (ATR platinum diamond): *v* [cm^{-1}] = 3329 (N-H), 3058 ($_{Ar}$C-H), 2824 & 2725 (C-H), 1703 (C=O), 1602 (N-H), 1486 (C-H), 1448 (C=C) 1071 (C-N).

(iii) Synthesis of 3-(5-chloro-2-phenyl-1H-indol-3-yl)propanal ***L2b***

To a mixture of 5-chloro-2-phenyl-1*H*-indole (**I2b**, 4.70 g, 20.6 mmol, 1.0 equiv.) and morpholinium trifluoroacetate salt ([**M.TFA**], 0.83 g, 4.13 mmol, 0.2 equiv.) in 150 ml tetrahydrofuran, acrolein (4.14 mL, 61.9 mmol, 3.0 equiv.) was added. The resulting solution was placed under an inert atmosphere and stirred at 30 °C for 24 hours, until complete consumption of the indole substrate (monitored via thin layer chromatography). The resulting mixture was cooled to room temperature and the solvent was evaporated *in vacuo* to give a brown oil. The residue was purified by column chromatography (silica, hexane:ethyl acetate 4:1, R$_F$ = 0.24) to give 3-(5-chlorophenyl-1*H*-indol-3-yl)propanal **L2b** as a yellow oil. A small amount of hydroquinone was added as stabilizer prior to solvent removal whilst the temperature of the water bath was kept below 35 °C. Yield = 56 % (3.28 g). **^{1}H-NMR (400 MHz, DMSO-d_6, see Figure S32):** δ(ppm) = 11.41 (s, 1H, N*H*, [1]), 9.71 (t, *J* =1.4 Hz, 1H, C*H*O, [2]), 7.65 (d, *J* = 2.1 Hz, 1H, Ar*H*, [3]), 7.61 (m, 2H, Ar*H*, [4]), 7.52 (m, 2H, Ar*H*, [5]), 7.41 (m, 1H, Ar*H*, [6]), 7.36 (dd, *J* = 8.5, 0.4 Hz, 1H, Ar*H*, [7]), 7.10 (dd, *J* = 8.6, 2.0 Hz, 1H, Ar*H*, [8]), 3.09 (m, 2H, C*H*$_2$-CH$_2$-CHO, [9]), 2.78 (m, 2H, C*H*$_2$-CHO, [10]). **^{13}C-NMR (APT, 100 MHz, DMSO-d_6, see Figure S32):** δ (ppm) = 202.95 (CH, [1]), 135.95 (C, [2]), 134.38 (C, [3]), 132.29 (C, [4]), 129.39 (C, [5]), 128.82 (CH, [6]), 127.90 (CH, [7]), 127.76 (CH, [8]), 123.38 (C, [9]), 121.46 (CH, [10]), 117.83 (CH, [11]), 112.67 (CH, [12]), 110.10 (C, [13]), 43.94 (CH$_2$, [14]), 16.73 (CH$_2$, [15]). **HRMS (ESI) *m/z*:** [M-H]$^-$ for [C$_{17}$H$_{13}$ClNO]$^-$; *calculated*: 282.0691, *found*: 282.0701.

(iv) Synthesis of 3-(5-bromo-2-phenyl-1H-indol-3-yl)propanal ***L2c***

To a mixture of 5-bromo-2-phenyl-1*H*-indole (**I2c**, 5.00 g, 18.4 mmol, 1.0 equiv.) and morpholinium trifluoroacetate salt ([**M.TFA**], 0.74 g, 3.67 mmol, 0.2 equiv.) in 100 mL tetrahydrofuran, acrolein (3.68 mL, 55.1 mmol, 3.0 equiv.) was added. The resulting solution was placed under an inert atmosphere and stirred at 30 °C for 24 hours, until complete consumption of the indole substrate (monitored via thin layer chromatography). The resulting mixture was cooled to room temperature and

the solvent was evaporated *in vacuo*. The resulting brown oil was purified by column chromatography (silica, hexane:ethyl acetate 4:1, R_F = 0.20) to give 3-(5-bromophenyl-1*H*-indol-3-yl)propanal **L2c** as a pale yellow powder. A small amount of hydroquinone was added as stabilizer prior to solvent removal whilst the temperature of the water bath was kept below 35 °C. Yield = 36 % (2.17 g).

^{1}H-NMR (400 MHz, DMSO-*d*$_6$, see Figure S33): δ(ppm) = 11.42 (s, 1H, N*H*, ^{1}), 9.70 (t, *J* =1.3 Hz, 1H, C*H*O, ^{2}), 7.79 (d, *J* = 1.8 Hz, 1H, Ar*H*, ^{3}), 7.61 (m, 2H, Ar*H*, ^{4}), 7.52 (m, 2H, Ar*H*, ^{5}), 7.41 (m, 1H, Ar*H*, ^{6}), 7.32 (dd, *J* = 8.5, 0.4 Hz, 1H, Ar*H*, ^{7}), 7.21 (dd, *J* = 8.5, 2.0 Hz, 1H, Ar*H*, ^{8}), 3.08 (m, 2H, CH_2-CH_2-CHO, ^{9}), 2.78 (m, 2H, CH_2-CHO, ^{10}). **^{13}C-NMR (APT, 100 MHz, DMSO-*d*$_6$, see Figure S33):** δ (ppm) = 202.94 (CH, ^{1}), 135.76 (C, ^{2}), 134.61 (C, ^{3}), 132.24 (CH, ^{4}), 130.09 (C, ^{5}), 128.83 (CH, ^{6}), 127.92 (CH, ^{7}), 127.77 (CH, ^{8}), 124.00 (CH, ^{9}), 120.84 (CH, ^{10}), 113.14 (CH, ^{11}), 111.35 (C, ^{12}), 110.02 (C, ^{13}), 43.94 (CH_2, ^{14}), 16.72 (CH_2, ^{15}). **HRMS (ESI) *m/z*:** $[M+H]^+$ for $[C_{17}H_{15}BrNO]^+$; *calculated*: 328.0332, *found*: 328.0321.

(v) Synthesis of 3-(5-bromo-2-(4-bromophenyl)-1H-indol-3-yl)propanal ***L2d***

To a mixture of 5-bromo-2-(4-bromophenyl)-1*H*-indole (**l2d**, 4.00 g, 11.4 mmol, 1.0 equiv.) and morpholinium trifluoroacetate salt ([**M.TFA**], 0.46 g, 2.28 mmol, 0.2 equiv.) in 80 mL tetrahydrofuran, acrolein (2.29 mL, 34.2 mmol, 3.0 equiv.) was added. The resulting solution was placed under an inert atmosphere and stirred at 30 °C for 24 hours, until complete consumption of the indole substrate (monitored via thin layer chromatography). The resulting mixture was cooled to room temperature and the solvent was evaporated *in vacuo*. The resulting brown oil was purified by column chromatography (silica, hexane:ethyl acetate 4:1, R_F = 0.19) to give 3-(5-bromo-2-(4-bromophenyl)-1*H*-indol-3-yl)propanal **L2d** as a yellow powder. A small amount of hydroquinone was added as stabilizer prior to solvent removal whilst the temperature of the water bath was kept below 35 °C. Yield = 54 % (2.51 g).

^{1}H-NMR (400 MHz, DMSO-*d*$_6$, see Figure S34): δ(ppm) = 11.48 (s, 1H, N*H*, ^{1}), 9.69 (t, *J* =1.3 Hz, 1H, C*H*O, ^{2}), 7.80 (d, *J* = 2.0 Hz, 1H, Ar*H*, ^{3}), 7.71 (m, 2H, Ar*H*, ^{4}), 7.56 (m, 2H, Ar*H*, ^{5}), 7.32 (dd, *J* = 8.6, 0.3 Hz, 1H, Ar*H*, ^{6}), 7.23 (dd, *J* = 8.6, 1.9 Hz, 1H, Ar*H*, ^{7}), 3.13 (m, 2H, CH_2-CH_2-CHO, ^{8}), 2.80 (m, 2H, CH_2-CHO, ^{9}). **^{13}C-NMR (APT, 100 MHz, DMSO-*d*$_6$, see Figure S34):** δ(ppm) = 202.85 (CH, ^{1}), 134.69 (C, ^{2}), 134.46 (C, ^{3}), 131.77 (CH, ^{4}), 131.43 (C, ^{5}), 130.02 (C, ^{6}), 129.87 (CH, ^{7}), 124.33 (CH, ^{8}), 121.03 (C, ^{9}), 120.98 (CH, ^{10}), 113.22 (CH, ^{11}), 111.48 (C, ^{12}), 110.64 (C, ^{13}), 43.80 (CH_2, ^{14}), 16.64 (CH_2, ^{15}). **HRMS (ESI) *m/z*:** $[M-H]^-$ for $[C_{17}H_{12}Br_2NO]^-$; *calculated*: 403.9291, *found*: 403.9300.

3.3 Synthesis of macromolecular pin codes PC1 and PC2

3.3.1 Synthesis of (2E,4E)-hexa-2,4-dien-1-yl stearate (S1)

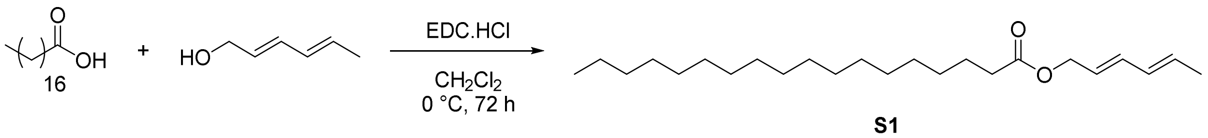

A mixture of stearic acid (5.69 g, 20.0 mmol, 1.0 equiv.), sorbic alcohol (2.36 g, 24.0 mmol, 1.2 equiv.) and 4-(dimethylamino)pyridine (DMAP, 0.50 g, 4.0 mmol, 0.2 equiv.) in 50 mL anhydrous dichloromethane was placed under inert atmosphere and cooled in a water-ice bath to 0 °C. To this, a suspension of 1-(3-dimethylaminopropyl)-3-ethylcarbodiimide hydrochloride (EDC.HCl, 4.60 g, 24.0 mmol, 1.2 equiv.) in 30 mL anhydrous dichloromethane was added dropwise to the cooled solution. The reaction mixture was stirred for 72 hours in a water bath, after which the resulting colorless solution was extracted with water (2 x 50 mL) and brine (1 x 50 mL). The combined organic phases were dried over magnesium sulfate, filtered and concentrated *in vacuo*. The residue was recrystallized from water (50 mL) upon slow addition of ethanol (250 mL) under reflux conditions to give (2*E*,4*E*)-hexa-2,4-dien-1-yl stearate (**S1**) as white needles after drying overnight in a vacuum oven at 40 °C. Yield = 75 % (5.49 g)

^{1}H-NMR (400 MHz, $CDCl_3$, see Figure S35): δ = 6.26 (m, 1H, CH_3-CH=CH-C*H*, [1]), 6.06 (m, 1H, CH_3-CH=C*H*, [2]), 5.77 (m, 1H, CH_3-C*H*, [3]), 5.63 (m, 1H, O-CH_2-C*H*, [4]), 4.58 (d, *J* = 6.5 Hz, 2H, O-CH_2, [5]), 2.31 (t, *J* = 7.5 Hz, 2H, C(O)-CH_2, [6]), 1.77 (d, *J* = 6.6 Hz, 3H, CH-CH_3, [7]), 1.63 (m, 2H, C(O)-CH_2-CH_2, [8]), 1.26 (m, 28H, CH_3-(CH_2)$_{14}$, [9]), 0.89 (t, *J* = 6.8 Hz, 3H, CH_2-CH_3, [10]). **^{13}C-NMR (APT, 100 MHz, $CDCl_3$, see Figure S35):** δ = 173.66 (C, [1]), 134.76 (CH, [2]), 131.16 (CH, [3]), 130.47 (CH, [4]), 123.87 (CH, [5]), 64.67 (CH_2, [6]), 34.38 (CH_2, [7]), 31.94 (CH_2, [8]), 29.69-29.14 (CH_2, [9]), 24.95 (CH_2, [10]), 22.68 (CH_2, [11]), 18.12 (CH_3, [12]), 14.14 (CH_3, [13]). **HRMS (ESI) *m/z*:** [M+**L1a**+H]$^+$ for $[C_{32}H_{56}N_3O_6]^+$; *calculated*: 578.4164, *found*: 578.4183. Submitted as the Diels-Alder adduct upon reaction with TAD-COOH **L1a** as a result of fragmentation arising during HRMS. **IR (ATR platinum diamond):** *ν* [cm^{-1}] = 3342 & 2908 (C-H), 1729 (C=O), 1596 (N-H), 1462 & 1392 (C-H), 1175 (C-O), 984 & 963 (C=C), 730 (C-H).

3.3.2 *Synthesis of aliphatic TAD-indole-based pin code (**PC1**)*

(i) 1st TAD addition and P-3CR

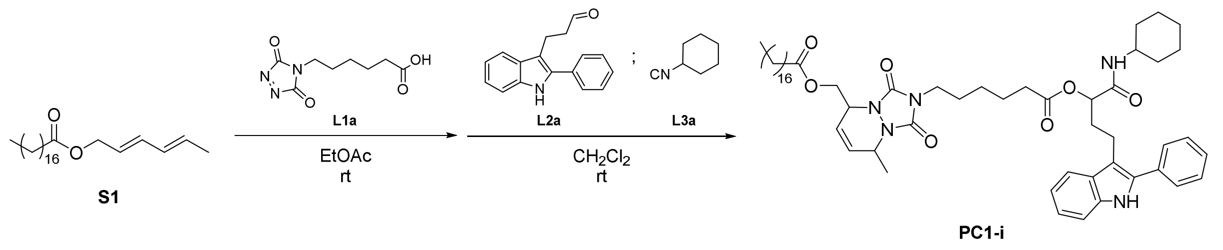

S1 (365 mg, 1.0 mmol, 1.0 equiv.) was dissolved in anhydrous ethyl acetate (3 mL), to which a solution of TAD-COOH **L1a** (320 mg, 1.5 mmol, 1.5 equiv.), dissolved in anhydrous ethyl acetate (2 mL) was added dropwise until the pink color persisted (ca. 1 hour), indicating that the diene was completely consumed. Next, 2,3-dimethylbut-2-ene (60 µL, 0.5 mmol, 0.5 equiv.) was added to the reaction mixture in order to quench the excess unreacted TAD. The solvent was removed *in vacuo* at 35 °C and the crude product was then used in the following step without purification. The resulting product was dissolved in anhydrous dichloromethane (4 mL). Subsequently, **L2a** (766 mg, 3.0 mmol, 2.0 equiv.*) was solubilized in dichloromethane (3 mL) and added to the reaction mixture, followed by cyclohexyl isocyanide **L3a** (280 µL, 2.25 mmol, 1.5 equiv.*). The reaction mixture was stirred at room temperature for 24 hours and the solvent was removed *in vacuo* at < 35 °C. The crude product was purified by column chromatography (silica, hexane:ethyl acetate 2:1 → 1:4), R_F (hexane: ethyl acetate 2:1 = 0.07), yielding **PC1-i** as a viscous yellow oil that solidified upon further drying *in vacuo*. Yield = 89 % (833 mg). *with respect to the amount of carboxylic acid (from the TAD-COOH) initially used.

^{1}H-NMR (400 MHz, DMSO-*d₆*, see Figure S36): δ= 11.15 (s, 1H, [1]), 7.81 (d, J = 8.0 Hz, 1H, [2]), 7.62-7.58 (m, 2H, [3]), 7.53-7.47 (m, 3H, [4+5]), 7.40-7.34 (m, 2H, [6+7]), 7.10 (m, 1H, [8]), 7.00 (m, 1H, [9]), 5.91 (m, 1H, [10]), 5.82 (m, 1H, [11]), 4.89 (t, J = 6.0 Hz, 1H, [12]), 4.62 (m, 1H, [13]), 4.49 (dd, J = 11.7, 3.6 Hz, 1H, [14]), 4.29 (m, 1H, [15]), 4.19 (ddd, J = 11.6, 5.1, 0.9 Hz, 1H, [16]), 3.58-3.51 (m, 1H, [17]), 3.40 (t, J = 7.1 Hz, 2H, [18]), 2.88 (m, 2H, [19]), 2.34 (m, 2H, [20]), 2.21 (t, J = 7.4 Hz, 2H, [21]), 2.04 (dt, J = 9.5, 6.4 Hz, 2H, [22]), 1.77-1.49 (m, 10H, [23+24+25]), 1.45 (t, J = 7.0 Hz, 2H, [26]), 1.40 (dd, J = 6.6, 0.6 Hz, 3H, [27]), 1.37-1.03 (m, 35H, [28+29+30]), 0.88-0.81 (m, 3H, [31]). **^{13}C-NMR (APT, 100 MHz, DMSO-*d₆*, see Figure S36):** δ= 172.56 (C, [1]), 172.14 (C, [2]), 168.05 (C, [3]), 153.74 (C, [4]), 151.90 (C, [4]), 136.02 (C, [5]), 133.96 (C, [6]), 132.83 (C, [7]), 129.74 (CH, [8]), 128.62 (CH, [9]), 128.40 (C, [10]), 127.63 (CH, [11]), 127.18 (CH, [12]), 121.50 (CH, [13]), 120.98 (CH, [14]), 118.66 (CH, [15]), 118.19 (CH, [16]), 111.19 (CH, [17]), 110.54 (C, [18]), 72.96 (CH, [19]), 61.86 (CH_2, [20]), 52.12 (CH, [21]), 50.61 (CH, [22]), 47.46 (CH, [23]), 38.11 (CH_2, [24]), 33.20 (CH_2, [25]), 32.79 (CH_2, [26]), 32.23 (CH_2, [27]), 31.28 (CH_2, [28]), 28.99-28.35 (CH_2, [29]), 27.07 (CH_2, [30]), 25.36 (CH_2, [31]), 25.15 (CH_2, [32]), 24.57 (CH_2, [33]), 24.09 (CH_2, [34]), 23.89 (CH_2, [35]), 22.07 (CH_2, [36]), 19.81 (CH_2, [37]), 18.89 (CH_3, [38]), 13.92 (CH_3, [39]). **HRMS (ESI) *m/z*:** $[M+H]^+$ for $[C_{56}H_{82}N_5O_7]^+$; *calculated*: 936.6209, *found*: 936.6234.

(ii) 2nd TAD addition and P-3CR

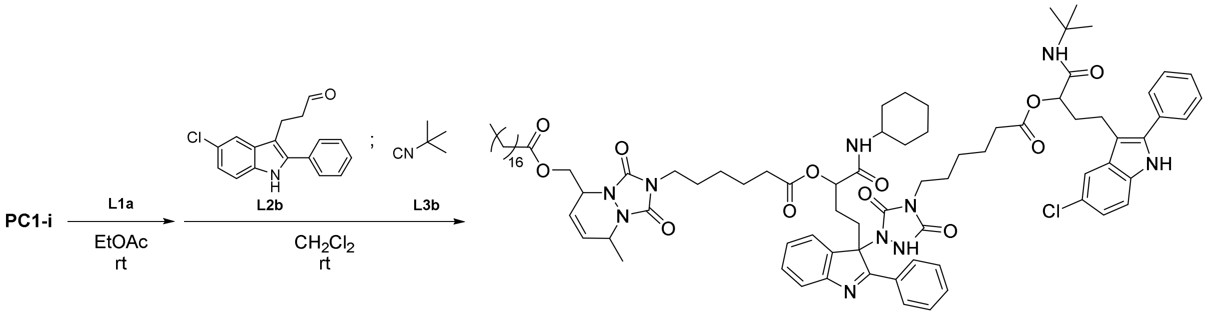

PC1-ii

PC1-i (809 mg, 0.86 mmol, 1.0 equiv.) was dissolved in anhydrous ethyl acetate (3 mL), to which a solution of TAD-COOH **L1a** (275 mg, 1.29 mmol, 1.5 equiv.), dissolved in anhydrous ethyl acetate (2.5 mL) was added dropwise until the pink color persisted (ca. 1 hour), indicating that the indole was completely consumed. Next, 2,3-dimethylbut-2-ene (51 µL, 0.43 mmol, 0.5 equiv.) was added to the reaction mixture in order to quench the excess unreacted TAD. The solvent was removed *in vacuo* at 35 °C and the crude product was then used in the following step without purification. The resulting product was dissolved in anhydrous dichloromethane (3 mL). Subsequently, **L2b** (738 mg, 2.6 mmol, 2.0 equiv.*) was solubilized in dichloromethane (2 mL) and added to the reaction mixture, followed by *tert*-butyl isocyanide **L3b** (221 µL, 1.95 mmol, 1.5 equiv.*). The reaction mixture was stirred at room temperature for 22 hours and the solvent was removed *in vacuo* at < 35 °C. The crude product was purified by column chromatography (silica, hexane:ethyl acetate 9:1 → 1:1), R_F (hexane: ethyl acetate 1:4 = 0.51), yielding **PC1-ii** as a viscous yellow oil that solidified upon further drying *in vacuo*. Yield = 86 % (1.127 g).

*with respect to the amount of carboxylic acid (from the TAD-COOH) initially used.

^{1}H-NMR (400 MHz, DMSO-*d$_6$*, see Figure S37): δ = 11.38 (s, 1H, [1]), 10.70 (s, 1H, [2]), 8.19 (m, 2H, [3]), 7.60 (d, *J* = 8.2 Hz, 2H, [4]), 7.57-7.44 (m, 9H, [5]), 7.44-7.33 (m, 4H, [6]), 7.24 (m, 1H, [7]), 7.10 (dd, *J* = 8.5, 2.0 Hz, 1H, [8]), 5.92 (m, 1H, [9]), 5.82 (m, 1H, [10]), 4.85 (t, *J* = 5.9 Hz, 1H, [11]), 4.63 (m, 1H, [12]), 4.56 (m, 1H, [13]), 4.49 (dd, *J* = 11.6, 3.4 Hz, 1H, [14]), 4.31 (m, 1H, [15]), 4.21 (dd, *J* = 11.7, 4.9 Hz, 1H, [16]), 3.38 (m, 2H, [17]), 3.30-3.10 (m, 3H, [18]), 2.84 (m, 2H, [19]), 2.41 (m, 2H, [20]), 2.21 (t, *J* = 7.3 Hz, 2H, [21]), 2.17-2.06 (m, 4H, [22]), 2.01 (m, 2H, [23]), 1.65-1.28 (m, 16H, [24]), 1.28-0.87 (m, 50H, [25]), 0.84 (m, 3H, [26]). **^{13}C-NMR (APT, 100 MHz, DMSO-*d$_6$*):** δ = 174.54 (C), 172.57 (C), 171.96 (C), 171.74 (C), 171.69 (C), 168.36 (C), 167.13 (C), 167.07 (C), 155.16 (C), 155.06 (C), 153.78 (C), 153.72 (C), 153.66 (C), 151.99 (C), 136.86 (C), 136.79 (C), 135.79 (C), 134.46 (C), 132.29 (C), 131.94 (C), 131.85 (C), 131.21 (CH), 129.82 (CH), 129.69 (CH), 129.58 (C), 128.73 (CH), 128.62 (CH), 127.73 (CH), 127.68 (CH), 126.56 (CH), 123.33 (C), 121.41 (CH), 121.25 (CH), 121.02 (CH), 120.64 (CH), 117.34 (CH), 112.76 (CH), 110.47 (C), 76.08 (C), 73.05 (CH), 71.55 (CH), 71.46 (CH), 61.88 (CH_2), 52.25 (CH), 50.57 (CH), 50.21 (C), 47.31 (CH), 38.10 (CH_2), 38.01 (CH_2), 33.26 (CH_2), 33.17 (CH_2), 33.03 (CH_2), 32.69 (CH_2), 32.10 (CH_2), 31.91 (CH_2), 31.27 (CH_2), 29.00-28.58 (CH_2), 28.41 (CH_3), 28.36 (CH_2),

27.03 (CH_2), 26.60 (CH_2), 25.29 (CH_2), 25.10 (CH_2), 24.97 (CH_2), 24.46 (CH_2), 24.11 (CH_2), 23.65 (CH_2), 22.07 (CH_2), 19.66 (CH_2), 18.88 (CH_3), 13.91 (CH_3). **HRMS (ESI) *m/z*:** $[M+H]^+$ for $[C_{86}H_{116}ClN_{10}O_{12}]^+$; *calculated*: 1515.8457, *found*: 1515.8448.

(iii) 3rd TAD addition and P-3CR

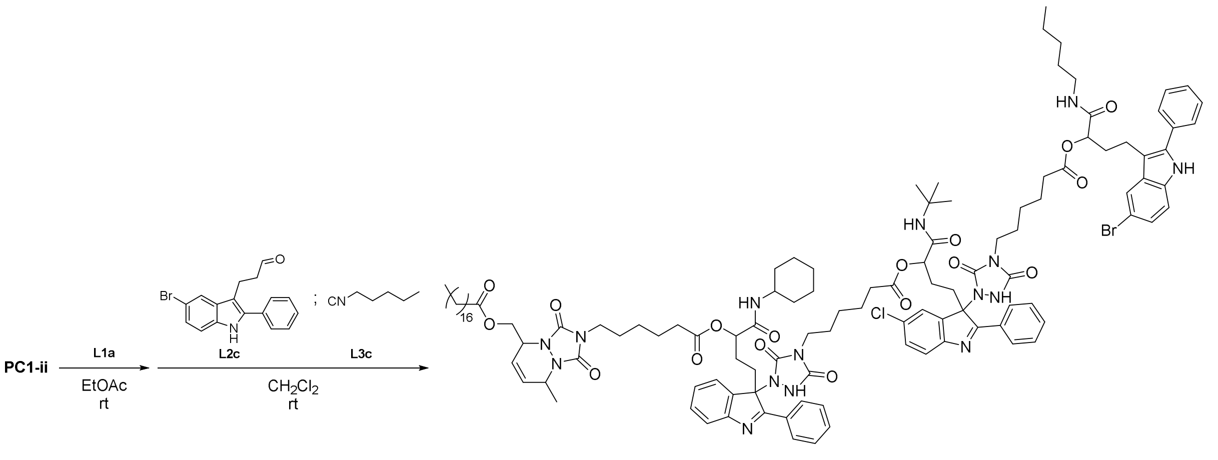

PC1-ii (1.25 g, 0.82 mmol, 1.0 equiv.) was dissolved in anhydrous ethyl acetate (4 mL), to which a solution of TAD-COOH **L1a** (262 mg, 1.23 mmol, 1.5 equiv.), dissolved in anhydrous ethyl acetate (2.5 mL) was added dropwise until the pink color persisted (ca. 1 hour), indicating that the indole was completely consumed. Next, 2,3-dimethylbut-2-ene (49 µL, 0.41 mmol, 0.5 equiv.) was added to the reaction mixture in order to quench the excess unreacted TAD. The solvent was removed *in vacuo* at 35 °C and the crude product was then used in the following step without purification. The resulting product was dissolved in anhydrous dichloromethane (4 mL). Subsequently, **L2c** (807 mg, 2.46 mmol, 2.0 equiv.*) was solubilized in dichloromethane (3 mL) and added to the reaction mixture, followed by 1-pentyl isocyanide **L3c** (232 µL, 1.85 mmol, 1.5 equiv.*). The reaction mixture was stirred at room temperature for 48 hours and then the solvent was removed *in vacuo* at < 35 °C. The crude product was purified by column chromatography (silica, hexane:ethyl acetate 4:1 → 0:1), R_F (ethyl acetate = 0.48), yielding **PC1-iii** as a viscous yellow oil that solidified upon further drying *in vacuo*. Yield = 49 % (868 mg).

*with respect to the amount of carboxylic acid (from the TAD-COOH) initially used.

^{1}H-NMR (400 MHz, DMSO-d_6, see Figure S38): δ = 11.40 (s, 1H, [1]), 10.70 (s, 2H, [2]), 8.18 (m, 4H, [3]), 7.93 (m, 1H, [4]), 7.66 (d, J = 1.6 Hz, 1H, [5]), 7.63-7.34 (m, 18H, [6]), 7.32 (d, J = 8.4 Hz, 1H, [7]), 7.28-7.17 (m, 3H, [8]), 5.92 (m, 1H, [9]), 5.83 (m, 1H, [10]), 4.84 (m, 1H, [11]), 4.64 (m, 1H, [12]), 4.60-4.52 (m, 2H, [13]), 4.50 (dd, J = 11.7, 3.6 Hz, 1H, [14]), 4.31 (m, 1H, [15]), 4.22 (dd, J = 11.7, 4.9 Hz, 1H, [16]), 3.38 (m, 2H, [17]), 3.30-3.11 (m, 5H, [18]), 3.05 (m, 2H, [19]), 2.85 (m, 2H, [20]), 2.44 (m, 2H, [21]), 2.27-1.86 (m, 12H, [22]), 1.68-1.28 (m, 22H, [23]), 1.28-0.87 (m, 58H, [24]), 0.84-0.74 (m, 6H, [25]). **^{13}C-NMR (APT, 100 MHz, DMSO-d_6):** δ = 175.42 (C), 174.54 (C), 172.57 (C), 172.02 (C), 171.75 (C), 171.69 (C), 171.58 (C),

171.50 (C), 168.97 (C), 167.35 (C), 167.22 (C), 167.14 (C), 167.08 (C), 155.29 (C), 155.17 (C), 155.05 (C), 155.03 (C), 154.98 (C), 153.79 (C), 153.72 (C), 153.65 (C), 152.61 (C), 152.55 (C), 152.00 (C), 138.92 (C), 136.86 (C), 136.79 (C), 135.71 (C), 134.67 (C), 132.23 (C), 131.95 (C), 131.85 (C), 131.61 (CH), 131.56 (C), 131.52 (C), 131.21 (CH), 130.91 (C), 130.26 (C), 129.91 (CH), 129.81 (CH), 129.68 (CH), 128.70 (CH), 128.62 (CH), 127.77 (CH), 127.68 (CH), 126.55 (CH), 123.96 (CH), 122.03 (CH), 121.32 (CH), 121.03 (CH), 120.65 (CH), 120.44 (CH), 113.20 (CH), 111.30 (C), 110.21 (C), 76.18 (C), 76.15 (C), 76.07 (C), 72.93 (CH), 71.58 (CH), 71.51 (CH), 71.46 (CH), 71.29 (CH), 61.88 (CH_2), 59.73 (CH_2), 52.26 (CH), 50.57 (CH), 50.08 (C), 50.01 (C), 47.32 (CH), 38.26 (CH_2), 38.11 (CH_2), 38.01 (CH_2), 33.27 (CH_2), 33.05 (CH_2), 32.98 (CH_2), 32.64 (CH_2), 32.12 (CH_2), 32.00 (CH_2), 31.96 (CH_2), 31.92 (CH_2), 31.27 (CH_2), 29.00-28.64 (CH_2), 28.46 (CH_2), 28.37 (CH_2), 28.26 (CH_3), 28.16 (CH_3), 27.04 (CH_2), 26.57 (CH_2), 25.30 (CH_2), 25.11 (CH_2), 25.07 (CH_2), 24.99 (CH_2), 24.91 (CH_2), 24.89 (CH_2), 24.47 (CH_2), 24.12 (CH_2), 23.70 (CH_2), 23.66 (CH_2), 23.57 (CH_2), 23.45 (CH_2), 23.42 (CH_2), 22.08 (CH_2), 21.75 (CH_2), 19.65 (CH_2), 18.88 (CH_3), 13.91 (CH_3), 13.84 (CH_3). **HRMS (ESI) *m/z*:** $[M+H]^+$ for $[C_{117}H_{152}BrClN_{15}O_{17}]^+$; *calculated*: 2153.0357, *found*: 2153.0370.

(iv) 4th TAD addition and P-3CR

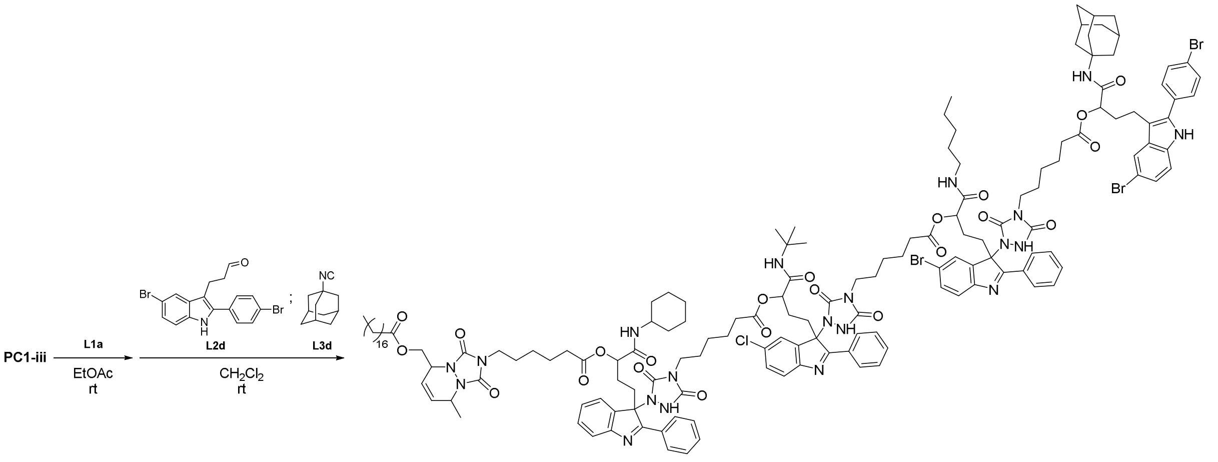

PC1-iii (800 mg, 0.371 mmol, 1.0 equiv.) was dissolved in anhydrous ethyl acetate (3 mL), to which a solution of TAD-COOH **L1a** (119 mg, 0.557 mmol, 1.5 equiv.), dissolved in anhydrous ethyl acetate (1 mL) was added dropwise until the pink color persisted (ca. 1 hour), indicating that the indole was completely consumed. Next, 2,3-dimethylbut-2-ene (22 µL, 0.186 mmol, 0.5 equiv.) was added to the reaction mixture in order to quench the excess unreacted TAD. The solvent was removed *in vacuo* at 35 °C and the crude product was then used in the following step without purification. The resulting product was dissolved in anhydrous dichloromethane (3 mL). Subsequently, **L2d** (454 mg, 1.14 mmol, 2.0 equiv.*) was solubilized in dichloromethane (1 mL) and added to the reaction mixture, followed by 1-adamantyl isocyanide **L3d** (135 mg, 0.836 mmol, 1.5 equiv.*) solubilized in dichloromethane (1 mL).

The reaction mixture was stirred at room temperature for 48 hours and the solvent was removed *in vacuo* at < 35 °C. The crude product was purified by column chromatography (silica, hexane:ethyl acetate 2:1 → 0:1, R_F (ethyl acetate = 0.43), yielding a viscous yellow oil that solidified upon further drying *in vacuo*. Yield = 83 % (902 mg). From SEC analysis, a small amount of the remaining trimer was observed to have co-eluted. Therefore, the product was re-purified by column chromatography (silica, hexane:ethyl acetate 9:1 > 0:1 gradient), R_F (hexane:ethyl acetate 1:9) = 0.38) to give the aliphatic pin code **PC1** as a faint yellow powder. Yield = 55 % (596 mg).[§]

*with respect to the amount of carboxylic acid (from the TAD-COOH) initially used.

[§]determined based upon the purity (LCMS) of the isolated fractions obtained after column chromatography.

^{1}H-NMR (400 MHz, DMSO-*d*$_6$, see Figure S39): δ = 11.46 (s, 1H, [1]), 10.75 (s, 3H, [2]), 8.18 (m, 6H, [3]), 7.75-7.28 (m, 27H, [4]), 7.22 (m, 3H, [5]), 5.92 (m, 1H, [6]), 5.84 (m, 1H, [7]), 4.81 (t, *J* = 5.7 Hz, 1H, [8]), 4.64 (m, 1H, [9]), 4.61-4.52 (m, 3H, [10]), 4.49 (dd, *J* = 11.7, 3.5 Hz, 1H, [11]), 4.31 (m, 1H, [12]), 4.21 (dd, *J* = 11.7, 4.9 Hz, 1H, [13]), 3.37 (m, 2H, [14]), 3.29-3.09 (m, 7H, [15]), 2.83 (m, 4H, [16]), 2.48-2.29 (m, 2H, [17]), 2.27-1.82 (m, 25H, [18]), 1.82-1.28 (m, 32H, [19]), 1.28-0.87 (m, 62H, [20]), 0.87-0.75 (m, 6H, [21]). **^{13}C-NMR (APT, 100 MHz, DMSO-*d*$_6$):** δ = 175.47 (C), 175.27 (C), 174.59 (C), 172.58 (C), 171.90 (C), 171.77 (C), 171.69 (C), 171.66 (C), 171.60 (C), 171.51 (C), 168.08 (C), 168.03 (C), 167.90 (C), 167.38 (C), 167.24 (C), 167.16 (C), 167.09 (C), 155.21 (C), 155.08 (C), 154.97 (C), 153.78 (C), 153.73 (C), 153.66 (C), 152.95 (C), 152.90 (C), 152.61 (C), 152.54 (C), 152.00 (C), 134.77 (C), 134.46 (C), 131.96 (C), 131.86 (C), 131.69 (CH), 131.63 (CH), 131.46 (C), 131.24 (CH), 130.92 (C), 130.17 (C), 129.93 (CH), 129.80 (CH), 129.70 (CH), 128.73 (CH), 128.62 (CH), 127.72 (CH), 127.68 (CH), 126.57 (CH), 124.26 (CH), 122.48 (CH), 122.00 (CH), 121.33 (CH), 121.03 (CH), 120.98 (C), 120.65 (CH), 120.55 (CH), 119.20 (C), 113.33 (CH), 111.42 (C), 110.97 (C), 76.10 (C), 74.20 (CH_2), 72.89 (CH), 71.81 (CH), 71.59 (CH), 71.47 (CH), 68.76 (CH), 61.89 (CH_2), 59.74 (CH_2), 58.29 (CH), 52.26 (CH), 50.92 (CH_2), 50.58 (CH), 50.09 (C), 50.02 (C), 47.33 (CH), 40.91 (CH_2), 38.12 (CH_2), 35.97 (CH_2), 33.28 (CH_2), 33.18 (CH_2), 33.04 (CH_2), 32.98 (CH_2), 32.90 (CH_2), 32.61 (CH_2), 32.59 (CH_2), 32.12 (CH_2), 32.01 (CH_2), 31.96 (CH_2), 31.93 (CH_2), 31.28 (CH_2), 31.13 (CH), 30.36 (CH_2), 29.81 (CH_2), 29.00-28.88 (CH_2), 28.69-28.60 (CH_2), 28.49 (CH_2), 28.45 (CH_2), 28.38 (CH_2), 28.27 (CH_3), 28.16 (CH_3), 27.05 (CH_2), 26.60 (CH_2), 25.31 (CH_2), 25.10 (CH_2), 24.96 (CH_2), 24.90 (CH_2), 24.48 (CH_2), 24.12 (CH_2), 23.70 (CH_2), 23.66 (CH_2), 23.40 (CH_2), 22.08 (CH_2), 21.73 (CH_2), 21.68 (CH_2), 20.74, 18.88 (CH_3), 13.92 (CH_3), 13.84 (CH_3). **HRMS (ESI) *m/z*:** $[M+H]^+$ for $[C_{153}H_{192}Br_3ClN_{20}O_{22}]^{2+}$; *calculated*: 1466.5874, *found*: 1466.5899.

*3.3.3 Synthesis of aromatic TAD-indole-based pin code (**PC2**)*

(i)   1st TAD addition and P-3CR

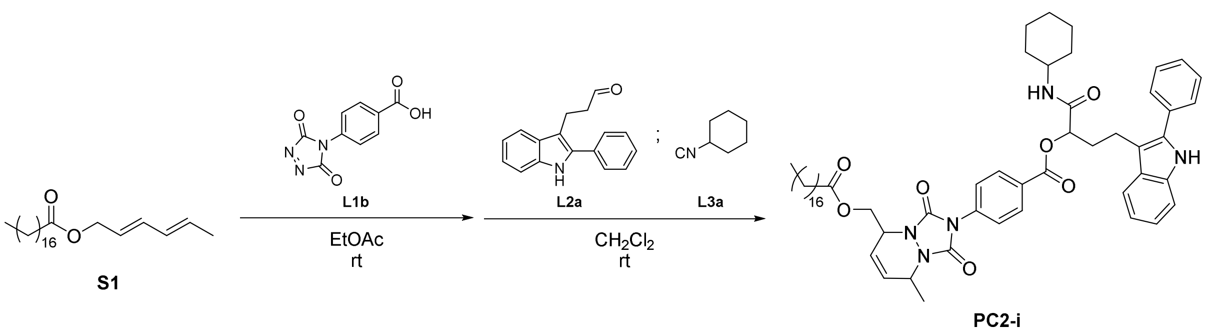

S1 (365 mg, 1.0 mmol, 1.0 equiv.) was dissolved in anhydrous ethyl acetate (3 mL), to which a solution of TAD-COOH **L1b** (163 mg, 1.2 mmol, 1.2 equiv.) dissolved in anhydrous ethyl acetate (3.5 mL) was added dropwise until the pink color persisted (ca. 1 hour), indicating the complete consumption of the diene. Next, 2,3-dimethylbut-2-ene (36 µL, 0.3 mmol, 0.3 equiv.) was added to the reaction mixture in order to quench the excess unreacted TAD. The solvent was removed *in vacuo* at 35 °C and the crude product was then used in the following step without purification. The resulting product was dissolved in anhydrous dichloromethane (3 mL). Subsequently, **L2a** (499 mg, 2.0 mmol, 2.0 equiv.*) was solubilized in dichloromethane (1 mL) and added to the reaction mixture, followed by cyclohexyl isocyanide **L3a** (187 µL, 1.5 mmol, 1.5 equiv.*). The reaction mixture was stirred at room temperature for 22 hours and then the solvent was removed *in vacuo* at < 35 °C. The crude product was purified by column chromatography (silica, hexane:ethyl acetate 9:1 → 0:1), R_F (hexane: ethyl acetate 4:1 = 0.11), yielding **PC2-i** as a viscous orange oil that solidified upon further drying *in vacuo*. Yield = 97 % (1.122 g).

*with respect to the amount of carboxylic acid (from the TAD-COOH) initially used.

^{1}H-NMR (400 MHz, DMSO-d_6, see Figure S40): δ= 11.17 (s, 1H, [1]), 8.11 (d, J = 8.5 Hz, 2H, [2]), 8.01 (d, J = 7.8 Hz, 1H, [3]), 7.71 (m, 2H, [4]), 7.61 (m, 2H, [5]), 7.55 (m, 1H, [6]), 7.43 (m, 2H, [7]), 7.34 (m, 2H, [8+9]), 7.11 (m, 1H, [10]), 7.01 (m, 1H, [11]), 6.00 (m, 1H, [12]), 5.91 (m, 1H, [13]), 5.14 (m, 1H, [14]), 4.79 (m, 1H, [15]), 4.59 (dd, J = 11.7, 3.4 Hz, 1H, [16]), 4.49 (m, 1H, [17]), 4.28 (dd, J = 11.8, 4.9 Hz, 1H, [18]), 3.57 (m, 1H, [19]), 3.01 (m, 2H, [20]), 2.29-2.15 (m, 4H, [21+22]), 1.80-1.39 (m, 9H, [23-27]), 1.37-1.03 (m, 34H, [28-30]), 0.83 (t, J = 7.1 Hz, 3H, [31]). **^{13}C-NMR (APT, 100 MHz, DMSO-d_6, see Figure S40):** δ= 172.84 (C, [1]), 167.81 (C, [2]), 164.42 (C, [3]), 151.82 (C, [4]), 150.22 (C, [4]), 136.06 (C, [5]), 135.84 (C, [6]), 134.08 (C, [7]), 132.84 (C, [8]), 129.97 (CH, [9]), 129.66 (CH, [10]), 128.60 (CH, [11]), 128.56 (C, [12]), 128.43 (C, [13]), 127.67 (CH, [14]), 127.23 (CH, [15]), 125.51 (CH, [16]), 121.53 (CH, [17]), 120.73 (CH, [18]), 118.68 (CH, [19]), 118.25 (CH, [20]), 111.24 (CH, [21]), 110.52 (C, [22]), 74.01 (CH, [23]), 61.85 (CH_2, [24]), 52.44 (CH, [25]), 50.93 (CH, [26]), 47.60 (CH, [27]), 33.31 (CH_2, [28]), 32.98 (CH_2, [29]), 32.32 (CH_2, [30]), 32.22 (CH_2, [30]), 31.26 (CH_2, [31]), 28.97-28.37 (CH_2, [32]), 25.17 (CH_2, [33]), 24.60 (CH_2, [34]), 24.11 (CH_2, [35]), 22.07 (CH_2, [36]), 19.96 (CH_2, [37]), 19.20 (CH_3,

[38]), 13.92 (CH_3, [39]). **HRMS (ESI) *m/z*:** $[M+H]^+$ for $[C_{57}H_{76}N_5O_7]^+$; *calculated*: 942.5739, *found*: 942.5737.

(ii) 2nd TAD addition and P-3CR

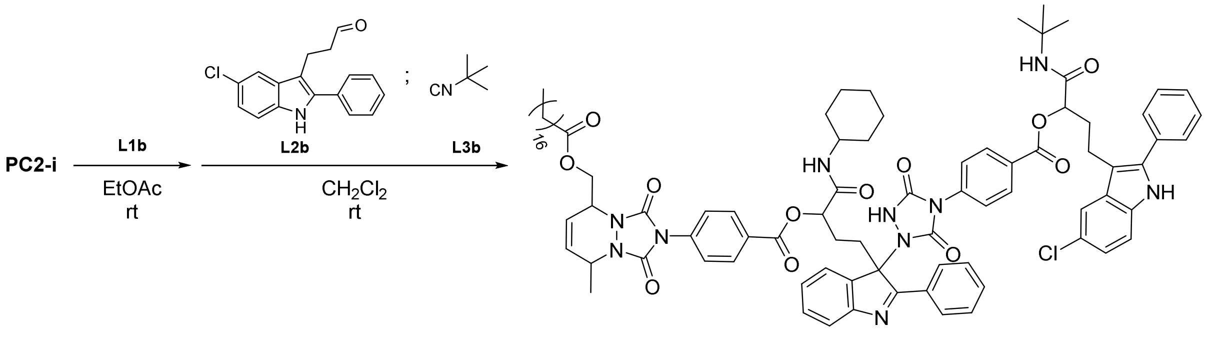

PC2-i (1.1 g, 1.17 mmol, 1.0 equiv.) was dissolved in anhydrous ethyl acetate (3.5 mL), to which a solution of TAD-COOH, **L1b** (308 mg, 1.40 mmol, 1.2 equiv.), dissolved in anhydrous ethyl acetate (4 mL) was added dropwise until the pink color persisted (ca. 15 min), indicating that the indole was completely consumed. Next, 2,3-dimethylbut-2-ene (69 µL, 0.585 mmol, 0.5 equiv.) was added to the reaction mixture in order to quench the excess unreacted TAD. The solvent was removed *in vacuo* at 30 °C and the crude product was then used in the following step without purification. The resulting product was solubilized along with **L2b** (797 mg, 2.808 mmol, 2.0 equiv.*) in dichloromethane (5 mL). *tert*-Butyl isocyanide **L3b** (238 µL, 2.106 mmol, 1.5 equiv.*) was then added and the reaction mixture was stirred at room temperature for 36 hours and the solvent was removed *in vacuo* at 30 °C. The crude product was purified by column chromatography (silica, hexane:ethyl acetate 9:1 → 0:1, with the product eluting approximately in hexane:ethyl acetate 2:1), R_F (hexane: ethyl acetate 1:4 = 0.72), yielding **PC2-ii** as a viscous yellow oil that solidified upon further drying *in vacuo*. Yield = 51 % (905 mg).

*with respect to the amount of carboxylic acid (from the TAD-COOH) initially used.

^{1}H-NMR (400 MHz, DMSO-d_6, see Figure S41): δ = 11.41 (s, 1H, [1]), 11.38 (s, 1H, [2]), 8.32 (m, 2H, [3]), 7.97 (d, *J* = 8.4 Hz, 2H, [4]), 7.85 (d, *J* = 8.5 Hz, 2H, [5]), 7.79-7.52 (m, 12H, [6]), 7.52-7.26 (m, 8H, [7]), 7.09 (dd, *J* = 8.6, 2.0 Hz, 1H, [8]), 6.00 (m, 1H, [9]), 5.91 (m, 1H, [10]), 5.07 (t, *J* = 5.8 Hz, 1H, [11]), 4.89-4.79 (m, 2H, [12+13]), 4.61 (m, 1H, [14]), 4.50 (m, 1H, [15]), 4.30 (dd, *J* = 11.8, 3.5 Hz, 1H, [16]), 3.48 (m, 1H, [17]), 3.01-2.54 (m, 4H, [18+19]), 2.26 (t, *J* = 7.3 Hz, 2H, [20]), 2.14 (m, 2H, [21]), 1.72-1.42 (m, 9H, [22]), 1.42-0.89 (m, 45H, [23]), 0.82 (t, *J* = 6.8 Hz, 3H, [24]). **^{13}C-NMR (APT, 100 MHz, DMSO-d_6):** δ = 174.38 (C), 172.84 (C), 168.06 (C), 167.01 (C), 166.73 (C), 164.23 (C), 164.04 (C), 163.91 (C), 153.83 (C), 153.74 (C), 151.72 (C), 150.26 (C), 136.86 (C), 135.90 (C), 135.85 (C), 135.33 (C), 134.47 (C), 132.28 (C), 131.78 (C), 131.40 (CH), 130.07 (CH), 129.87 (CH), 129.58 (C), 128.76 (CH), 128.69 (CH), 128.22 (C), 127.72 (CH), 126.97 (CH), 125.46 (CH), 125.02 (CH), 123.34 (C), 121.51 (CH), 121.41 (CH),

120.84 (CH), 120.76 (CH), 117.38 (CH), 112.77 (CH), 110.43 (C), 75.89 (C), 74.20 (CH_2), 74.05 (CH), 72.63 (CH), 72.18 (CH), 68.76 (CH), 61.87 (CH_2), 52.50 (CH), 50.86 (CH), 50.31 (C), 47.44 (CH), 47.38 (CH), 33.33 (CH_2), 32.84 (CH_2), 32.25 (CH_2), 32.02 (CH_2), 31.96 (CH_2), 31.28 (CH_2), 28.99 (CH_2), 28.94 (CH_2), 28.81 (CH_2), 28.69 (CH_2), 28.67 (CH_2), 28.43 (CH_3), 25.14 (CH_2), 24.43 (CH_2), 24.15 (CH_2), 22.08 (CH_2), 19.84 (CH_2), 19.17 (CH_3), 13.91 (CH_3). **HRMS (ESI)** ***m/z*:** $[M+H]^+$ for $[C_{88}H_{104}ClN_{10}O_{12}]^+$; *calculated*: 1527.7518, *found*: 1527.7585.

(iii) 3rd TAD addition and P-3CR

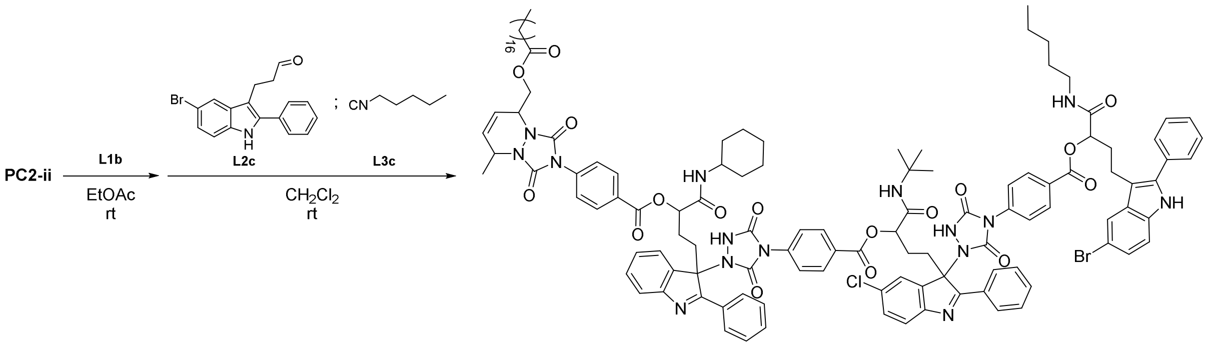

PC2-iii

PC2-ii (855 mg, 0.559 mmol, 1.0 equiv.) was dissolved in anhydrous ethyl acetate (4 mL), to which a solution of TAD-COOH **L1b** (147 mg, 0.671 mmol, 1.2 equiv.), dissolved in anhydrous ethyl acetate (3 mL) was added dropwise until the pink color persisted (ca. 2.5 hours), indicating that the indole was completely consumed. Next, 2,3-dimethylbut-2-ene (33 µL, 0.28 mmol, 0.5 equiv.) was added to the reaction mixture in order to quench the excess unreacted TAD. The solvent was removed *in vacuo* at 30 °C and the crude product was then used in the following step without purification. The resulting product was dissolved in anhydrous dichloromethane (3 mL). Subsequently, **L2c** (440 mg, 1.342 mmol, 2.0 equiv.*) was solubilized in dichloromethane (2 mL) and added to the reaction mixture, followed by 1-pentyl isocyanide **L3c** (127 µL, 1.007 mmol, 1.5 equiv.*). The reaction mixture was stirred at room temperature for 48 hours and the solvent was removed *in vacuo* at 30 °C. The crude product was purified by column chromatography (silica, hexane:ethyl acetate 4:1 → 0:1), R_F (hexane: ethyl acetate 1:2 = 0.47), yielding a pale yellow powder. A less pure fraction was recovered and re-purified by column chromatography, yielding an additional batch of **PC2-iii** as a pale yellow powder. Combined yield = 61 % (736 mg).[§]

*with respect to the amount of carboxylic acid (from the TAD-COOH) initially used.

[§]determined based upon the purity (LCMS) of the isolated fractions obtained after column chromatography.

^{1}H-NMR (400 MHz, DMSO-*d₆*, see Figure S42): δ = 11.42 (s, 2H, [1]), 11.40 (s, 1H, [2]), 8.32 (m, 2H, [3]), 8.25 (d, *J* = 7.4 Hz, 2H, [4]), 8.11 (t, *J* = 5.6 Hz, 1H, [5]), 7.98 (d, *J* = 8.7 Hz, 2H, [6]), 7.85 (m, 2H, [7]), 7.81-7.22 (m, 30H, [8]), 7.20 (dd, *J* = 8.5, 1.8 Hz, 1H, [9]), 5.98 (m, 1H, [10]), 5.91 (m, 1H, [11]), 5.06 (t, *J* =

6.0 Hz, 1H, [12]), 4.89-4.72 (m, 3H, [13+14]), 4.61 (m, 1H, [15]), 4.50 (m, 1H, [16]), 4.30 (dd, *J* = 11.8, 3.4 Hz, 1H, [17]), 3.47 (m, 1H, [18]), 3.15-2.86 (m, 4H, [19+20]), 2.86-2.53 (m, 4H, [21]), 2.26 (t, *J* = 7.2 Hz, [22]), 2.17 (m, 2H, [23]), 1.73-1.32 (m, 9H, [24]), 1.42-0.95 (m, 53H, [25]), 0.90-0.71 (m, 6H, [26]). **^{13}C-NMR (APT, 100 MHz, DMSO-*d*$_6$):** δ = 175.23 (C), 174.39 (C), 172.83 (C), 169.84 (C), 168.70 (C), 167.10 (C), 167.01 (C), 166.90 (C), 166.73 (C), 164.30 (C), 164.04 (C), 163.90 (C), 163.81 (C), 163.71 (C), 153.82 (C), 153.74 (C), 153.14 (C), 153.04 (C), 152.52 (C), 152.42 (C), 151.70 (C), 150.26 (C), 149.90 (C), 135.89 (C), 135.86 (C), 135.78 (C), 134.67 (C), 132.17 (C), 131.78 (C), 131.22 (C), 130.25 (C), 130.00 (CH), 129.89 (CH), 129.78 (CH), 129.56 (CH), 128.88 (CH), 128.76 (CH), 128.65 (CH), 128.22 (C), 128.19 (C), 127.77 (CH), 127.73 (CH), 127.63 (CH), 126.94 (CH), 125.50 (CH), 125.45 (CH), 125.01 (CH), 123.96 (CH), 122.10 (CH), 121.77 (CH), 121.62 (CH), 121.51 (CH), 121.37 (CH), 120.76 (CH), 120.46 (CH), 113.20 (CH), 111.30 (C), 110.15 (C), 75.86 (C), 74.19 (CH_2), 74.01 (CH), 72.63 (CH), 72.18 (CH), 68.75 (CH), 61.87 (CH_2), 58.28 (CH), 52.50 (CH), 50.83 (CH), 50.21 (C), 50.12 (C), 47.43 (CH), 38.32 (CH_2), 33.32 (CH_2), 32.74 (CH_2), 32.24 (CH_2), 31.96 (CH_2), 31.27 (CH_2), 31.12 (CH), 30.41 (CH), 29.81 (CH), 29.01-28.88 (CH_2), 28.80 (CH_2), 28.68 (CH_2), 28.65 (CH_2), 28.52 (CH), 28.47 (CH_2), 28.39 (CH_2), 28.35 (CH_3), 28.21 (CH_3), 25.09 (CH_2), 24.42 (CH_2), 24.14 (CH_2), 22.07 (CH_2), 21.73 (CH_2), 19.77 (CH_2), 19.16 (CH_3), 16.40 (CH_3), 13.91 (CH_3), 13.84 (CH_3). **HRMS (ESI) *m/z*:** $[M+H]^+$ for $[C_{120}H_{134}BrClN_{15}O_{17}]^+$; *calculated*: 2170.8949, *found*: 2170.9020.

(iv) 4th TAD addition and P-3CR

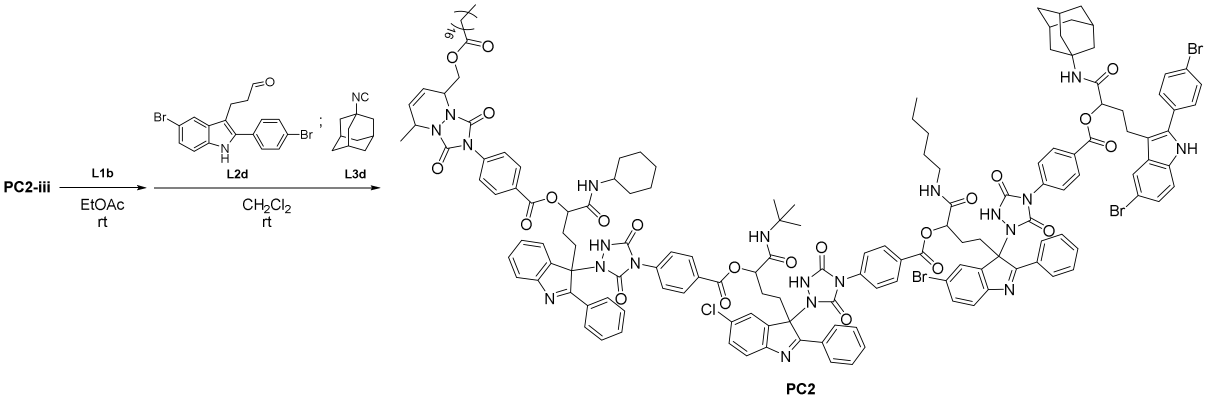

PC2-iii (267 mg, 0.123 mmol, 1.0 equiv.) was dissolved in anhydrous ethyl acetate (2 mL), to which a solution of TAD-COOH **L1b** (38 mg, 0.172 mmol, 1.4 equiv.), dissolved in anhydrous ethyl acetate (0.6 mL) was added dropwise until the pink color persisted (ca. 3 hours), indicating that the indole was completely consumed. Next, 2,3-dimethylbut-2-ene (8 µL, 0.062 mmol, 0.5 equiv.) was added to the reaction mixture in order to quench the excess unreacted TAD. The solvent was removed *in vacuo* at 30 °C and the crude product was then used in the following step without purification. The resulting product was dissolved in anhydrous dichloromethane (1 mL). Subsequently, **L2d** (140 mg, 0.343 mmol, 2.0 equiv.*) was solubilized in dichloromethane (0.6 mL) and added to the reaction mixture, followed

by 1-adamantyl isocyanide **L3d** (41 mg, 0.257 mmol, 1.5 equiv.*) solubilized in dichloromethane (0.3 mL). The reaction mixture was stirred at room temperature for 48 hours and the solvent was removed *in vacuo* at 30 °C. The crude product was purified by column chromatography (silica, hexane:ethyl acetate 9:1 → 1:2 gradient containing 1 % acetic acid, R_F (hexane:ethyl acetate 1:2) = 0.38) to give the aromatic TAD-based pin code **PC2** as a glossy orange powder. Yield = 78 % (280 mg).[§]

*with respect to the amount of carboxylic acid (from the TAD-COOH) initially used.

[§]determined based upon the purity (LCMS) of the isolated fractions obtained after column chromatography.

^{1}H-NMR (400 MHz, DMSO-d_6, see Figure S43): δ = 11.44 (s, 1H, [1]), 11.40 (s, 3H, [2]), 8.27 (m, 6H, [3]), 7.95 (m, 2H, [4]), 7.91-7.80 (m, 3H, [5]), 7.80-7.27 (m, 40H, [6]), 7.21 (dd, *J* = 8.5, 1.7 Hz, 1H, [7]), 5.99 (m, 1H, [8]), 5.90 (m, 1H, [9]), 5.01 (t, *J* = 5.1 Hz, 1H, [10]), 4.88-4.71 (m, 4H, [11+12]), 4.60 (m, 1H, [13]), 4.49 (m, 1H, [14]), 4.29 (dd, *J* = 11.5, 4.2 Hz, 1H, [15]), 3.45 (m, 1H, [16]), 3.09-2.53 (m, 10H, [17]), 2.25 (t, *J* = 7.2 Hz, 2H, [18]), 2.12 (m, 2H, [19]), 2.03-1.83 (m, 9H, [20]), 1.69-1.41 (m, 15H, [21]), 1.42-0.95 (m, 55H, [22]), 0.90-0.74 (m, 6H, [23]). **^{13}C-NMR (APT, 100 MHz, DMSO-d_6):** δ = 175.22 (C), 175.00 (C), 174.42 (C), 172.85 (C), 172.00 (C), 169.86 (C), 167.89 (C), 167.76 (C), 167.59 (C), 167.02 (C), 166.90 (C), 166.74 (C), 164.18 (C), 164.05 (C), 163.95 (C), 163.91 (C), 163.83 (C), 163.72 (C), 153.84 (C), 153.68 (C), 153.03 (C), 152.54 (C), 152.46 (C), 151.70 (C), 150.27 (C), 135.91 (C), 135.86 (C), 134.79 (C), 134.62 (C), 131.65 (CH), 131.42 (C), 131.39 (C), 131.29 (C), 130.17 (C), 130.09 (CH), 129.89 (CH), 129.83 (CH), 129.57 (CH), 128.90 (CH), 128.78 (CH), 128.20 (C), 127.82 (CH), 127.75 (CH), 125.47 (CH), 124.98 (CH), 124.24 (CH), 120.99 (C), 120.76 (CH), 120.60 (CH), 119.58 (C), 113.35 (CH), 111.42 (C), 110.88 (C), 74.21 (CH_2), 73.79 (CH), 68.76 (CH), 61.87 (CH_2), 58.29 (CH), 52.50 (CH), 51.02 (CH_2), 50.86 (CH), 50.23 (C), 50.13 (C), 47.45 (CH), 47.40 (CH), 40.95 (CH), 35.95 (CH_2), 33.33 (CH_2), 32.75 (CH_2), 32.03 (CH_2), 31.28 (CH_2), 31.13 (CH), 30.37 (CH), 29.82 (CH), 28.99-28.80 (CH_2), 28.79 (CH), 28.69 (CH_2), 28.66 (CH_2), 28.50 (CH), 28.41 (CH_2), 28.37 (CH_2), 28.22 (CH_3), 24.44 (CH_2), 24.15 (CH_2), 22.08 (CH_2), 21.73 (CH_2), 21.67 (CH_2), 21.04 (CH), 20.99 (CH), 19.18 (CH_3), 16.41 (CH_3), 13.93 (CH_3), 13.86 (CH_3).

4. MALDI-MS/MS analysis of PC1 and PC2

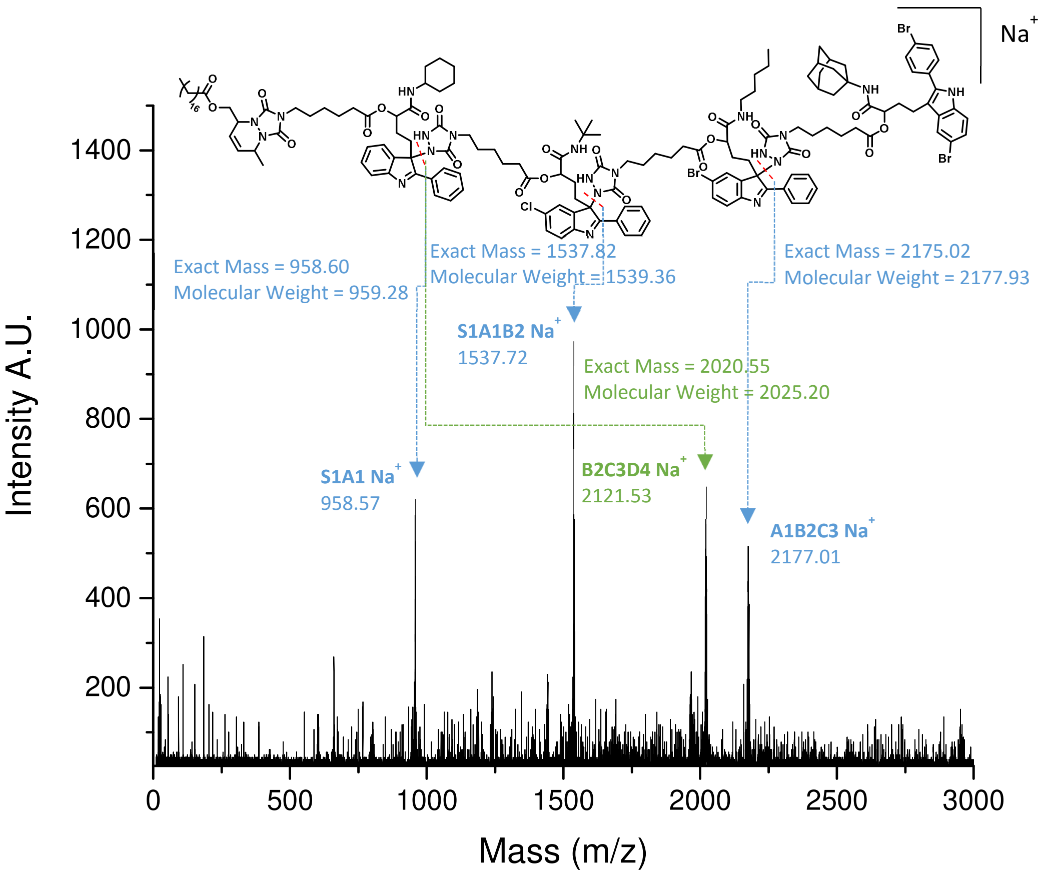

Figure S1. MALDI-MS/MS spectrum of **PC1** from which distinct fragments of the oligomer can be identified and the original order of the sequence can be determined. It should be noted that the molecular ion of the complete oligomer A1B2C3D4 was not detected under the applied conditions.

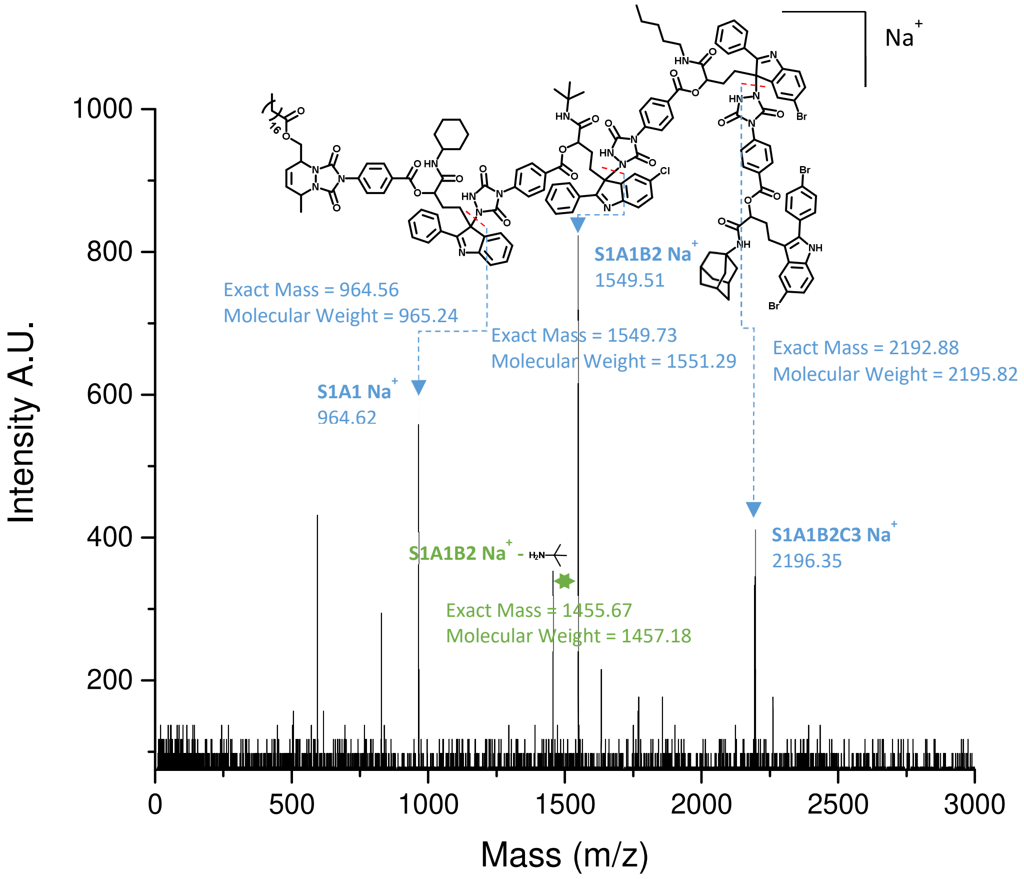

Figure S2. MALDI-MS/MS spectrum of **PC2** from which distinct fragments of the oligomer can be identified and the original order of the sequence can be determined. It should be noted that the molecular ion of the complete oligomer A1B2C3D4 was not detected under the applied conditions.

5. Encryption model study

(i) Aliphatic macromolecular pin code

A solution of **PC1** (58.7 mg, 0.020 mmol, 1.0 equiv.) in DMSO-d_6 (1 mL) was transferred into an NMR tube and placed in a pre-heated oil bath at 150 °C. After heating for 15 minutes, 0.20 mL of the mixture was taken out and diluted with 0.40 mL tetrahydrofuran prior to SEC analysis (see **Figure 3c**).

(ii) Aromatic macromolecular pin code

A solution of **PC2** (59.2 mg, 0.020 mmol, 1.0 equiv.) in DMSO-d_6 (1 mL) was transferred into an NMR tube and placed in a pre-heated oil bath at 120 °C. After heating for 15 minutes, 0.20 mL of the mixture was taken out and diluted with 0.40 mL tetrahydrofuran prior to SEC analysis (see **Figure 3d**).

6. Decryption model study

(i) Aliphatic macromolecular pin code

A solution of **PC1** (58.7 mg, 0.020 mmol, 1.0 equiv.) and sorbic alcohol (6.5 mg, 0.066 mmol, 3.3 equiv.) in 1 mL DMSO-d_6 was transferred into an NMR tube and placed in a pre-heated oil bath at 120 °C, 135 °C or 150 °C. At distinct time intervals, the NMR tube was taken out of the oil bath and cooled immediately under running tap water before being submitted to NMR analysis (see **Figure S3**), from which the conversion of **PC1** could be determined as a function of time (see **Figure S4**).

(ii) Aromatic macromolecular pin code

A solution of **PC2** (59.2 mg, 0.020 mmol, 1.0 equiv.) and sorbic alcohol (6.5 mg, 0.066 mmol, 3.3 equiv.) in 1 mL DMSO-d_6 was transferred into an NMR tube and placed in a pre-heated oil bath at 90 °C, 105 °C or 120 °C. At distinct time intervals, the NMR tube was taken out of the oil bath and cooled immediately under running tap water before being submitted to NMR analysis (see **Figure S5**), from which the conversion of **PC2** could be determined as a function of time (see **Figure S6**).

a)

b)

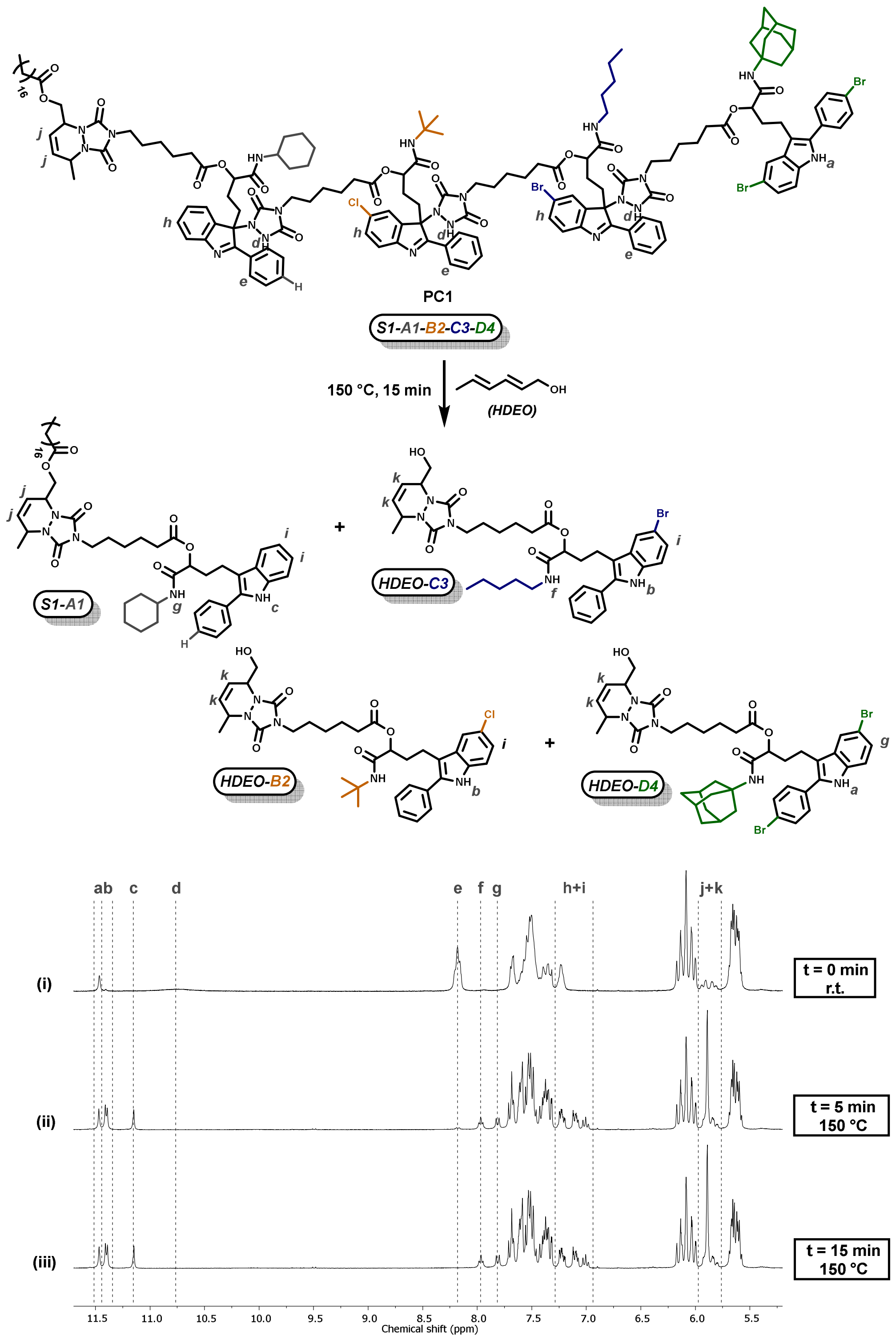

Figure S3. a) Decryption of **PC1** into its separated monomers upon heating in the presence of (*E*,*E*)-2,4-hexadien-1-ol (HDEO) as a trapping agent. b) Zoom of the ^{1}H NMR spectra (DMSO-d_6) before (i), after 5 min (ii) and 15 min (iii) of heating at 150 °C. The spectra show the disappearance of the TAD-indole adduct NH-proton resonances *(c)* and the appearance of the released indole NH-signals *(a)* and *(b)*, indicating the successful cleavage of the macromolecular pin code.

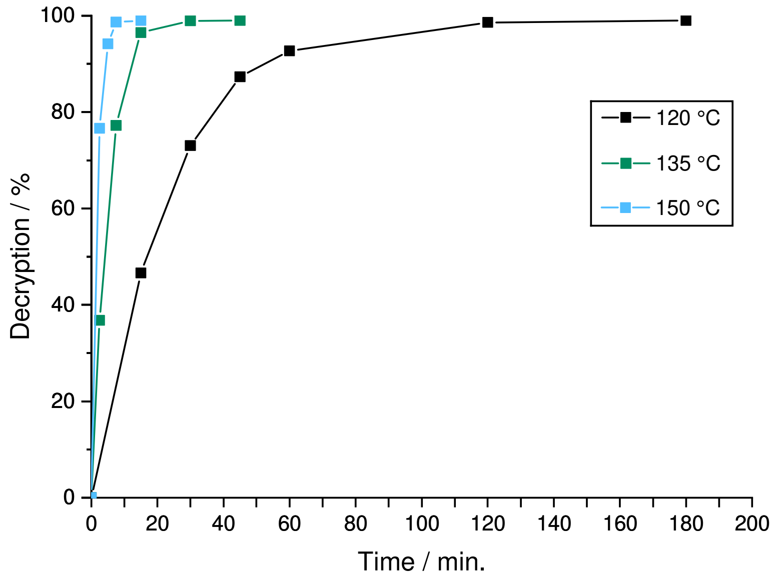

Figure S4. Thermal reversibility study of **PC1** (0.02 M, DMSO-*d*$_6$) to assess the kinetics of the decryption process.

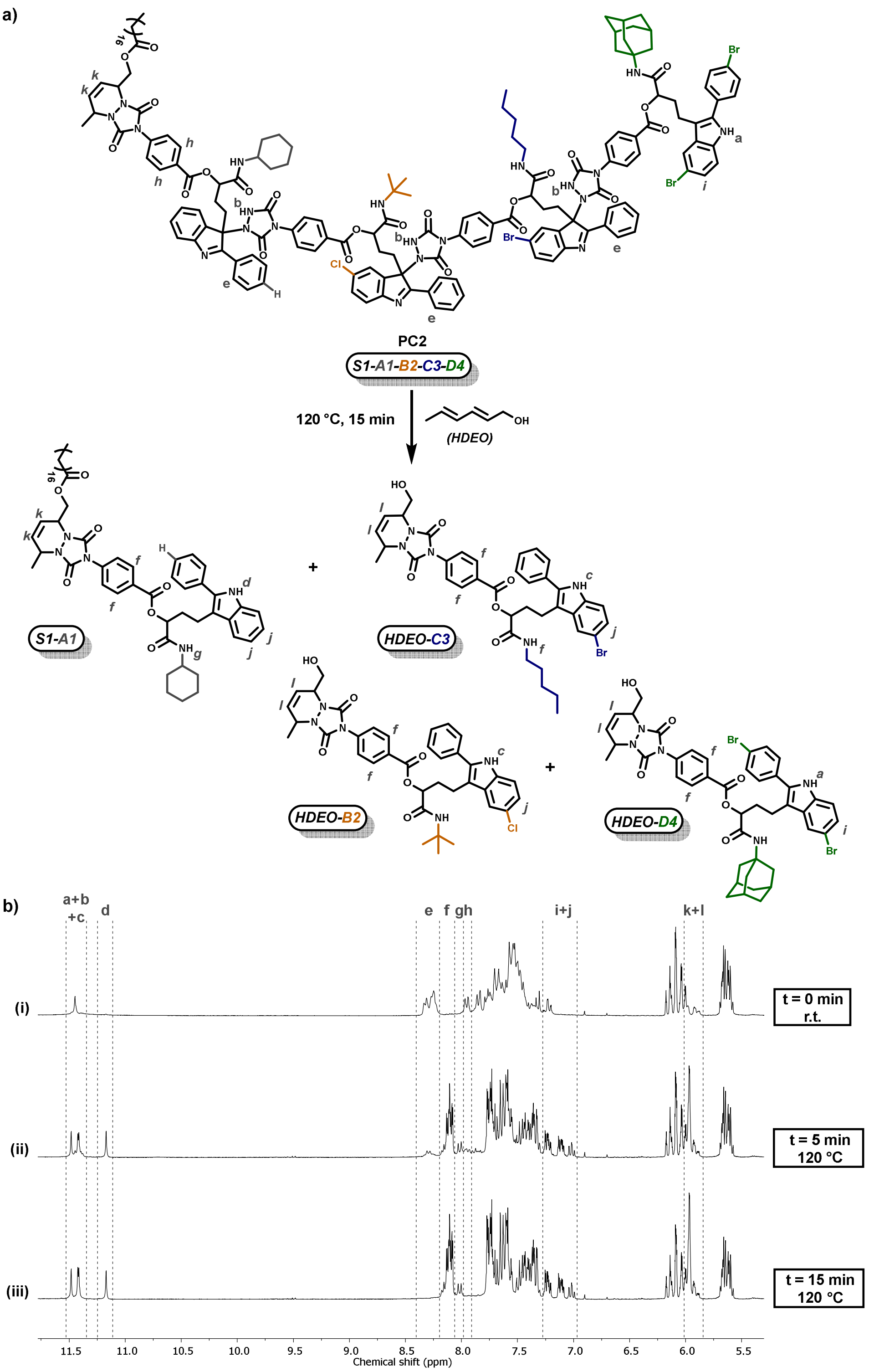

Figure S5. a) Decryption of **PC2** into its separated monomers upon heating in the presence of (*E*,*E*)-2,4-hexadien-1-ol (HDEO) as a trapping agent. b) Zoom of the ^{1}H NMR spectra (DMSO-d_6) before (i), after 5 min (ii) and 15 min (iii) of heating at 150 °C. The spectra show the disappearance of the TAD-indole adduct NH-proton resonances *(c)* and the appearance of the released indole NH-signals *(a)* and *(b)*, indicating the successful cleavage of the macromolecular pin code.

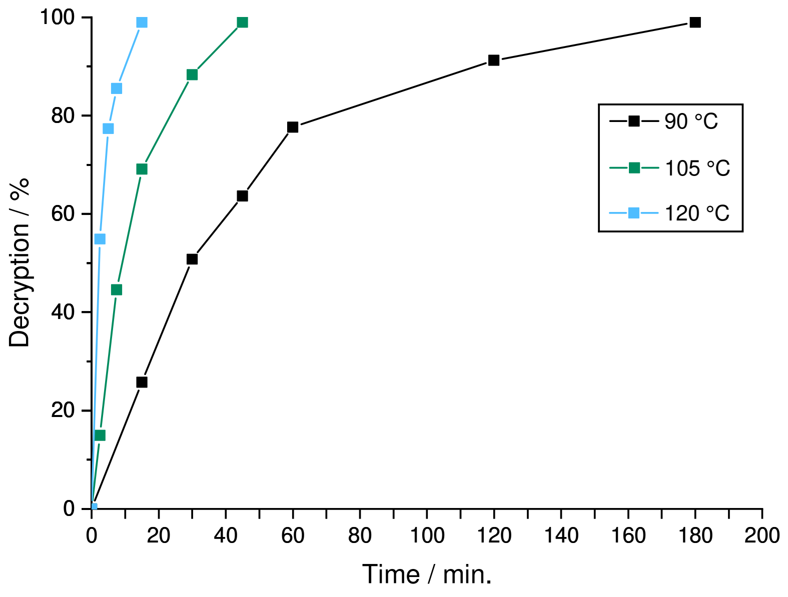

Figure S6. Thermal reversibility study of **PC2** (0.02 M, DMSO-d_6) to assess the kinetics of the decryption process.

The kinetic reversibility profiles of the decryption model studies for both the aliphatic and aromatic pin codes (i.e. **PC1** and **PC2**, respectively) depicted in **Figure S4** and **Figure S6**, respectively, express the fraction of retro-TAD-indole reaction that has occurred upon heating the sequence-defined oligomers at a well-defined temperature for a distinct period of time.

The presence of sorbic alcohol (HDEO) as a kinetic trap for the *in situ* released TAD at elevated temperatures, and the therewith associated fast reaction kinetics with regard to the TAD-indole recombination reaction (i.e. $k_{TAD\text{-}HDEO} > 10^3\ k_{TAD\text{-}indole}$),[3-4] allows for a simplified expression of the observed overall decryption reaction rate coefficient k_{obs}.

$$-\frac{d[A]}{dt} = k_{obs}\,[A]$$

$$\Rightarrow \ln[A]_t - \ln[A]_0 = -\,k_{obs}t$$

$$\Rightarrow \ln[A]_t = -\,k_{obs}t + \ln[A]_0$$

The fraction of TAD-indole adducts remaining at specific time intervals, i.e. $\ln[A]_t$ could be derived from the decryption conversion *via* integration of the respective ^{1}H NMR spectra. The observed rate coefficient k_{obs} was hence determined by a linear regression fit for each of the three temperatures investigated. Using the rearranged Arrhenius equation below, the observed overall activation energy ($E_{a,\ obs}$) for the decryption of the sequence-defined pin codes could eventually be derived from the Arrhenius plot, obtained by a best-fit linear interpolation of ln k_{obs} as a function of T^{-1} (refer to **Table S1**).

$$y = ax + b$$

$$\Rightarrow a = \frac{-E_{a,obs}}{R}$$

$$\Rightarrow E_{a,obs} = -aR$$

With the gas constant R = 8.314 J K^{-1} mol^{-1} and *a* expressed in K, this gives $E_{a,\,obs}$ in J mol^{-1}.

Table S1. Arrhenius plot equations and R^2 values obtained from the kinetic investigations of macromolecular pin codes **PC1** and **PC2**, from which the observed activation energy $E_{a,\,obs}$ and the standard deviation thereof has been determined.

Pin code	Linear regression equation y = a x + b	R^2	$E_{a,\,obs}$ (kJ mol^{-1})	Standard deviation (kJ mol^{-1})
**PC1**	y = -13966 x + 28.40	0.995	116.1	8.5
**PC2**	y = -11514 x + 23.80	0.997	95.7	5.3

Table S2. Purity of oligomers during the synthesis of macromolecular pin codes **PC1** and **PC2** shown in the SEC elugrams in Figure 3a and Figure 3b.

Pin code	Monomer	Dimer	Trimer	Tetramer
**PC1** (Figure 3a)	100 %	100 %	97 %	95 %
**PC2** (Figure 3b)	100 %	98 %	97 %	94 %

7. MALDI-MS/MS analysis of PC1 and PC2 after encryption

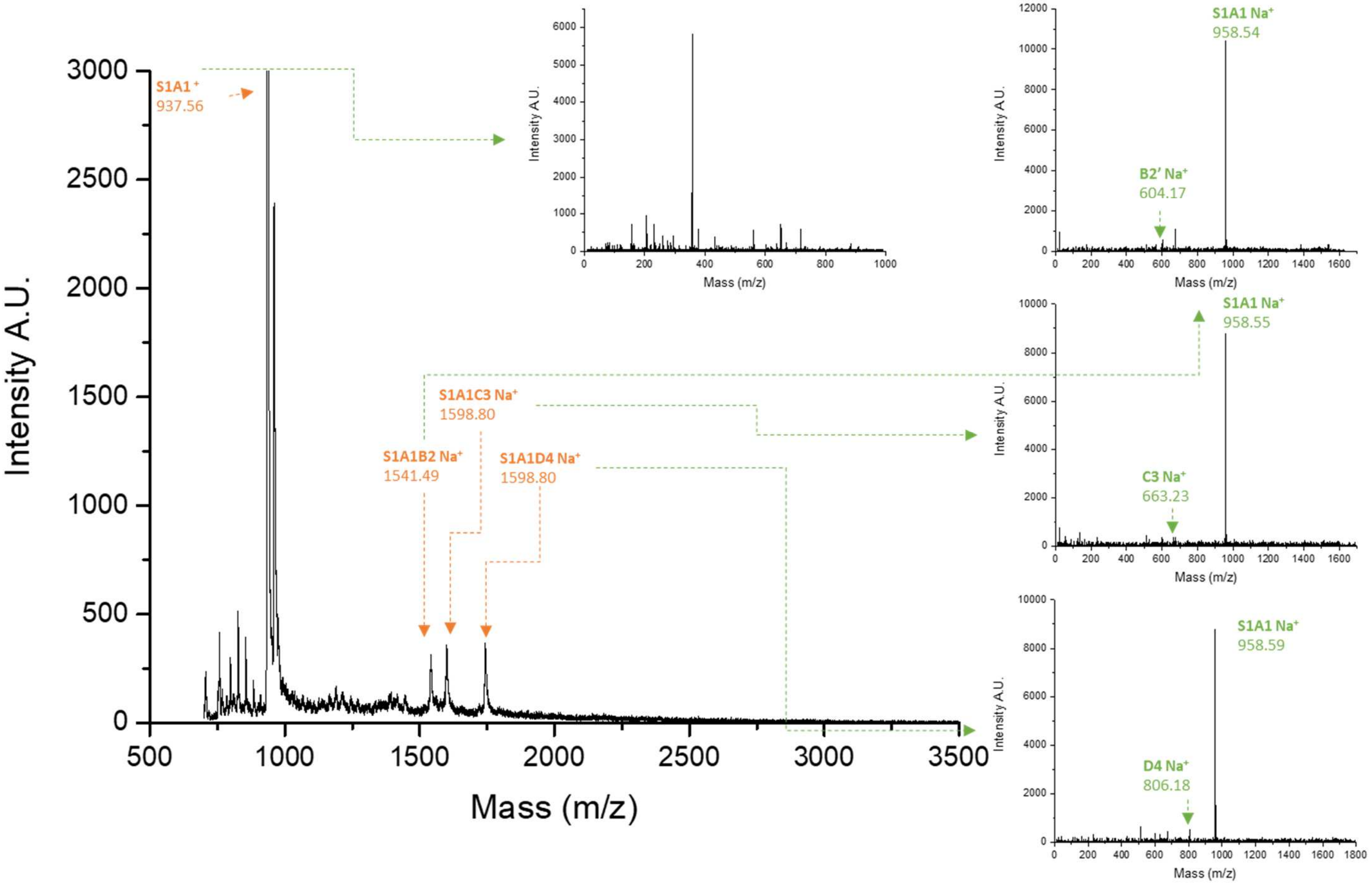

Figure S7. MALDI-MS/MS spectrum of **PC1** after encryption, showing a range of oligomer fragments that do not enable the unambiguous construction of the initially written pin code.

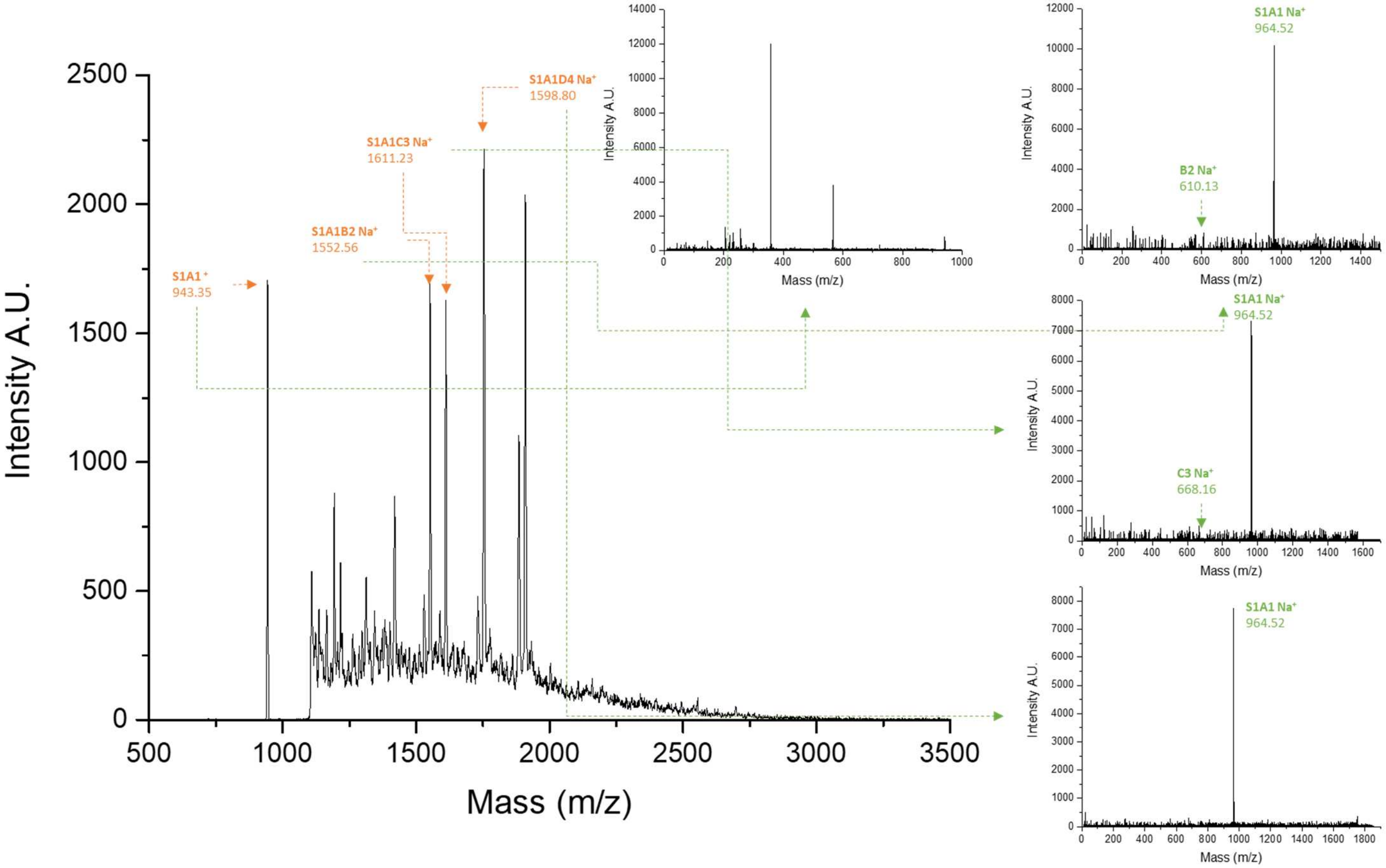

Figure S8. MALDI-MS/MS spectrum of **PC2** after encryption, showing a range of oligomer fragments that do not enable the unambiguous construction of the initially written pin code.

8. ESI-MS analysis of the encryption of PC1 and PC2

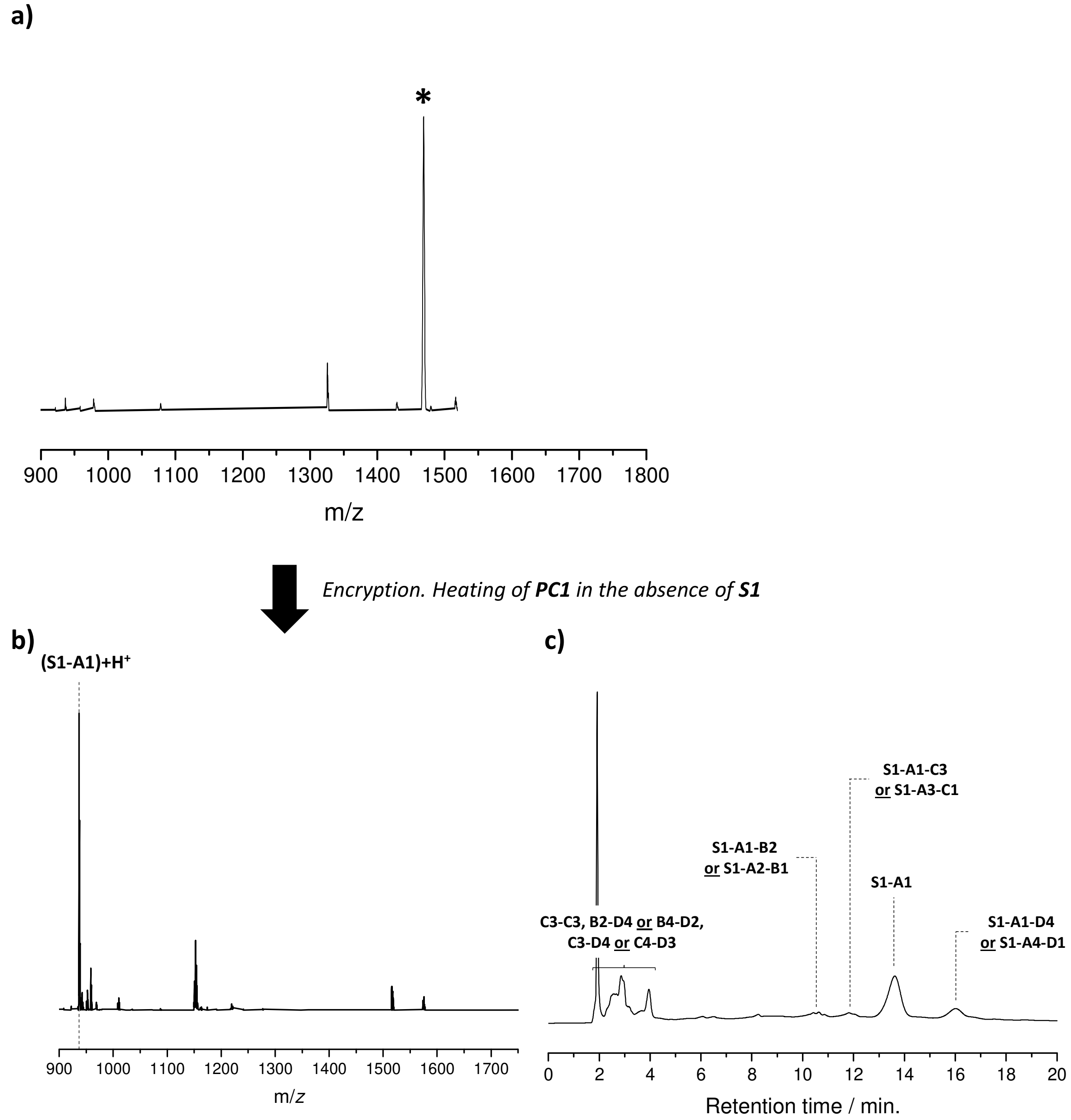

Figure S9. a) ESI-MS spectrum of **PC1** prior to encryption. *denotes [**PC1+2H**]$^{2+}$. b) ESI-MS spectrum of **PC1** following encryption, showing the absence of the expected monomer fragments of **PC1**, except that of A1, which can be identified because of the irreversible Diels-Alder adduct formed with the use of **S1** at the beginning of the sequence. c) LC chromatogram following encryption of **PC1**. Apart from A1, no other isolatable or readable fragments can unambiguously be deciphered. The fragments observed relate to recombined fragments of the macromolecular pin code without the **S1** trapping agent, which are unreadable.

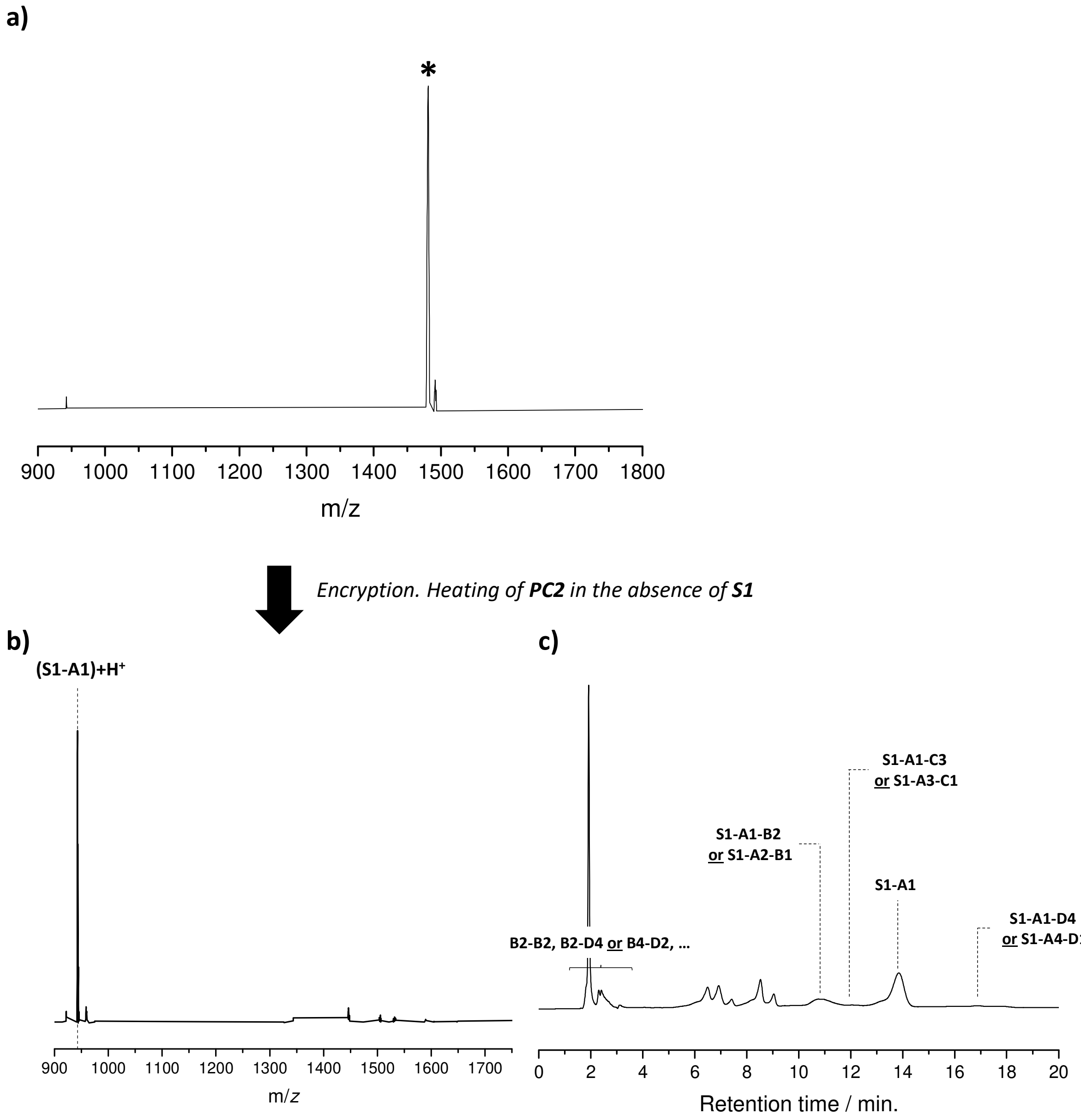

Figure S10. a) ESI-MS spectrum of **PC2** prior to encryption. *denotes [**PC2+2H**]$^{2+}$. b) ESI-MS spectrum of **PC2** following encryption, showing the absence of the expected monomer fragments of **PC2**, except that of A1, which can be identified because of the irreversible Diels-Alder adduct formed with the use of **S1** at the beginning of the sequence. c) LC chromatogram following encryption of **PC2**. Apart from A1, no other isolatable or readable fragments can unambiguously be deciphered. The fragments observed relate to recombined fragments of the macromolecular pin code without the **S1** trapping agent, which are unreadable.

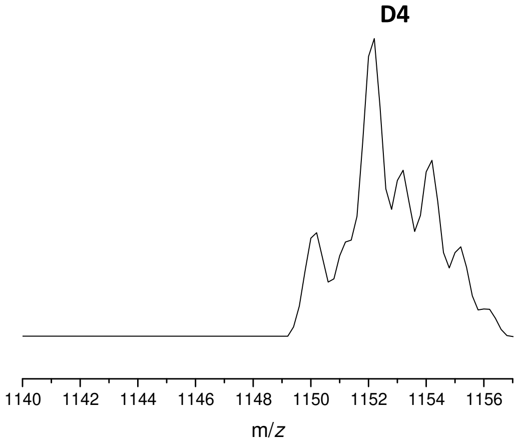

Figure S11. Zoom of the ESI-MS spectrum in **Figure 3f**, showing the isotopic pattern of fragment **D4**.

9. Decryption and read-out of PC1 and PC2

Besides kinetic ^{1}H NMR measurements of the decryption process, the thermal dissociation of the sequence-defined macromolecular pin codes was also monitored by means of LCMS analysis. For this, an anhydrous butyl acetate (1 mL) solution of **PC1** (58.7 mg, 0.020 mmol, 1.0 equiv.) or **PC2** (59.2 mg, 0.020 mmol, 1.0 equiv.) in the presence of conjugated diene **S1** (24.1 mg, 0.066 mmol, 3.3 equiv.) was heated at 120 °C for 5 hours or 2 hours, respectively. Subsequently, the solvent was removed *in vacuo* and the resulting residue was re-dissolved in either acetonitrile or tetrahydrofuran and subjected to LC-ESI-MS analysis (see **Figure S12** and **Figure S13**). The different monomer fragments could be readily identified by means of the identified mass fragments from the ESI-MS spectrum, whereby the initial order of the fragments within the sequence could be derived from the distinct isotopic pattern (as illustrated for **PC1** in **Figure S14**).

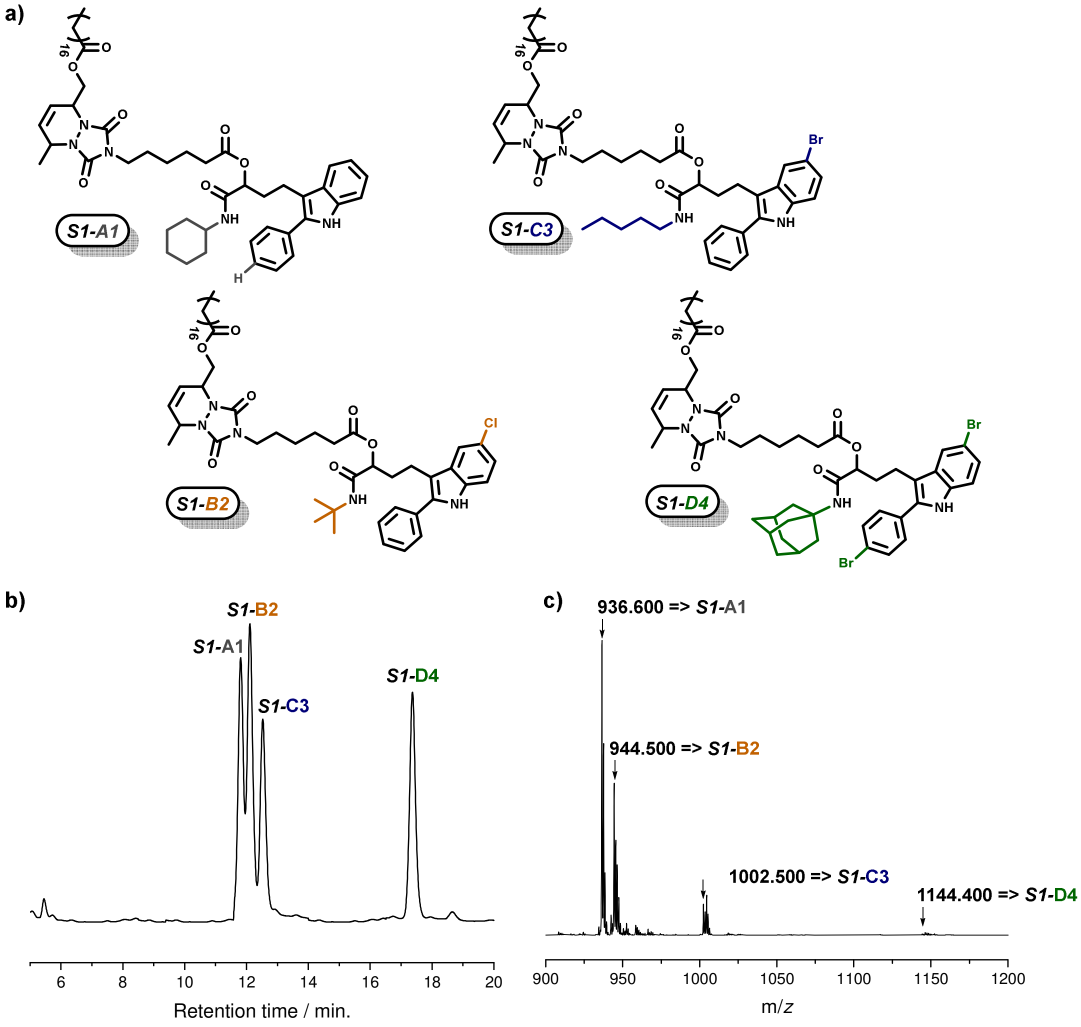

Figure S12. a) Structures of the isolated monomers obtained after decryption of **PC1** upon heating for 5 hours at 120 °C in the presence of **S1**. b) LC chromatogram (λ = 214 nm), indicating the successful separation of the four different monomer units. c) ESI-MS spectrum following the decryption process allows for the mass of the four monomer units to be identified. The monomers can be related to their respective structure based on the differences in mass, while using the isotopic pattern of the indole markers, their original order in the sequence can also be determined.

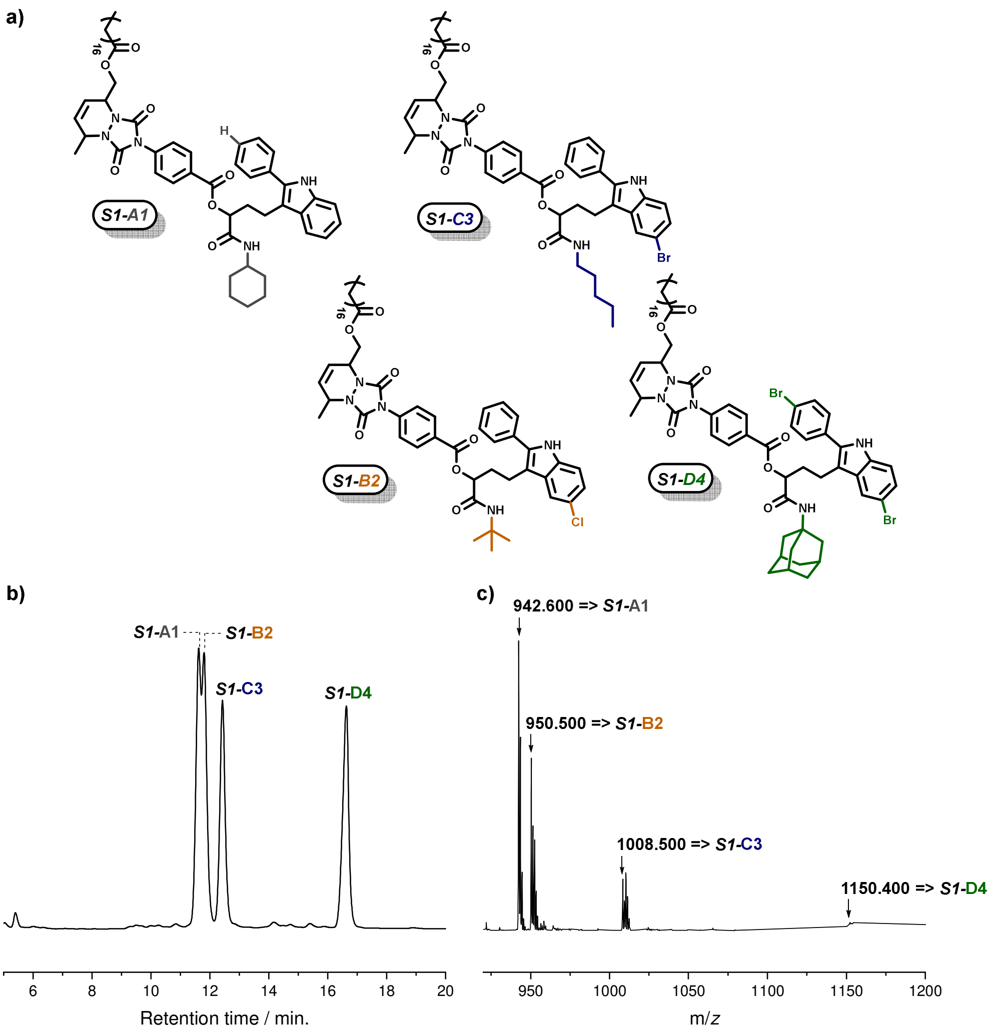

Figure S13. a) Structures of the isolated monomers obtained after decryption of **PC2** upon heating for 5 hours at 120 °C in the presence of **S1**. b) LC chromatogram (λ = 214 nm), indicating the successful separation of the four different monomer units. c) ESI-MS spectrum following the decryption process allows for the mass of the four monomer units to be identified. The monomers can be related to their respective structure based on the differences in mass, while using the isotopic pattern of the indole markers, their original order in the sequence can also be determined.

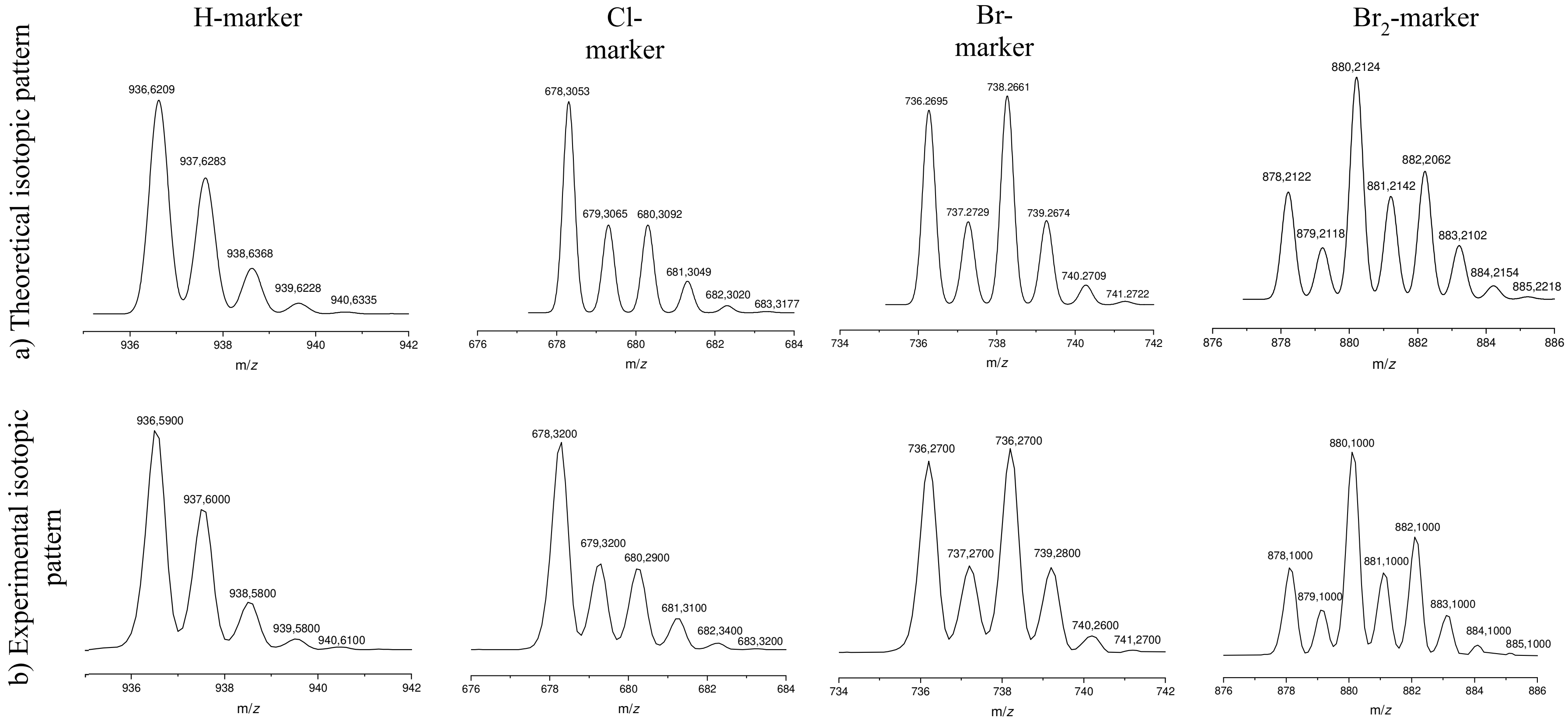

Figure S14. Comparison of the theoretical (a) and the experimentally obtained isotopic pattern (b) of the four separated monomers obtained after decryption of the sequence-defined pin code **PC1** with sorbic alcohol (HDEO), which enabled the straightforward identification of the appropriate indole marker compound.

10. Proof-of-concept demonstration on polymer banknotes

A 2016 series Bank of England £5 note and a 2016 series Reserve Bank of Australia $5 note were first individually suspended in stirring ethanol overnight to verify that the banknotes did not dissolve or discolor in the solvent (see **Figure S15**). Upon removal of the ethanol *in vacuo*, the resulting residue was submitted for ESI-MS analysis whereby a background spectrum was collected (see **Figure S16**).

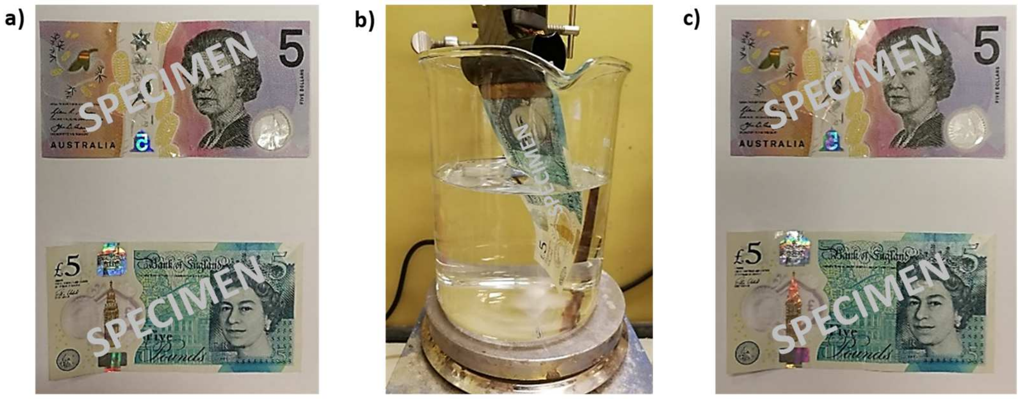

Figure S15. Macromolecular tagging of banknotes for anti-counterfeiting. a) Original $5 and £5 banknotes prior to coating with encrypted pin codes **PC1** and **PC2**, respectively. b) Suspension of the banknotes in ethanol to test that they did not dissolve or discolor in the solvent (here shown for the £5). c) Banknotes after suspension in ethanol and prior to coating, showing that they remained unchanged by the solvent.

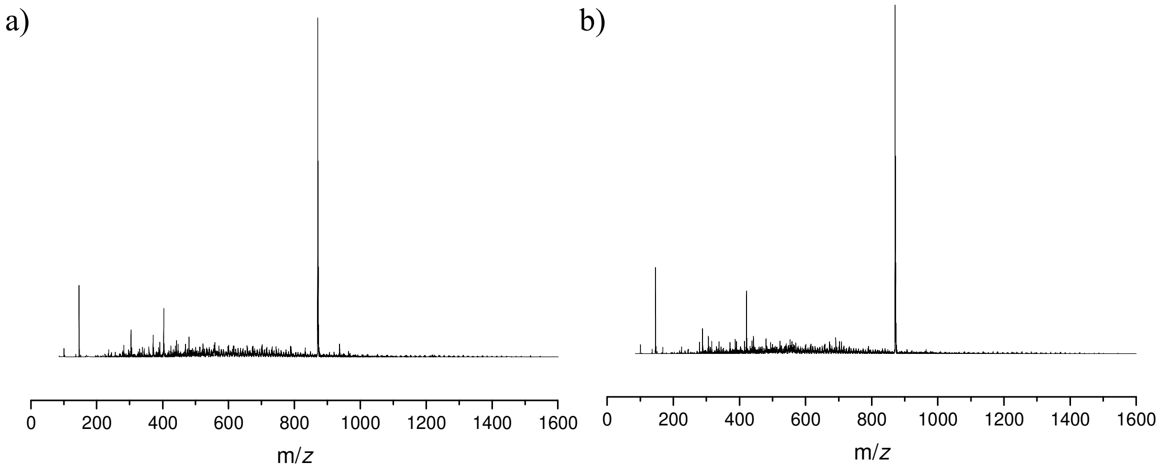

Figure S16. ESI-MS background spectrum of the £5 banknote (a) and $5 banknote (b) collected after overnight suspension in ethanol at room temperature.

Following the blank experiment, the sequence-defined macromolecular pin codes were next applied to the surface of the polymer banknotes. Thus, **PC1** (7.64 mg, 2.6 µmol) and **PC2** (9.67 mg, 3.3 µmol) were each dissolved in anhydrous butyl acetate (1 mL) and encrypted by heating for 2 hours at 120 °C. The solvent was then removed *in vacuo* and the residues were re-dissolved in ethanol (2 mL) and then were deposited on the surface of either the £5 or $5 banknotes, respectively. The solvent was evaporated by gentle heating and the chemically labelled banknotes were left to dry overnight. The encrypted macromolecular pin codes were later extracted from the surface of the banknote by suspending it in

stirring ethanol for 5 hours and the solvent was removed *in vacuo*. A solution of conjugated diene **S1** (3.13 mg, 8.6 µmol) in butyl acetate (1.5 mL) was added to the extracted **PC1**, while a solution of conjugated diene **S1** (3.93 mg, 10.8 µmol) in butyl acetate (1.5 mL) was added to the extracted **PC2**. The two solutions were finally heated for 4 hours at 120 °C, followed by quick cooling under running tap water and solvent removal *in vacuo* and the resulting decrypted macromolecular pin codes were submitted for ESI-MS analysis (**Figure S17** and **Figure S18**).

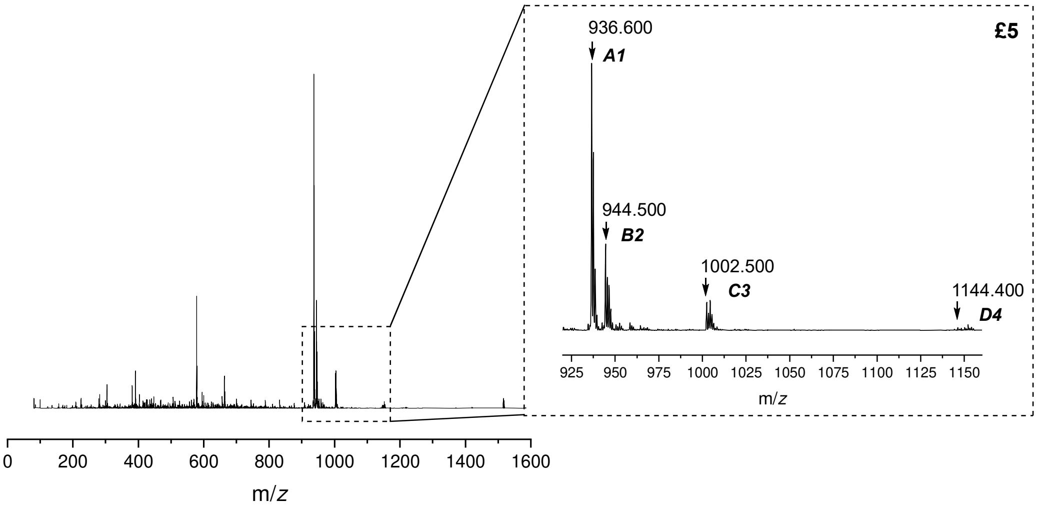

Figure S17. ESI-MS spectrum of the decrypted macromolecular pin code **PC1** extracted from the £5 banknote.

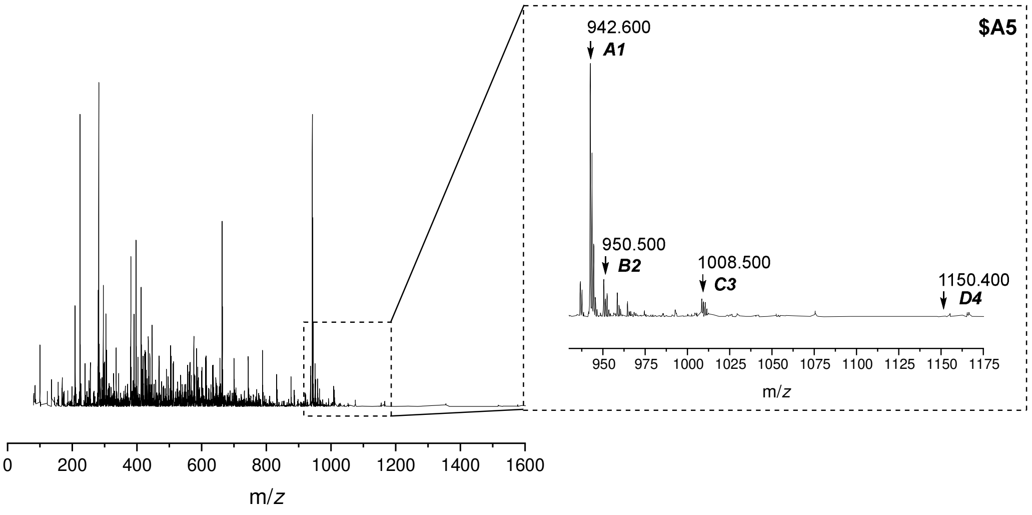

Figure S18. ESI-MS spectrum of the decrypted macromolecular pin code **PC2** extracted from the $5 banknote.

11. Supplementary LCMS Figures

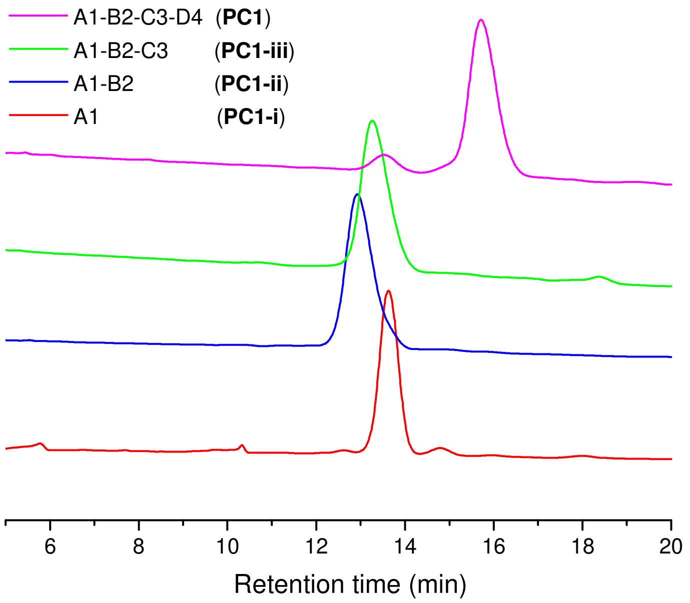

Figure S19. Stacked LCMS chromatograms (λ = 214 nm) recorded during the synthesis of **PC1**.

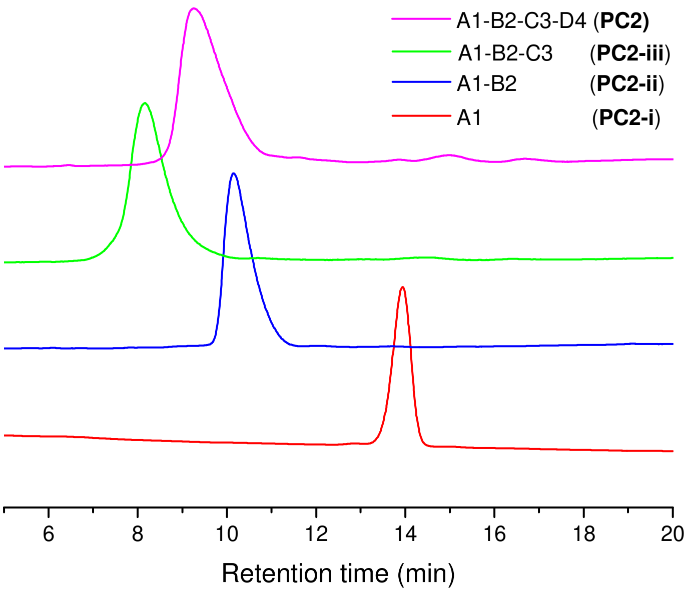

Figure S20. Stacked LCMS chromatograms (λ = 214 nm) recorded during the synthesis of **PC2**.

12. Supplementary NMR Figures

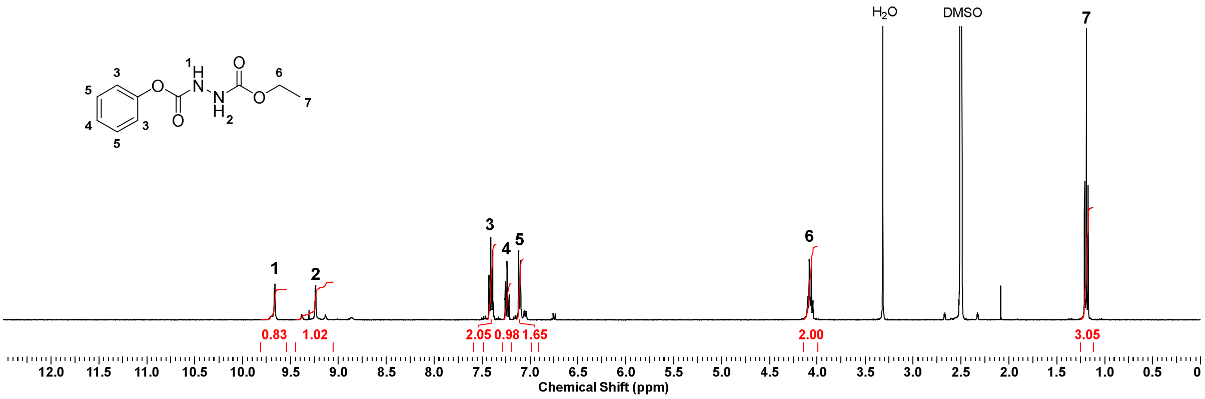

Figure S21. ^{1}H NMR spectrum (DMSO-d_6) of 1-ethyl 2-phenyl hydrazine-1,2-dicarboxylate.

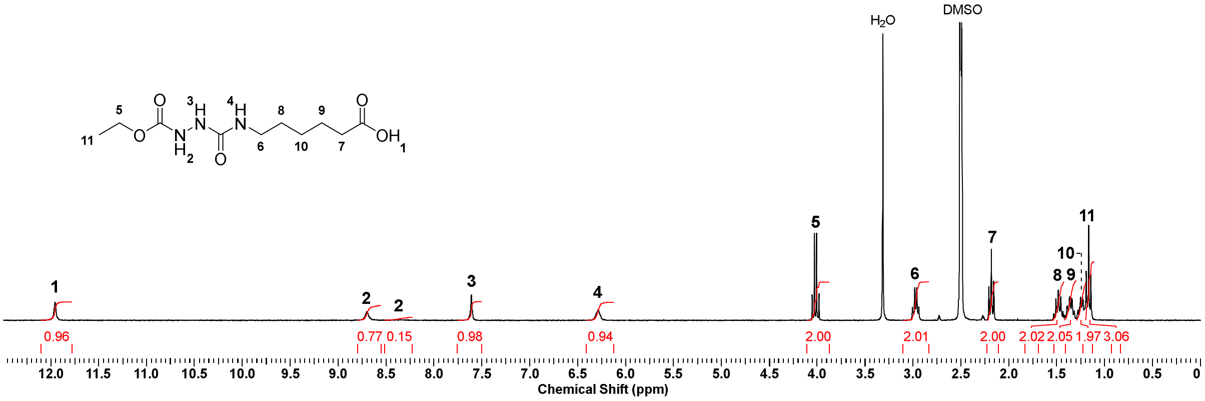

Figure S22. ^{1}H NMR spectrum (DMSO-d_6) of 6-(2-(ethoxycarbonyl)hydrazine-1-carboxamido)hexanoic acid.

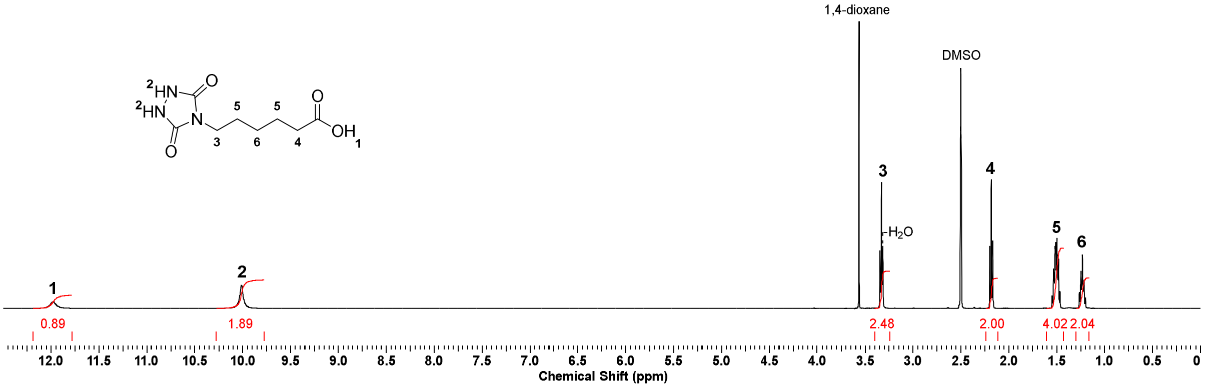

Figure S23. ^{1}H NMR spectrum (DMSO-d_6) of 6-(3,5-dioxo-1,2,4-triazolidin-4-yl)hexanoic acid.

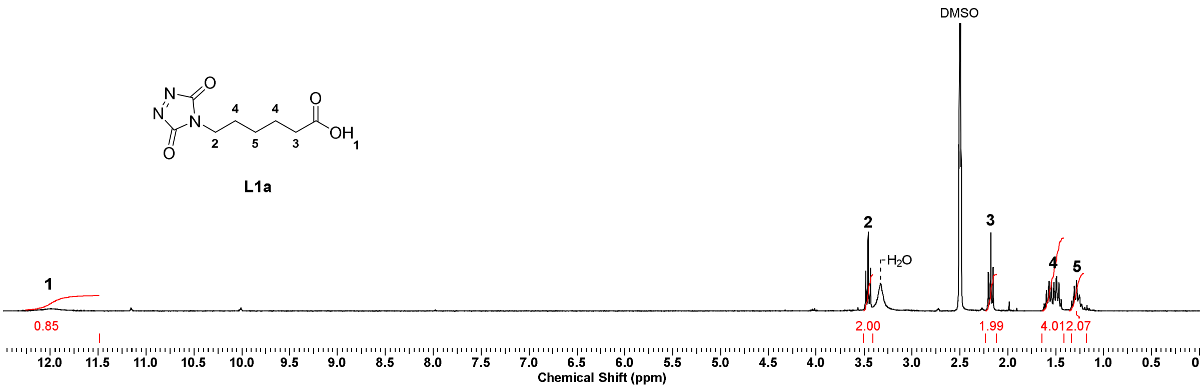

Figure S24. ^{1}H NMR spectrum (DMSO-d_6) of 6-(3,5-dioxo-4H-1,2,4-triazol-4-yl)hexanoic acid **L1a**.

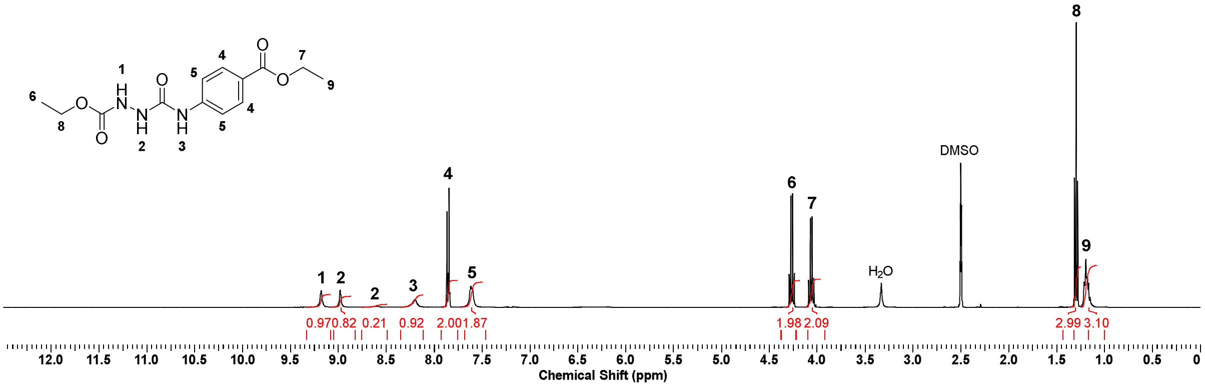

Figure S25. ^{1}H NMR spectrum (DMSO-d_6) of ethyl 2-((4-(ethoxycarbonyl)phenyl)carbamoyl)hydrazine-1-carboxylate.

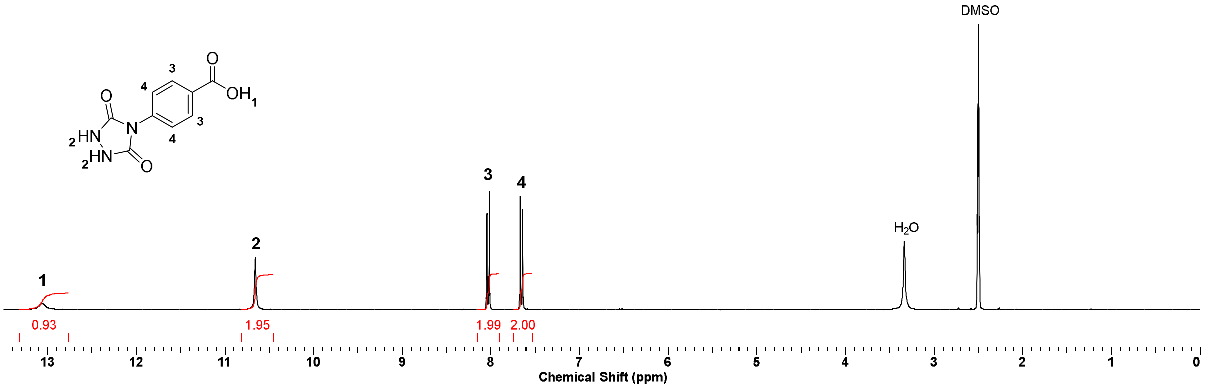

Figure S26. ^{1}H NMR spectrum (DMSO-d_6) of 4-(3,5-dioxo-1,2,4-triazolidin-4-yl)benzoic acid.

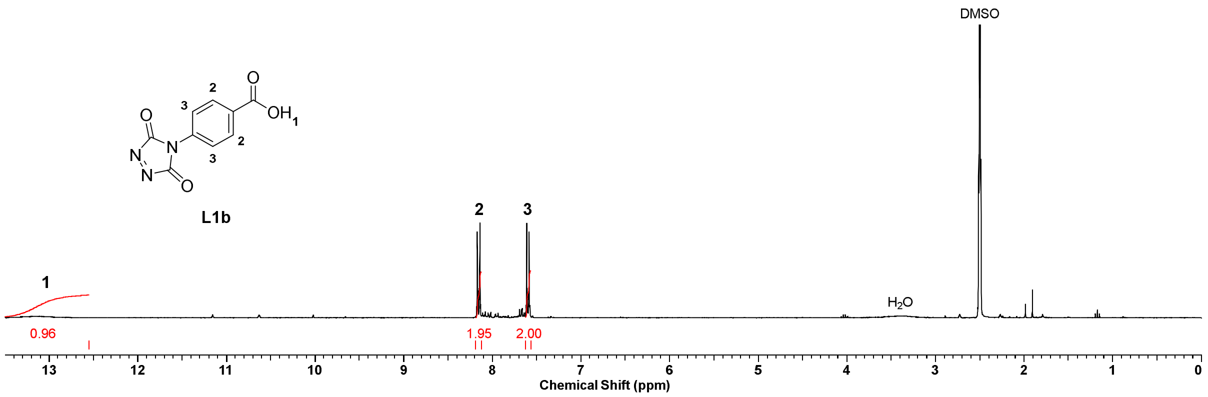

Figure S27. ^{1}H NMR spectrum (DMSO-*d*$_6$) of 4-(3,5-dioxo-3,5-dihydro-4*H*-1,2,4-triazol-4-yl)benzoic acid **L1b**.

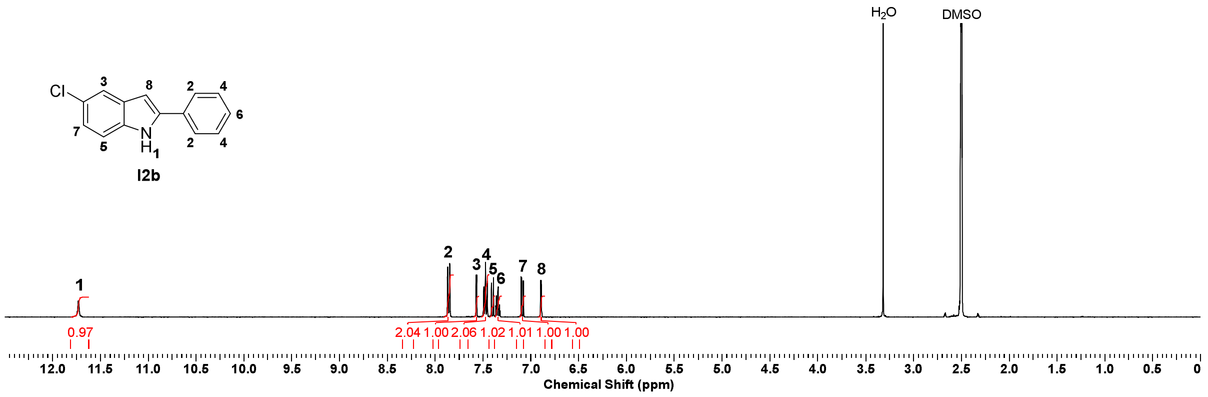

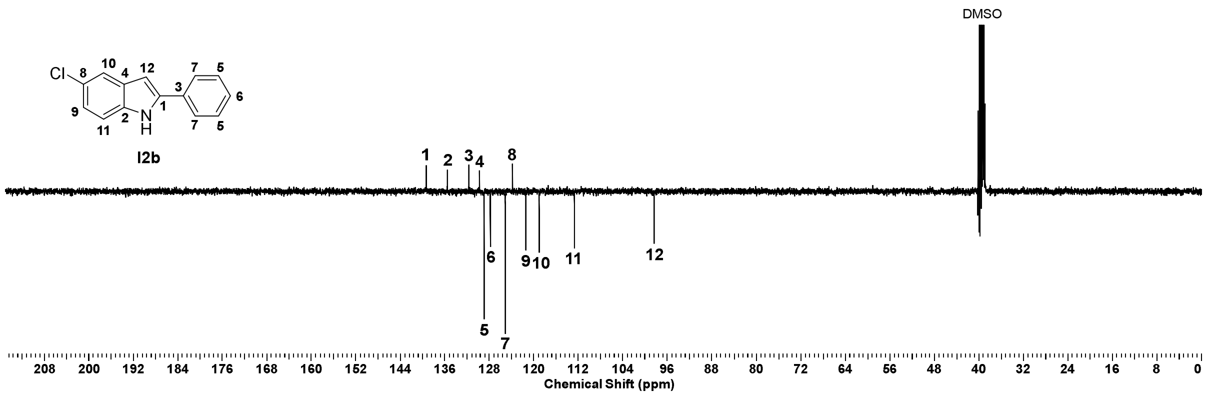

Figure S28. ^{1}H NMR (top) and ^{13}C NMR (bottom) spectrum (DMSO-*d*$_6$) of 5-chloro-2-phenyl-1*H*-indole **I2b**.

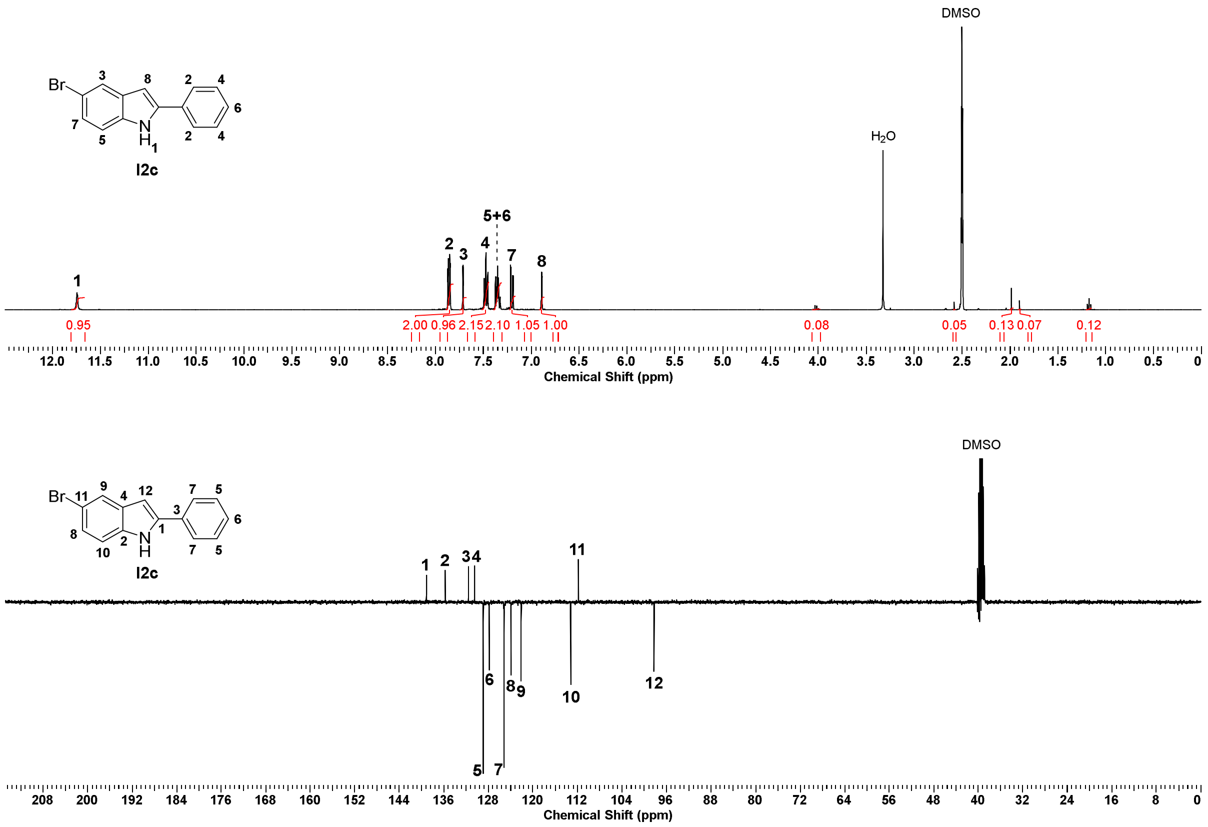

Figure S29. ^{1}H NMR (top) and ^{13}C NMR (bottom) spectrum (DMSO-*d*$_6$) of 5-bromo-2-phenyl-1*H*-indole **I2c**.

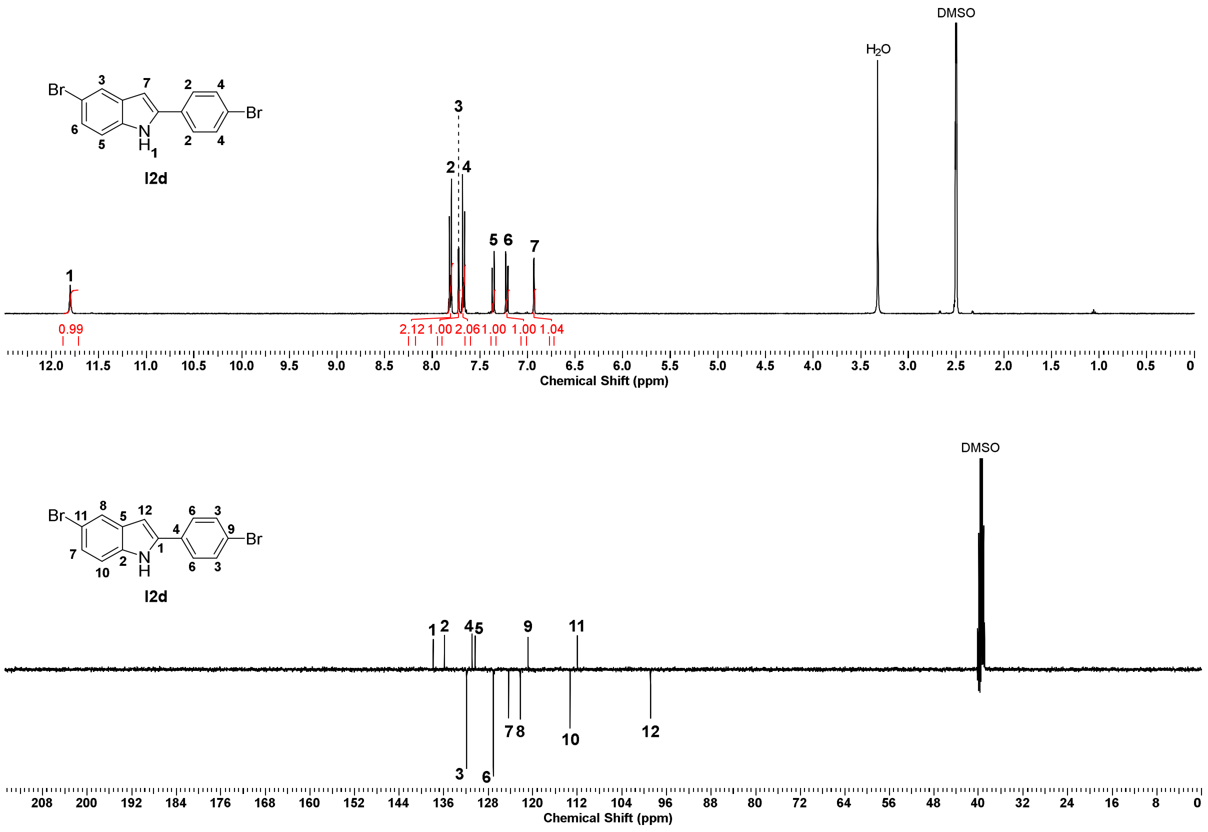

Figure S30. ^{1}H NMR (top) and ^{13}C NMR (bottom) spectrum (DMSO-*d*$_6$) of 5-bromo-2-(4-bromophenyl)-1*H*-indole **I2d**.

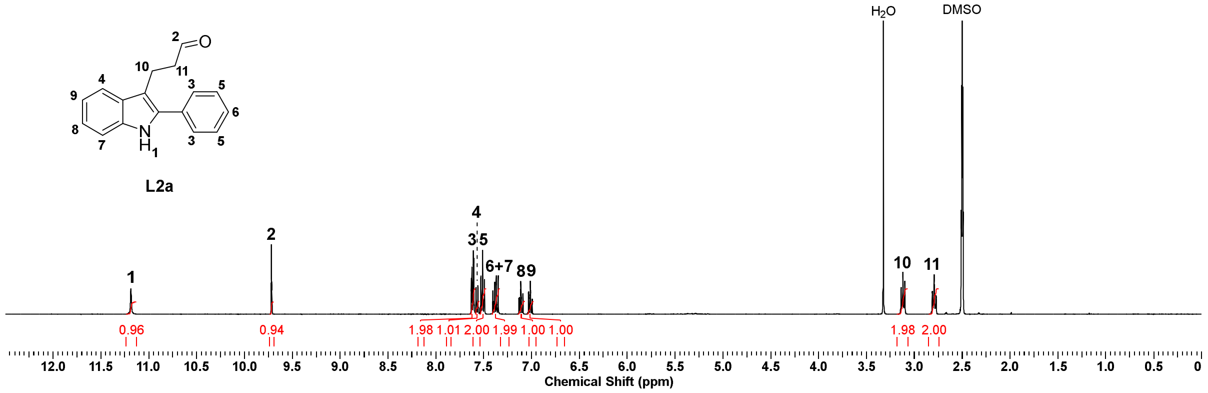

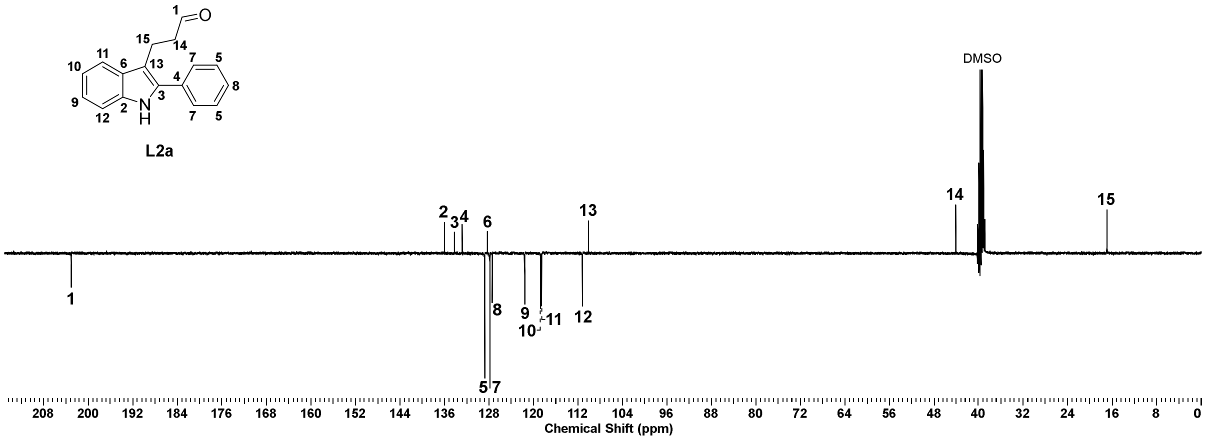

Figure S31. ^{1}H NMR (top) and ^{13}C NMR (bottom) spectrum (DMSO-d_6) of 3-(2-phenyl-1*H*-indol-3-yl)propanal **L2a**.

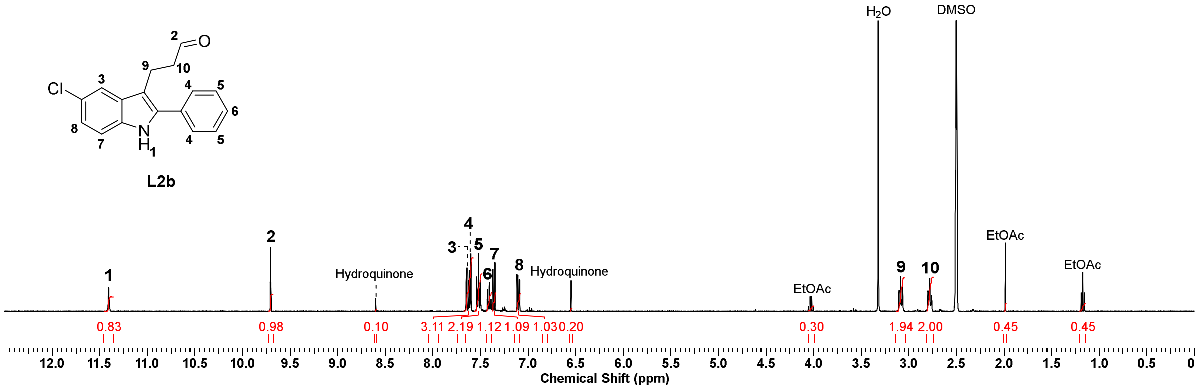

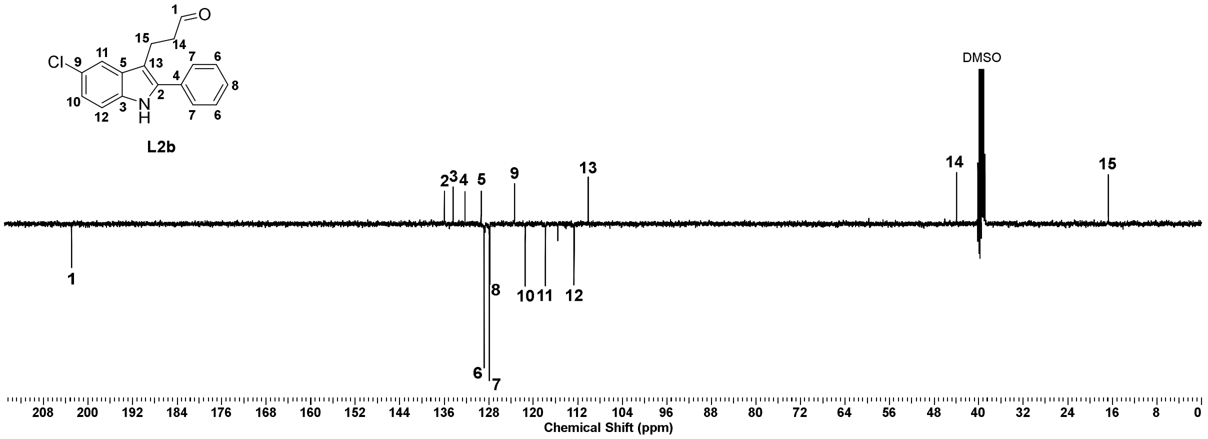

Figure S32. ^{1}H NMR (top) and ^{13}C NMR (bottom) spectrum (DMSO-d_6) of 3-(5-chloro-2-phenyl-1*H*-indol-3-yl)propanal **L2b**.

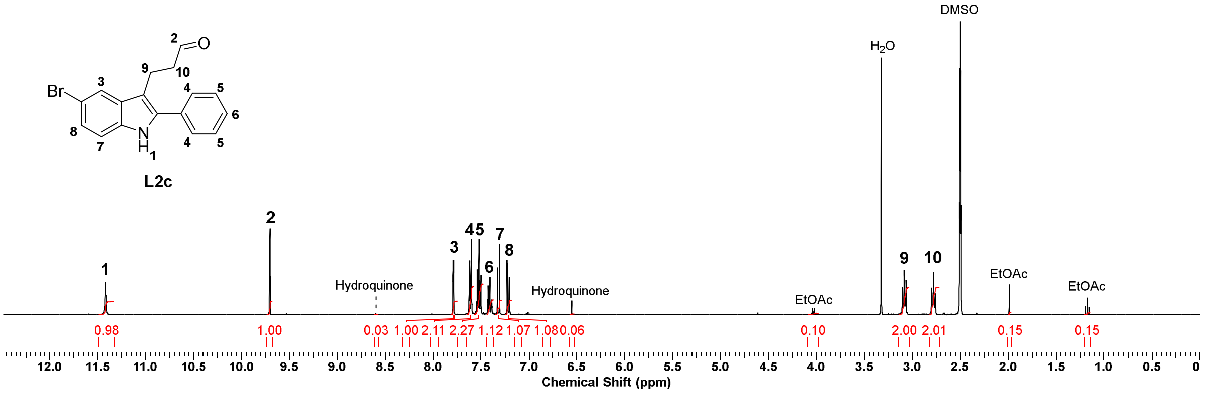

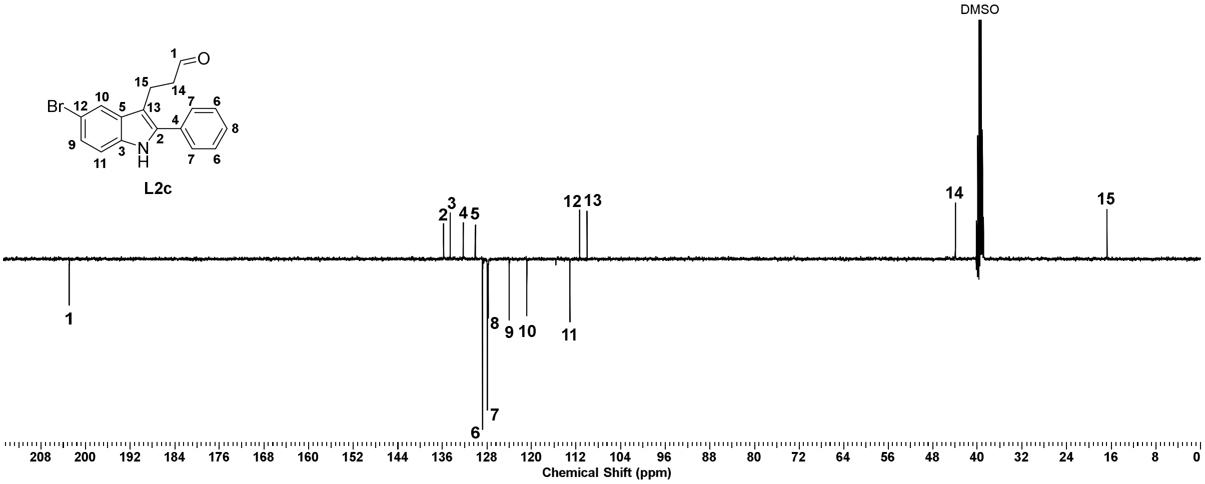

Figure S33. ^{1}H NMR (top) and ^{13}C NMR (bottom) spectrum (DMSO-d_6) of 3-(5-bromo-2-phenyl-1*H*-indol-3-yl)propanal **L2c**.

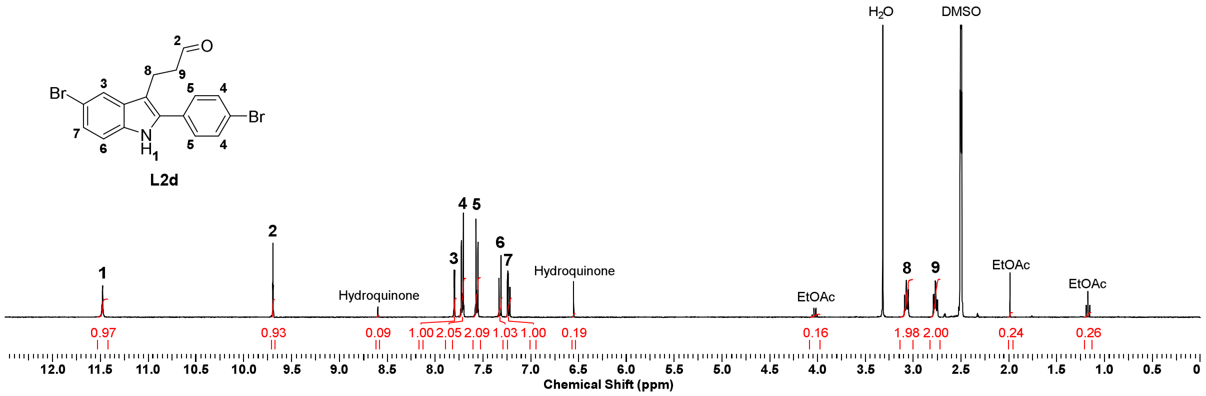

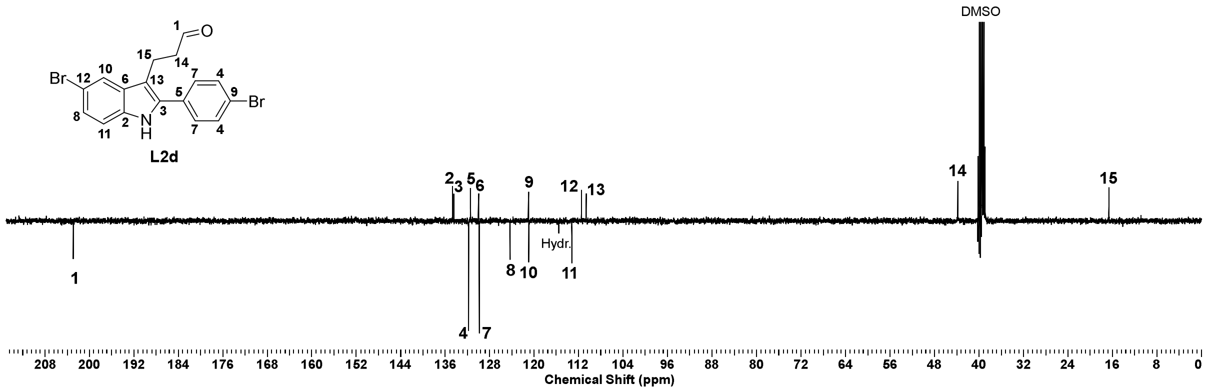

Figure S34. ^{1}H NMR (top) and ^{13}C NMR (bottom) spectrum (DMSO-*d*$_6$) of 3-(5-bromo-2-(4-bromophenyl)-1*H*-indol-3-yl)propanal **L2d**.

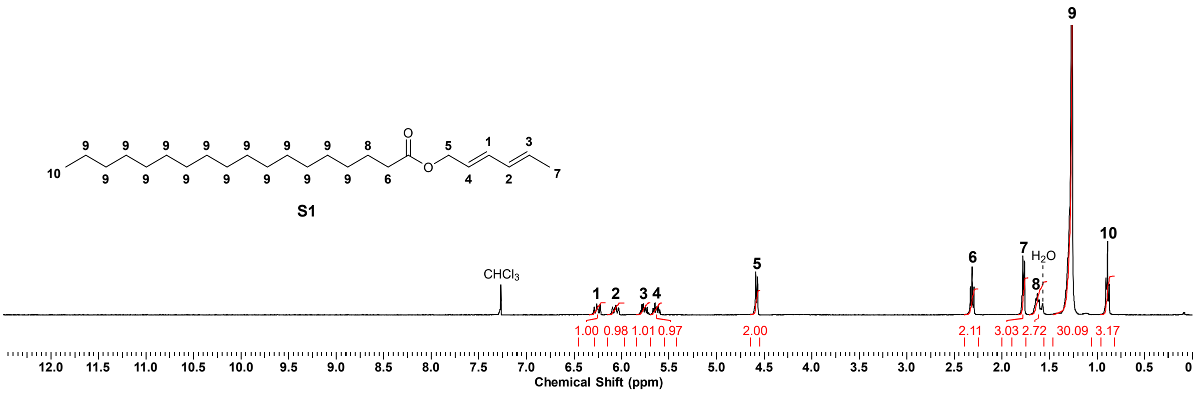

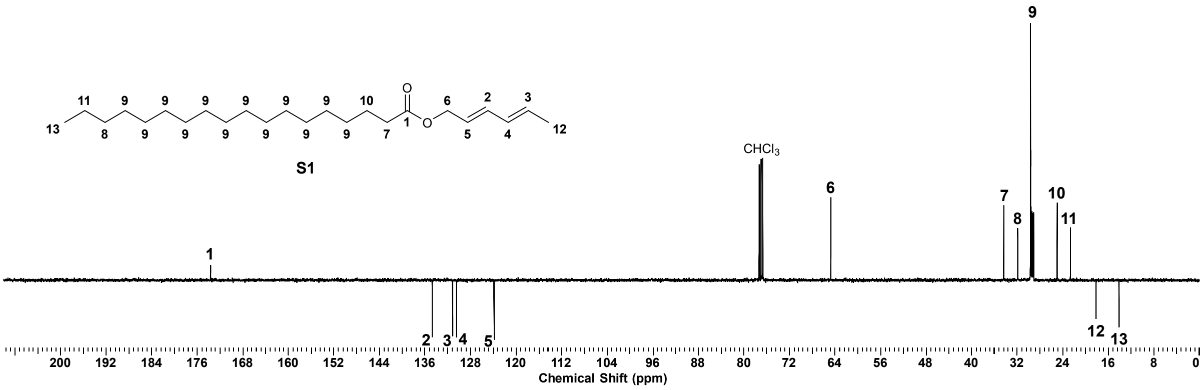

Figure S35. ^{1}H NMR (top) and ^{13}C NMR (bottom) spectrum ($CDCl_3$) of (2E,4E)-hexa-2,4-dien-1-yl stearate **S1**.

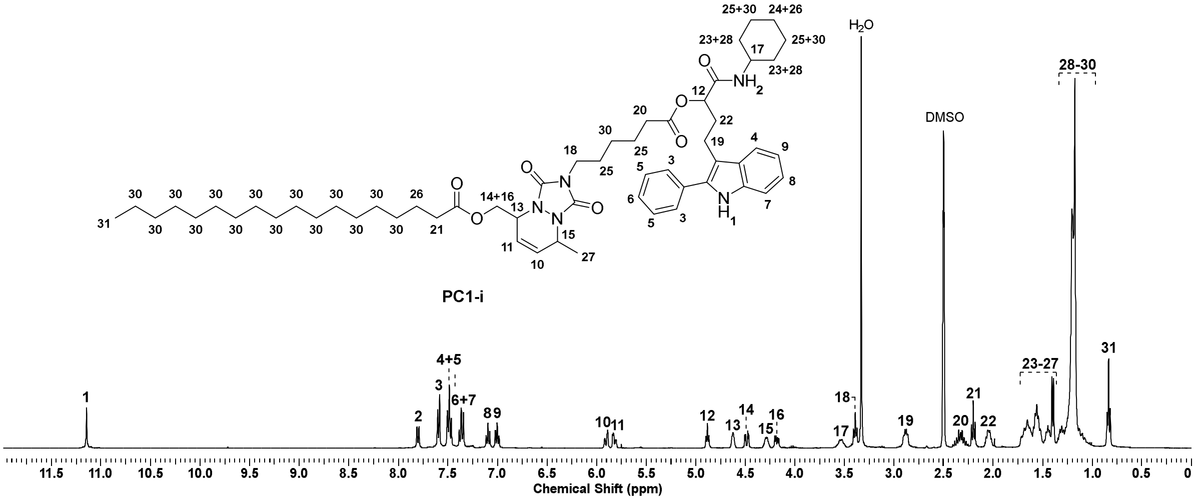

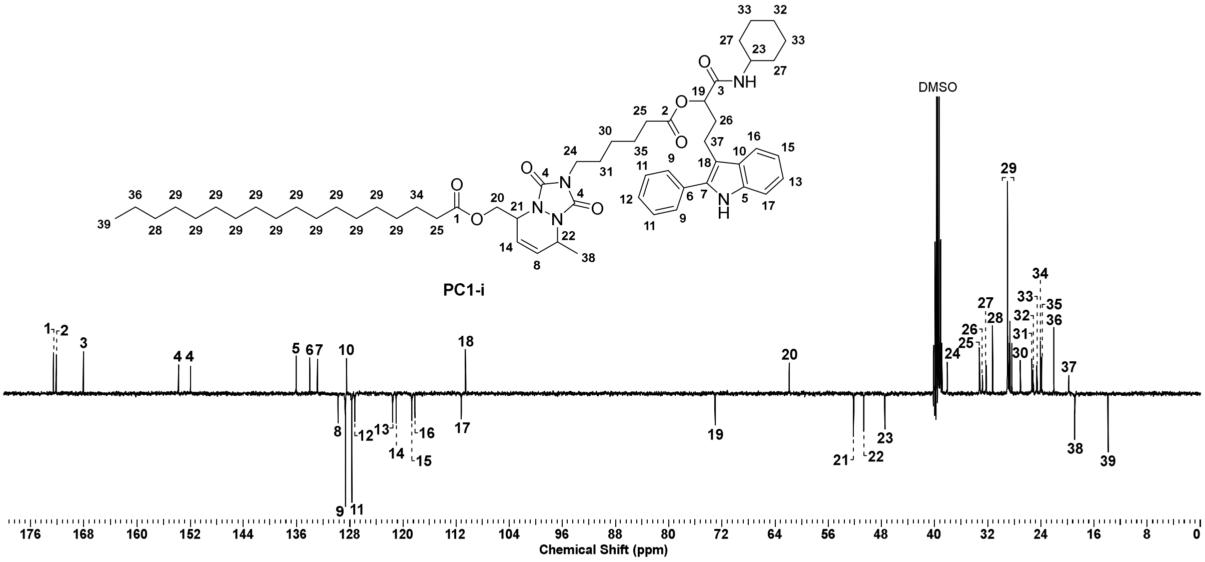

Figure S36. ^{1}H NMR (top) and ^{13}C NMR (bottom) spectrum (DMSO-d_6) of **PC1-i**.

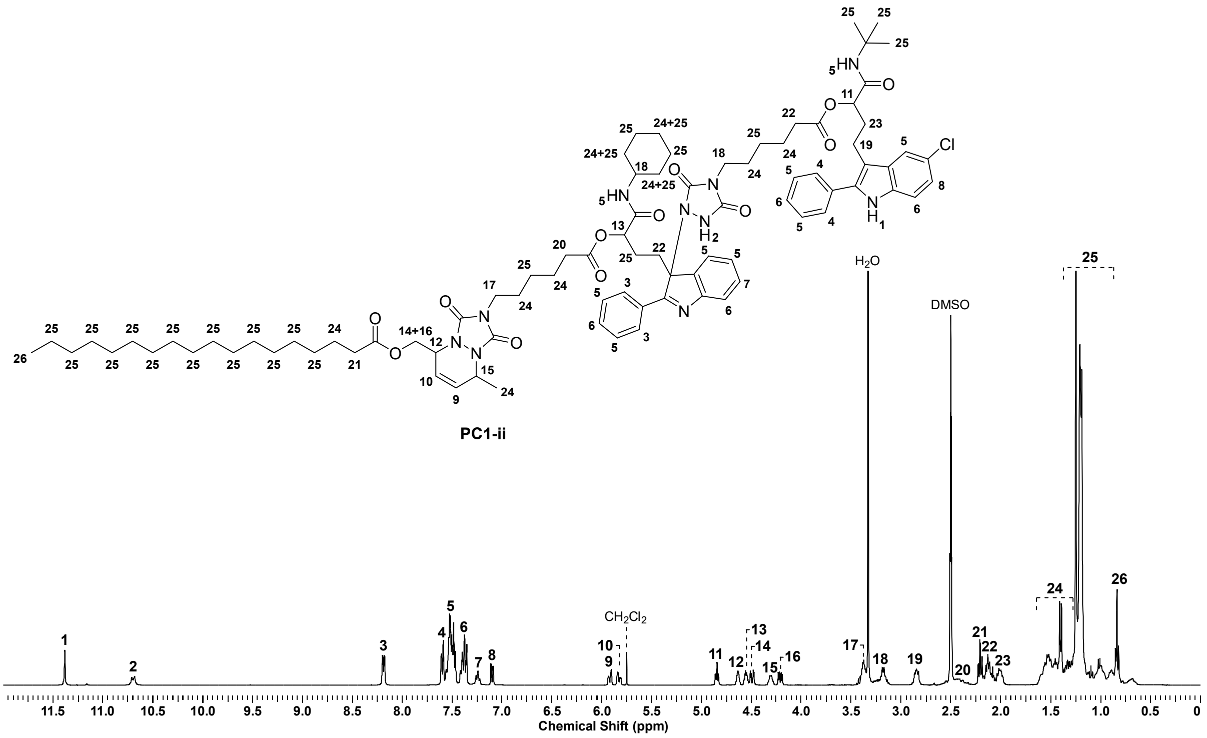

Figure S37. ^{1}H NMR spectrum (DMSO-d_6) of **PC1-ii**.

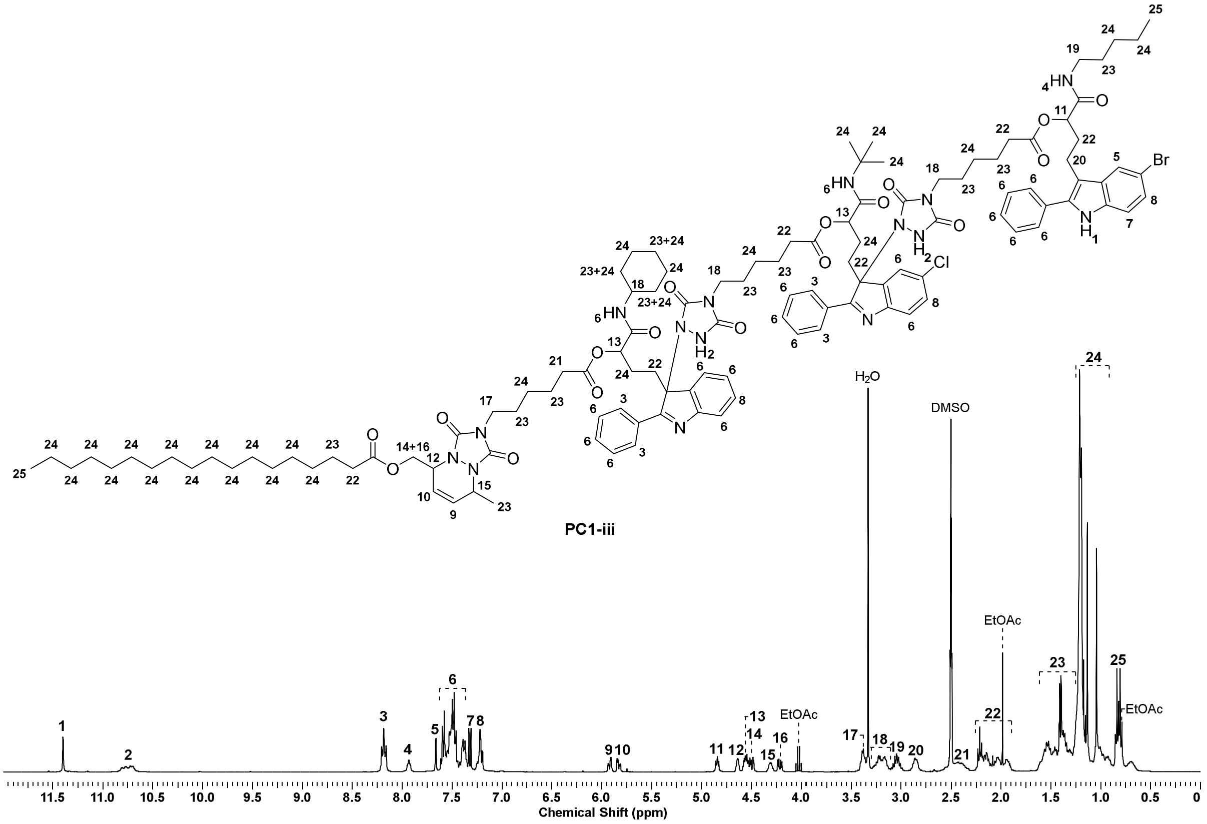

Figure S38. ^{1}H NMR spectrum (DMSO-d_6) of **PC1-iii**.

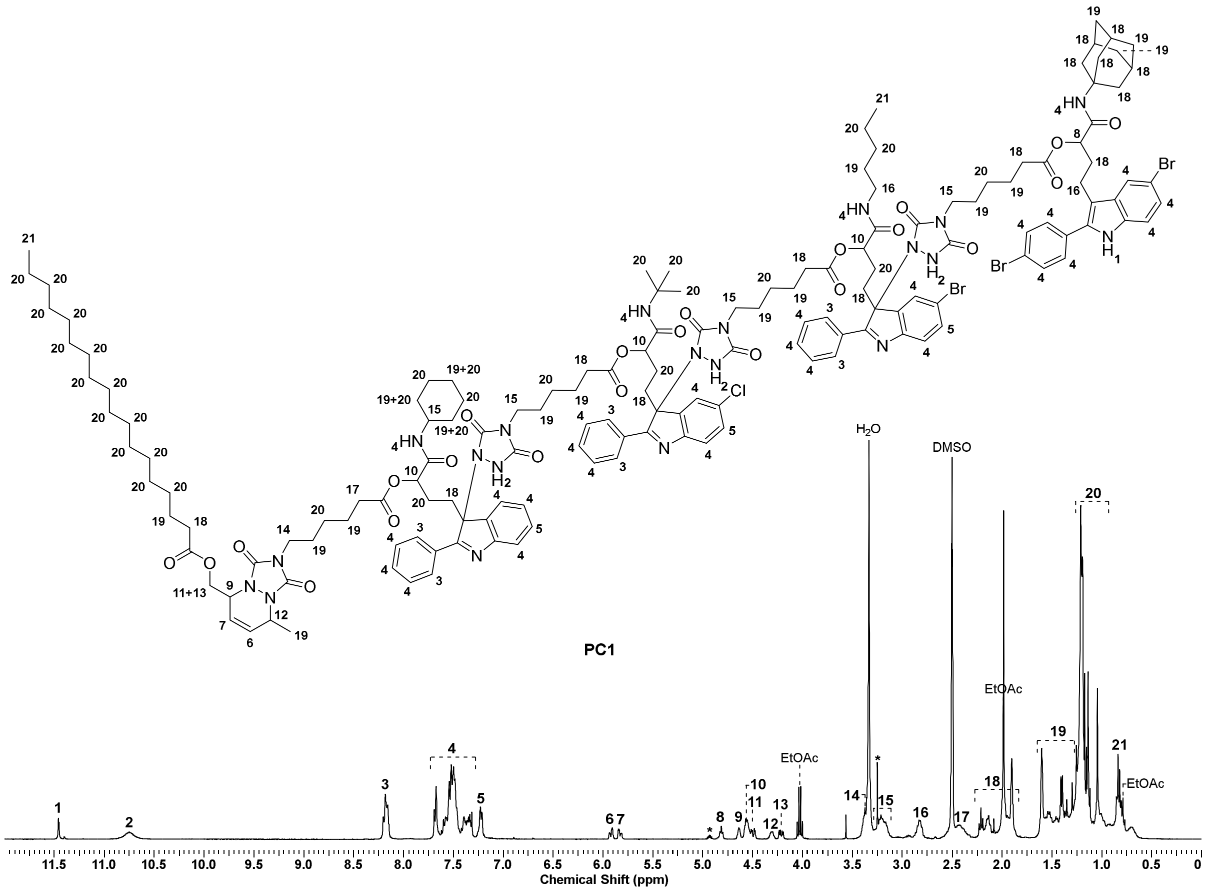

Figure S39. ^{1}H NMR spectrum (DMSO-d_6) of **PC1**.

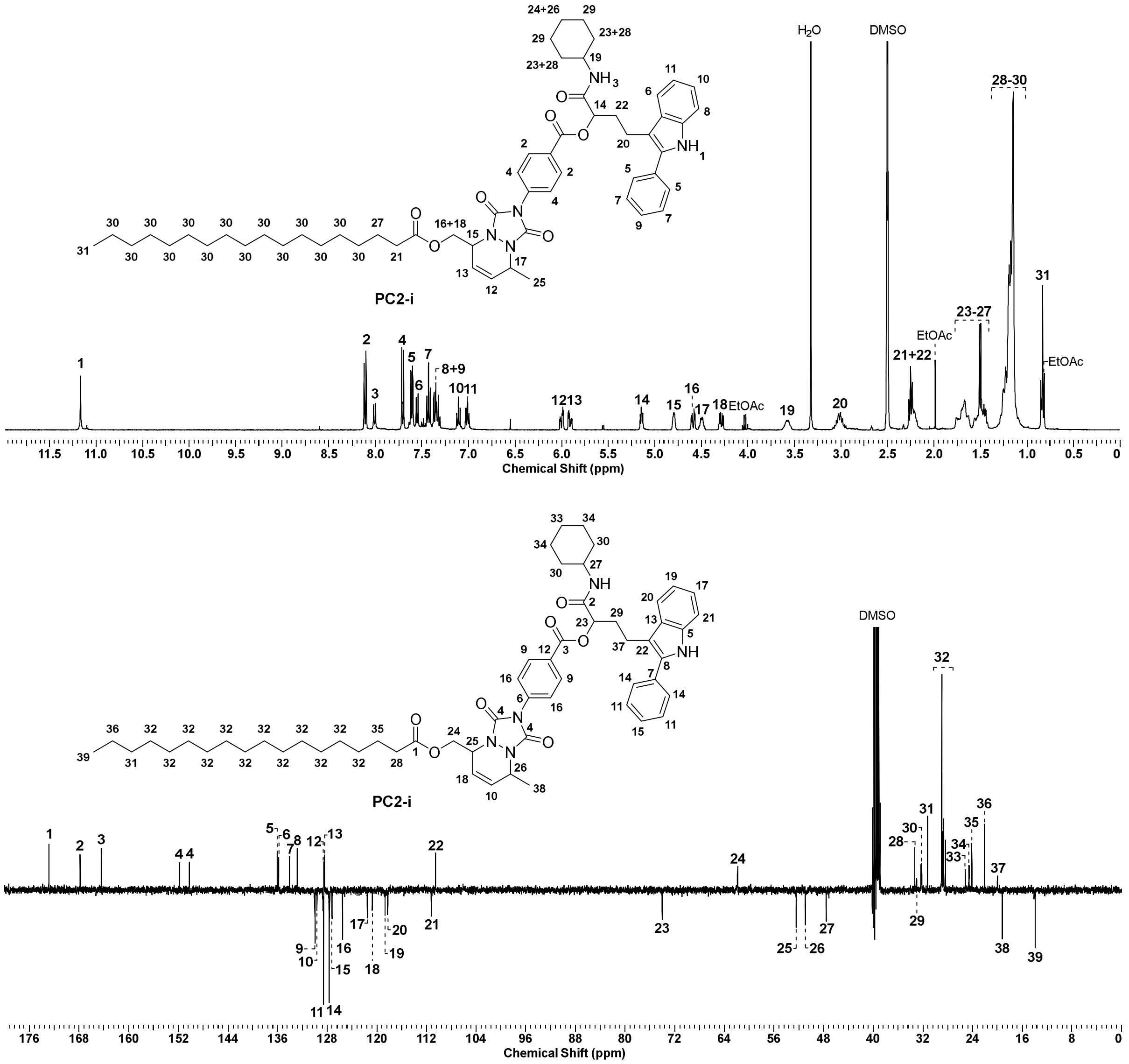

Figure S40. ^{1}H NMR (top) and ^{13}C NMR (bottom) spectrum (DMSO-d_6) of **PC2-i**.

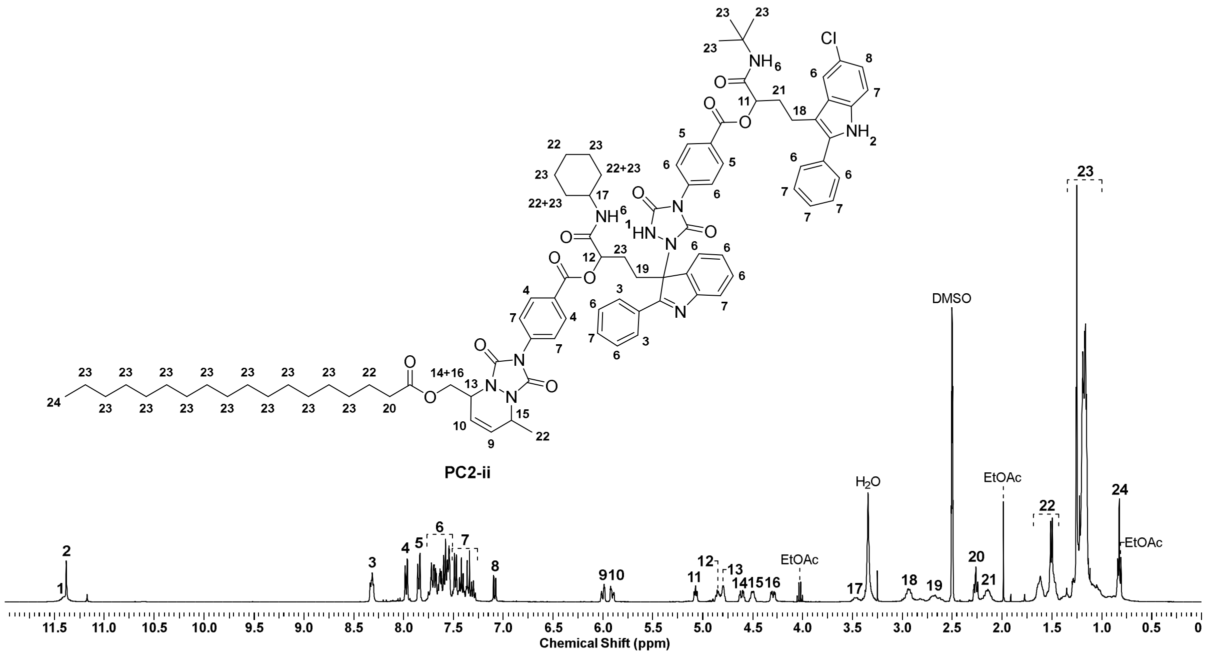

Figure S41. ^{1}H NMR spectrum (DMSO-*d*6) of **PC2-ii**.

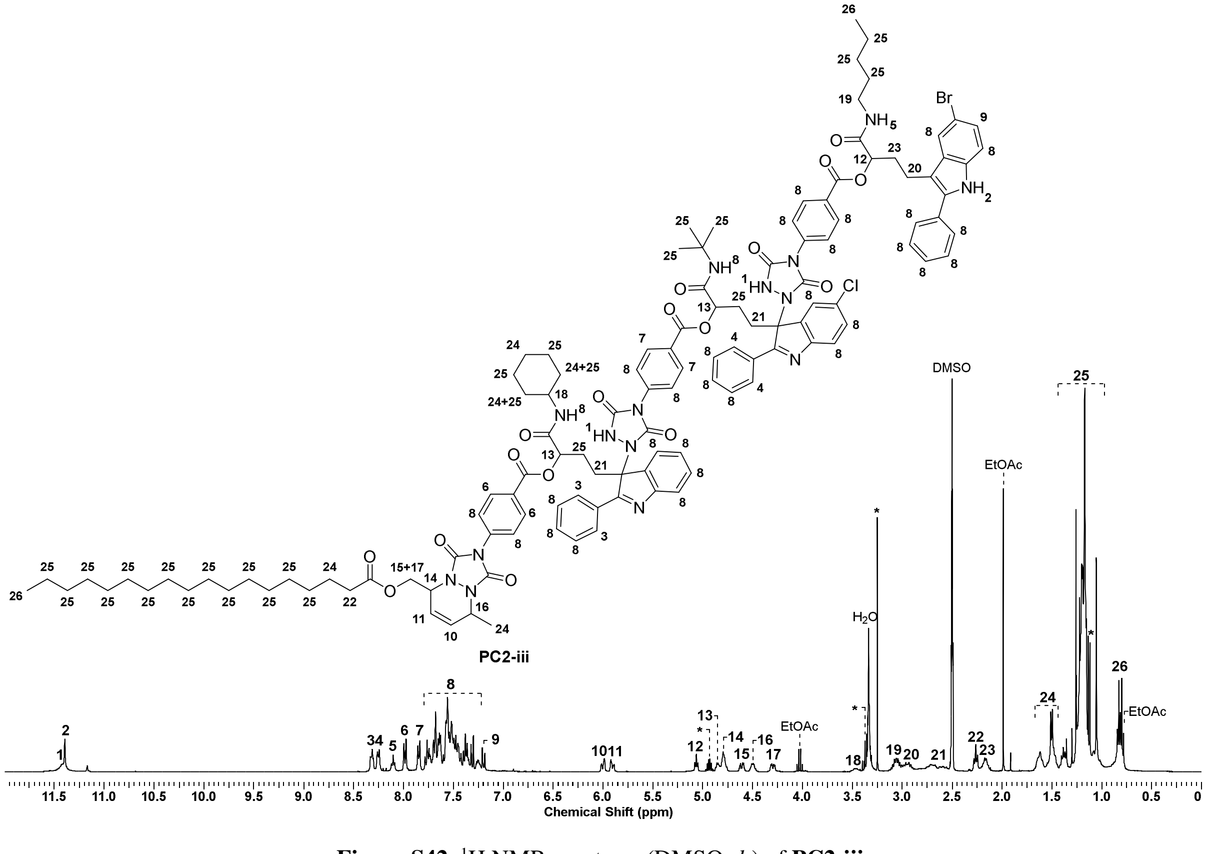

Figure S42. ^{1}H NMR spectrum (DMSO-*d*6) of **PC2-iii**.

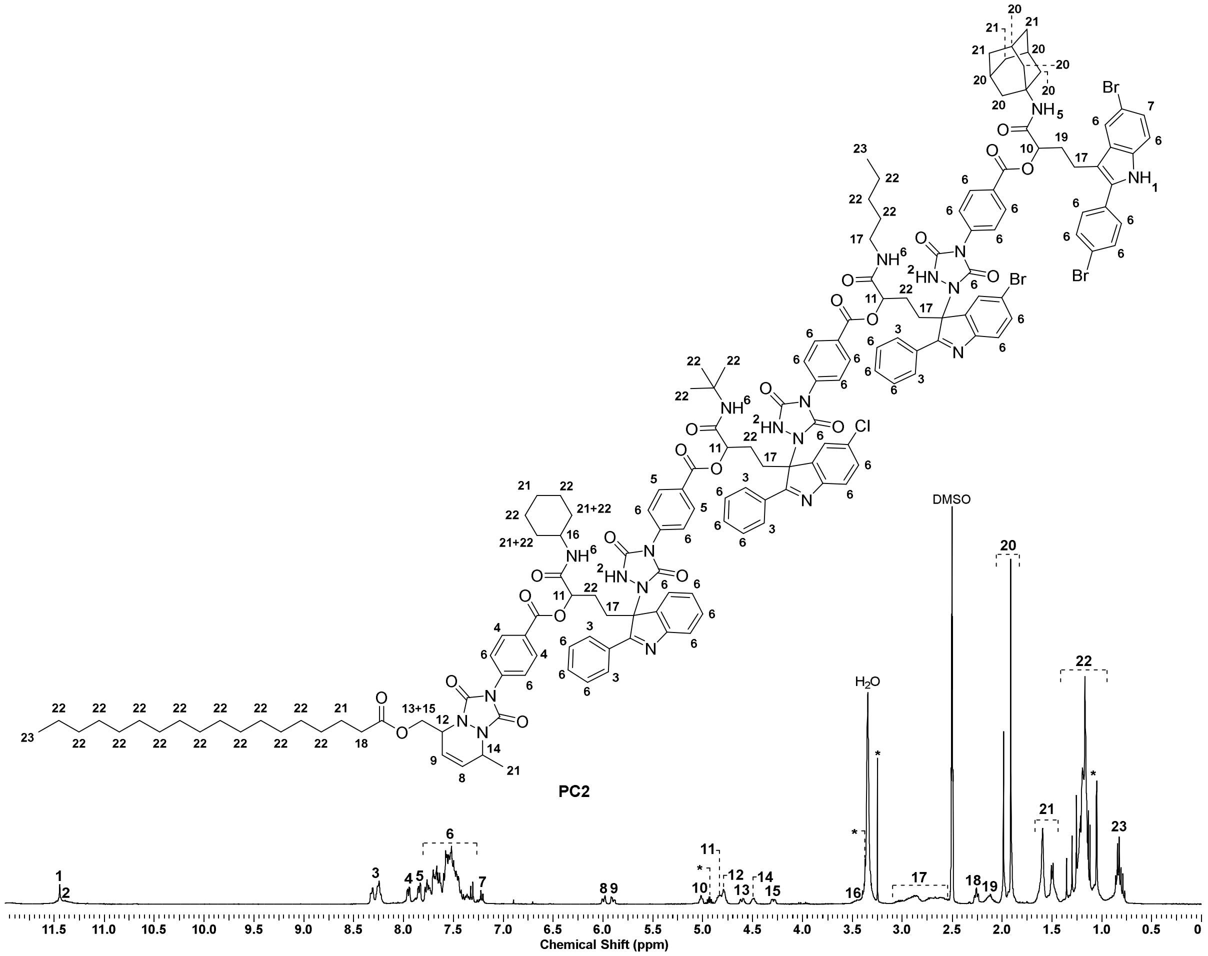

Figure S43. ^{1}H NMR spectrum (DMSO-d_6) of **PC2**.

13. References

1. J. O. Holloway, K. S. Wetzel, S. Martens, F.E. Du Prez, M. A. R. Meier, *Polym. Chem.* **2019**, *10*, 3859-3867.

2. S.-K Xiang, B. Zhang, L.-H. Zhang, Y. Cui, N. Jiao, *Chem. Commun.* **2011,** *47*, 8097-8099.

3. H. A. Houck, K. De Bruycker, S. Billiet, B. Dhanis, H. Goossens, S. Catak, V. Van Speybroeck, J.M. Winne, F.E. Du Prez, *Chem. Sci.* **2017,** *8*, 3098-3108

4. H. A. Houck, K. De Bruycker, C. Barner-Kowollik, J.M. Winne, F.E. Du Prez, *Macromolecules* **2018,** *51*, 3156-3164.
